# Learning Curves for Heterogeneous Feature-Subsampled Ridge Ensembles

**Published:** 2023-07-06

**Authors:** Benjamin S. Ruben, Cengiz Pehlevan

**Affiliations:** 1 Biophysics Graduate Program, Harvard University, Cambridge, Massachusetts 02138, USA; 2 John A. Paulson School of Engineering and Applied Sciences, Harvard University, Cambridge, Massachusetts 02138, USA; 3 Center for Brain Science, Harvard University, Cambridge, Massachusetts 02138, USA; 4 Kempner Institute for the Study of Natural and Artificial Intelligence, Harvard University, Cambridge, Massachusetts 02138, USA

## Abstract

Feature bagging is a well-established ensembling method which aims to reduce prediction variance by training estimators in an ensemble on random subsamples or projections of features. Typically, ensembles are chosen to be homogeneous, in the sense the the number of feature dimensions available to an estimator is uniform across the ensemble. Here, we introduce heterogeneous feature ensembling, with estimators built on varying number of feature dimensions, and consider its performance in a linear regression setting. We study an ensemble of linear predictors, each fit using ridge regression on a subset of the available features. We allow the number of features included in these subsets to vary. Using the replica trick from statistical physics, we derive learning curves for ridge ensembles with deterministic linear masks. We obtain explicit expressions for the learning curves in the case of equicorrelated data with an isotropic feature noise. Using the derived expressions, we investigate the effect of subsampling and ensembling, finding sharp transitions in the optimal ensembling strategy in the parameter space of noise level, data correlations, and data-task alignment. Finally, we suggest variable-dimension feature bagging as a strategy to mitigate double descent for robust machine learning in practice.

## I. INTRODUCTION

Ensembling methods, where one combines predictions from multiple predictors to achieve a stronger prediction, are ubiquitous in machine learning practice [1]. A popular class of ensembling methods (known as attribute bagging [2] as well as the random subspace method [3]) are based on feature subsampling [2–6], where each predictor has access to only a subset of data features, are independently trained on those features, and their predictions are combined to achieve a stronger prediction. For example, the popular random forest method makes use of this strategy [3, 7]. An advantage of these methods is that they allow parallel processing. For example, Feature-Distributed Machine Learning, combine independent predictions made by agents who only see subsets of available features [8].

While commonly used in practice, a theoretical understanding of ensembling via feature subsampling is not well developed. Here, we provide an analysis of this technique in the case of feature-subsampled linear ridge regression using methods from statistical physics [9–12]. This allows us to obtain analytical expressions for typical case performance of feature-subsampled linear ridge regression. Analysis of these equations under special cases reveal interesting phenomena involving effects of noise, regularization, and subsampling on prediction performance.

Our findings relate to double-descent [13, 14], which results from over-fitting to noise and poses a serious problem for practical machine learning. Regularization is commonly used to mitigate double descent, however optimal regularization strength depends on data and noise levels [15, 16]. Our theory reveals an alternative strategy. We observe that subsampling shifts the location of a predictor's sample-wise double-descent peak [14, 16, 17]. An interesting consequence of this is that if the predictors are heterogeneous in the number of features they see, they will go through double-descent at different sample-sizes. Therefore, bagging them will lead a mitigation of double-descent, as when one predictor fails, the others will compensate with accurate predictions.

In summary, we make the following original contributions:

- Using the replica trick from statistical physics [9, 11], we derive the generalization error of ensembled least-squares ridge regression with random structured Gaussian data, deterministic feature maps, and a noisy linear teacher function. Our derivation allows for heterogeneity in the rank of the feature maps of the ensemble members.
- We derive explicit formulas which demonstrate that subsampling alters the interpolation threshold of ridge regression.
- We demonstrate benefits of heterogeneous ensembling as a robust method for mitigating double-descent.
- We analyze the role of data correlations, readout noise, and data-task alignment in determining the optimal ensembling strategy in a tractable special case.

### Related works:

A substantial body of work has elucidated the behavior of linear predictors for a variety of feature maps [13, 16, 18–29]. Several recent works have extended this research to characterize the behavior of ensembled regression using solvable models [25, 30, 31]. Ref. [30] derives expressions for the generalization error of generalized linear models, of which ridge ensembles are a special case, in terms of the solutions to a set of self-consistent equations. However, [30] and [25] focus their analysis on the case of isotropic data and Gaussian random masks of *homogeneous* dimensionality. In contrast, we explicitly consider learning from correlated data by ensembles with heterogeneous readout dimensionality. Our work focuses on the effect of feature-wise subsampling. Additional recent works study the performance of ridge ensembles with example-wise subsampling [32, 33] and simultaneous subsampling of features and examples [31]. These works find that subsampling behaves as an implicit regularization, and prove equivalences between optimal ensembling and optimal regularization. In a similar vein, we consider here ensembling as a safeguard against insufficient regularization. Methods from statistical physics have long been used for machine learning theory [10–12]. Relevant work in this domain include [34] which studied ensembling by data-subsampling in linear regression.

## II. LERANING CURVES FOR ENSEMBLED RIDGE REGRESSION FROM THE REPLICA METHOD

We consider noisy ensembled ridge regression in the setting where ensemble members are trained independently on masked versions of the available features. We derive our main analytical formula for generalization error of ensembled linear regression, as well as analytical expressions for generalization error in the special case of subsampling of equicorrelated features. Later sections illustrate the implications of the derived formulas.

### A. Problem Setup

Consider a training set $𝒟={\left\{{\overset{-}{\psi }}_{\mu },y^{\mu }\right\}}_{\mu =1}^{P}$ of size P. The training examples ${\overset{-}{\psi }}_{\mu }\in R^{M}$ are drawn from a Gaussian distribution with Gaussian feature noise: ${\overset{-}{\psi }}_{\mu }=\psi_{\mu }+\sigma_{\mu }$, where $\psi_{\mu }\sim 𝒩\left(0,\Sigma_{s}\right)$ and $\sigma_{\mu }\sim 𝒩\left(0,\Sigma_{0}\right)$. Data and noise are drawn i.i.d. so that $E\left[\psi_{\mu }\psi_{\nu }^{⊤}\right]=\delta_{\mu \nu }\Sigma_{s}$ and $E\left[\sigma_{\mu }\sigma_{\nu }^{⊤}\right]=\delta_{\mu \nu }\Sigma_{0}$. Labels are generated from a noisy teacher function $y_{\mu }=\frac{1}{\sqrt{M}}w^{*⊤}\psi_{\mu }+\epsilon^{\mu }$ where $\epsilon^{\mu }\sim 𝒩\left(0,\zeta^{2}\right)$. Label noises are drawn i.i.d. so that $E\left[\epsilon^{\mu }\epsilon^{\nu }\right]=\delta_{\mu \nu }\zeta^{2}$.

We seek to analyze the quality of predictions which are averaged over an ensemble of ridge regression models, each with access to a subset of the features. We consider k linear predictors with weights ${\overset{ˆ}{w}}_{r}\in R^{N_{r}},r=1,\ldots ,k$. Critically, we allow $N_{r}\neq N_{r^{\prime }}$ for $r\neq r^{\prime }$, which allows us to introduce *structural* heterogeneity into the ensemble of predictors. A forward pass of the model is given as:

(1) $$f(\psi )=\frac{1}{k}\sum_{r=1}^{k}\;f_{r}(\psi ),\;f_{r}(\psi )=\frac{1}{\sqrt{N_{r}}}{\overset{ˆ}{w}}_{r}^{⊤}A_{r}(\psi +\sigma )+\xi_{r}.$$

The model's prediction f(ψ) is an average over k linear predictors. The "measurement matrices" $A_{r}\in R^{N_{r}\times M}$ act as linear masks restricting the information about the features available to each member of the ensemble. Subsampling may be implemented by choosing the rows of each $A_{r}$ to coincide with the rows of the identity matrix - the row indices corresponding to indices of the sampled features. The feature noise $\sigma \sim 𝒩\left(0,\Sigma_{0}\right)$ and the readout noises $\xi_{r}\sim 𝒩\left(0,\eta_{r}^{2}\right)$, are drawn independently at the execution of each forward pass of the model. Note that while the feature noise is shared across the ensemble, readout noise is drawn independently for each readout: $E\left[\xi_{r}\xi_{r^{\prime }}\right]=\delta_{rr^{\prime }}\eta_{r}^{2}$.

The weight vectors are trained separately in order to minimize a regular least-squares loss function with ridge regularization:

(2) $${\overset{ˆ}{w}}_{r}=\underset{w_{r}\in R^{N_{r}}}{argmin}\left[\sum_{\mu =1}^{P}\;\;{\left(\frac{1}{\sqrt{N_{r}}}w_{r}^{⊤}A_{r}{\overset{-}{\psi }}_{\mu }+\xi_{r}^{\mu }-y_{\mu }\right)}^{2}+\lambda \left|w_{r}^{2}\right|\right]$$

Here $\left\{\xi_{r}^{\mu }\right\}$ represents the readout noise which is present during training, and independently drawn: $\xi_{r}^{\mu }\sim 𝒩\left(0,\eta_{r}^{2}\right)$, $E\left[\xi_{r}^{\mu }\xi_{r}^{\nu }\right]=\eta_{r}^{2}\delta_{\mu \nu }$. As a measure of model performance, we consider the generalization error, given by the mean-squared-error (MSE) on ensemble-averaged prediction:

(3) $$E_{g}(𝒟)=\left\langle {\left(f(\psi )-\frac{1}{\sqrt{M}}w^{*⊤}\psi \right)}^{2}\right\rangle$$

Here, the angular brackets represent an average over the data distribution and noise: $\psi \sim 𝒩\left(0,\Sigma_{s}\right),\sigma \sim 𝒩\left(0,\Sigma_{0}\right)$, $\xi_{r}\sim 𝒩\left(0,\eta_{r}^{2}\right)$. The generalization error depends on the particular realization of the dataset 𝒟 through the learned weights $\left\{{\overset{ˆ}{w}}^{*}\right\}$. We may decompose the generalization error as follows:

(4) $$E_{g}(𝒟)=\frac{1}{k^{2}}\sum_{r,r^{\prime }=1}^{k}\;\;E_{rr^{\prime }}(𝒟)$$

(5) $$E_{rr^{\prime }}(𝒟)\equiv \frac{1}{M}\left[{\left(\frac{1}{\sqrt{\nu_{rr}}}A_{r}^{⊤}{\overset{ˆ}{w}}_{r}-w^{*}\right)}^{⊤}\Sigma_{s}\left(\frac{1}{\sqrt{\nu_{r^{\prime }r^{\prime }}}}A_{r^{\prime }}^{⊤}{\overset{ˆ}{w}}_{r^{\prime }}-w^{*}\right)\right.\;\left.+\frac{1}{\sqrt{\nu_{rr}\nu_{r^{\prime }r^{\prime }}}}{\overset{ˆ}{w}}_{r}^{⊤}A_{r}\Sigma_{0}A_{r^{\prime }}^{⊤}{\overset{ˆ}{w}}_{r^{\prime }}+M\delta_{rr^{\prime }}\eta_{r}^{2}\right]$$

Computing the generalization error of the model is then a matter of calculating $E_{rr^{\prime }}$ in the cases where $r=r^{\prime }$ and $r\neq r^{\prime }$. Furthermore, in the asymptotic limit we consider, we expect that the generalization error concentrates over randomly drawn datasets 𝒟.

### B. Main Result

We calculate the generalization error using the replica trick from statistical physics. The result of our calculation is stated in proposition 1. The proof is lengthy, and can be found in the SI.

**Proposition 1.** *Consider the ensembled ridge regression problem described in* Section II A. *Consider the asymptotic limit where* $M,P,\left\{N_{r}\right\}\to \infty$ *while the ratios* $\alpha =\frac{P}{M}$ *and* $\nu_{rr}=\frac{N_{r}}{M},r=1,\ldots ,k$ *remain fixed. Define the following quantities*:

(6) $${\tilde{\Sigma }}_{rr^{\prime }}\equiv \frac{1}{\sqrt{\nu_{rr}\nu_{r^{\prime }r^{\prime }}}}A_{r}\left[\Sigma_{s}+\Sigma_{0}\right]A_{r^{\prime }}^{⊤}$$

(7) $$G_{r}\equiv I_{N_{r}}+{\overset{ˆ}{q}}_{r}{\tilde{\Sigma }}_{rr}$$

(8) $$\gamma_{rr^{\prime }}\equiv \frac{\alpha }{\left.M\left(\lambda +q_{r}\right)\left(\lambda +q_{r^{\prime }}\right)\right)}tr\left[G_{r}^{-1}{\tilde{\Sigma }}_{rr^{\prime }}G_{r^{\prime }}^{-1}{\tilde{\Sigma }}_{r^{\prime }r}\right]$$

*Then the average generalization error may be written as:*

(9) $${\left\langle E_{g}(𝒟)\right\rangle }_{𝒟}=\frac{1}{K^{2}}\sum_{r,r^{\prime }=1}^{K}\;{\left\langle E_{rr^{\prime }}(𝒟)\right\rangle }_{𝒟},$$

*where*

(10) $${\left\langle E_{rr^{\prime }}(𝒟)\right\rangle }_{𝒟}=\frac{\gamma_{rr^{\prime }}\zeta^{2}+\delta_{rr^{\prime }}\eta_{r}^{2}}{1-\gamma_{rr^{\prime }}}+\frac{1}{1-\gamma_{rr^{\prime }}}\left(\frac{1}{M}w^{*⊤}\Sigma_{s}w^{*}\right)\;\;-\frac{1}{M\left(1-\gamma_{rr^{\prime }}\right)}w^{*⊤}\Sigma_{s}\left[\frac{1}{\nu_{rr}}{\overset{ˆ}{q}}_{r}A_{r}^{⊤}G_{r}^{-1}A_{r}+\frac{1}{\nu_{r^{\prime }r^{\prime }}}{\overset{ˆ}{q}}_{r^{\prime }}A_{r^{\prime }}^{⊤}G_{r^{\prime }}^{-1}A_{r^{\prime }}\right]\Sigma_{s}w^{*}\;\;+\frac{{\overset{ˆ}{q}}_{r}{\overset{ˆ}{q}}_{r^{\prime }}}{M\left(1-\gamma_{rr^{\prime }}\right)}\frac{1}{\sqrt{\nu_{rr}\nu_{r^{\prime }r^{\prime }}}}w^{*⊤}\Sigma_{s}A_{r}^{⊤}G_{r}^{-1}{\tilde{\Sigma }}_{rr^{\prime }}G_{r^{\prime }}^{-1}A_{r^{\prime }}\Sigma_{s}w^{*}$$

*where the pairs of order parameters* $\left\{q_{r},{\overset{ˆ}{q}}_{r}\right\}$ *for* r=1,…,K, *satisfy the following self*-*consistent saddle-point equations*

(11) $${\overset{ˆ}{q}}_{r}=\frac{\alpha }{\lambda +q_{r}},\;q_{r}=\frac{1}{M}tr\left[G_{r}^{-1}{\tilde{\Sigma }}_{rr}\right].$$

*Proof*. We calculate the terms in the generalization error using the replica trick from the statistical physics of disordered systems. The full derivation may be found in the supplemental material. □

We make several remarks on this result:

*Remark 1*. This is a highly general result which applies to any selection of linear masks $\left\{A_{r}\right\}$. However, we will focus on the case where the $\left\{A_{r}\right\}$ implement subsampling of the feature neurons.
*Remark 2*. Our result reduces to the results of [35] when k=1 and η=0, and may be obtained as a special case of [36] in this limit. In the case where all readout weights have the same dimension $N_{r}=N,r=1,\ldots ,k$, this result may be obtained as a special case of the results of [30]. The novelty in our derivation (and subsequent analysis) is to consider heterogeneity in the values of $N_{r}$.
*Remark* 3. The replica trick [37] is a non-rigorous but standard heuristic in the study of disordered systems. We confirm our results in simulations.

**FIG. 1.**
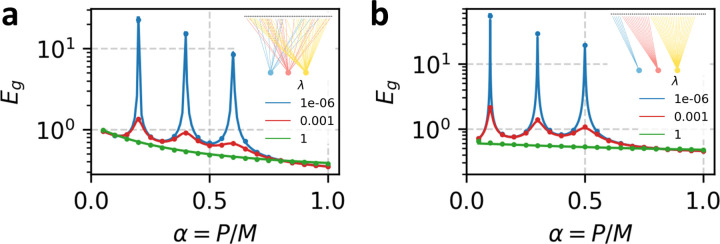
Comparison between numerically calculated generalization error and theoretical prediction. Dots show results of numerical experiment. Lines are theoretical prediction. (a) Numerical experiment with ${\left[\Sigma_{s}\right]}_{ij}=.8^{\left|i-j\right|},{\left[\Sigma_{0}\right]}_{ij}=\frac{1}{10}(0.3{)}^{\left|i-j\right|}$, ζ=0.1,η=0.2. We set k=3 with $\nu_{1}=0.2,\nu_{2}=0.4,\nu_{3}=0.6$. Subsets of feature neurons accessed by each readout are drawn randomly and are permitted to overlap (see inset). Circular markers show the result of numerical experiment with M=2000 feature neurons averaged over 100 trials. Curve shows theoretical prediction, obtained by solving the saddle-point equations 11 numerically. Theory and experiment conducted with a fixed ground-truth readout $w^{*}$ drawn randomly from an isotropic standard Gaussian distribution (b) Numerical experiment with ${\left[\Sigma_{s}\right]}_{ij}=(0.6)\delta_{ij}+0.4,{\left[\Sigma_{0}\right]}_{ij}=.1\delta_{ij},\zeta =0.1,\eta =0.1$. Ground truth weights are randomly sampled in each trial as in eq. 12 with ρ=.3. We set k=3 with $\nu_{1}=0.1,\nu_{2}=0.3,\nu_{3}=0.5$. Subsets of feature neurons accessed by each readout are are mutually exclusive (see inset). Circular markers show the result of numerical experiment with M=5000 feature neurons averaged over 100 trials. Error bars show the standard error of the mean, and are smaller than the markers. Curve shows analytical prediction obtained in the case of equicorrelated features.

In Figure 1a, we confirm the result of the general calculation by comparing with numerical experiments. Experimental curves are generated by running ridge regression on randomly drawn datasets with M=2000 features and averaging over the resulting error. We use highly structured data, feature noise, label noise, and readout noise (see caption for details). Each of k=3 readouts sees a fixed but randomly drawn subset of features. Theory curves are calculated by solving the fixed-point equations 11 numerically for the chosen $\Sigma_{s},\Sigma_{0}$ and ${\left\{A_{r}\right\}}_{r=1}^{k}$ then plugging the resulting order parameters into eq. 10.

### C. Equicorrelated Data

Our general result allows the freedom to tune many important parameters of the learning problem: the correlation structure of the dataset, the number of ensemble members, the scales of noise, etc. However, the derived expressions are rather opaque, as they depend on the solution to a set of in general analytically intractable self-consistent equations for the order parameters. In order to better understand the phenomena captured by these expressions, we simplify them in the tractable special case in which features of the data are equicorrelated:

**Proposition 2.** *Consider the ensembled ridge regression problem described in* section II A, *and the result of proposition 1. Consider the special case in which we select the following parameters*:

(12) $$w^{*}=\sqrt{1-\rho^{2}}P_{⊥}w_{0}^{*}+\rho 1_{M}$$

(13) $$w_{0}^{*}\sim 𝒩\left(0,I_{M}\right)$$

(14) $$\Sigma_{s}=s\left[(1-c)I_{M}+c1_{M}1_{M}^{⊤}\right]$$

(15) $$\Sigma_{0}=\omega I_{M}$$

*with* c∈[0,1],ρ∈[-1,1]. *A label noise scale* ζ≥0 *and readout noise scales* $\eta_{r}\geq 0$ *are permitted. Here* $P_{⊥}=I_{M}-\frac{1}{N}1_{M}1_{M}^{⊤}$ *is a projection matrix which removes the component of* $w_{0}^{*}$ *which is parallel to* $1_{M}$. *The measurement matrices* ${\left\{A_{r}\right\}}_{r=1}^{k}$ *have rows consisting of distinct one-hot vectors so that each of the* k *readouts has access to a subset of* $N_{r}=\nu_{rr}M$ *features*. *For* $r\neq r^{\prime }$, *denote by* $n_{rr^{\prime }}$ *the number of neurons sampled by both* $A_{r}$ *and* $A_{r^{\prime }}$ *and let* $\nu_{rr^{\prime }}\equiv n_{rr^{\prime }}/M$ *remain fixed as* M→∞.

*In this case, we may obtain fully analytical formulas for the generalization error as follows. First define the following quantities*:

(16) $$a\equiv s(1-c)+\omega \;S_{r}\equiv \frac{{\overset{ˆ}{q}}_{r}}{\nu_{rr}+a{\overset{ˆ}{q}}_{r}},\;\gamma_{rr^{\prime }}\equiv \frac{a^{2}\nu_{rr^{\prime }}S_{r}S_{r^{\prime }}}{\alpha }$$

*The terms of the decomposed generalization error may then be written:*

(17) $${\left\langle E_{rr^{\prime }}\right\rangle }_{𝒟,w_{0}^{*}}=\frac{1}{1-\gamma_{rr^{\prime }}}\left(\left(1-\rho^{2}\right)I_{rr^{\prime }}^{0}+\rho^{2}I_{rr^{\prime }}^{1}\right)+\frac{\gamma_{rr^{\prime }}\zeta^{2}+\delta_{rr^{\prime }}\eta_{r}^{2}}{1-\gamma_{rr^{\prime }}}$$

*where we have defined*

(18) $$I_{rr^{\prime }}^{0}\equiv s(1-c)\left(1-s(1-c)\nu_{rr}S_{r}-s(1-c)\nu_{r^{\prime }r^{\prime }}S_{r^{\prime }}+as(1-c)\nu_{rr^{\prime }}S_{r}S_{r^{\prime }}\right)$$

(19) $$\begin{array}{r} I_{rr^{\prime }}^{1}\equiv \left\{\begin{array}{ll} \frac{s(1-c)\left(\nu_{rr^{\prime }}-\nu_{rr}\nu_{r^{\prime }r^{\prime }}\right)+\omega \nu_{rr^{\prime }}}{\nu_{rr}\nu_{r^{\prime }r^{\prime }}} & \text{ if }0<c\leq 1\\ I_{rr^{\prime }}^{0} & \text{ if }c=0 \end{array}\right. \end{array}$$

*and where the order parameters* $\left\{q_{r},{\overset{ˆ}{q}}_{r}\right\}$ *may be obtained analytically as the solution (with* $q_{r}>0$*) to the following quadratic system of equations:*

(20) $$q_{r}=\frac{a\nu_{rr}}{\nu_{rr}+a{\overset{ˆ}{q}}_{r}}\;,\;{\overset{ˆ}{q}}_{r}=\frac{\alpha }{\lambda +q_{r}}$$

*In the "ridgeless" limit where* λ→0, *we may make the following simplifications:*

(21) $$S_{r}\;\to \frac{2\alpha }{a\left(\alpha +\nu_{rr}+\left|\alpha -\nu_{rr}\right|\right)}$$

(22) $$\gamma_{rr^{\prime }}\;\to \frac{4\alpha \nu_{rr^{\prime }}}{\left(\alpha +\nu_{rr}+\left|\alpha -\nu_{rr}\right|\right)\left(\alpha +\nu_{r^{\prime }r^{\prime }}+\left|\alpha -\nu_{r^{\prime }r^{\prime }}\right|\right)}$$

*Proof*. Simplifying the fixed-point equations and generalization error formulas in this special case is an exercise in linear algebra. The main tools used are the Sherman-Morrison formula [38] and the fact that the data distribution is isotropic in the features so that the form of ${\tilde{\Sigma }}_{rr}$ and ${\tilde{\Sigma }}_{rr^{\prime }}$ depend only on $N_{r},N_{r^{\prime }}$, and $n_{rr^{\prime }}$. Thus, the result depends only on the values of $\left\{\nu_{rr^{\prime }}\right\}$ and not the identities of the subsampled features. To aid in computing the necessary matrix contractions we developed a custom Mathematica package which handles block matrices of symbolic dimension, with blocks containing matrices of the form $M=c_{1}I+c_{2}11^{⊤}$. This package and the Mathematica notebook used to derive these results will be made available online (see SI) □

In this tractable special case, c∈[0,1] is a parameter which tunes the strength of correlations between features of the data. When c=0, the features are independent, and when c=1 the features are always equivalent. s sets the overall scale of the features and ρ tunes the alignment of the ground truth weights with the special direction in the covariance matrix. We refer to ρ as the "task alignment", and it can be thought of as a simple proxy for the "task-model alignment" [16] or "code-task alignment" [39]. In Figure 1b, we test these results by comparing the theoretical expressions for generalization error with the results of numerical experiments, finding perfect agreement. Note that in this case, both theory and experiment are averaged over ground-truth weights as well as datasets.

### D. Subsampling shifts the double-descent peak of a linear predictor.

Consider the equicorrelated data model in the isotropic limit (c=0). Consider a single linear regressor (k=1) which connects to a subset of N=νM features. In the ridgeless limit where regularization λ→0, and without readout noise or feature noise (η=ω=0), the generalization error is given by equation 17 with $\nu_{rr}=\nu ,s=1,\eta_{r}=\omega =0$ in the λ→0 limit:

(23) $${\left\langle E_{g}\right\rangle }_{𝒟,w^{*}}=\left\{\begin{array}{ll} \frac{\nu }{\nu -\alpha }\left[\left(1-\nu_{rr}\right)+\frac{1}{\nu_{rr}}(\alpha -\nu {)}^{2}\right]+\frac{\alpha }{\nu -\alpha }\zeta^{2}, & \text{ if }\alpha <\nu \\ \frac{\alpha }{\alpha -\nu }[1-\nu ]+\frac{\nu }{\alpha -\nu }\zeta^{2}, & \text{ if }\alpha >\nu \end{array}\right\}$$

**FIG. 2.**
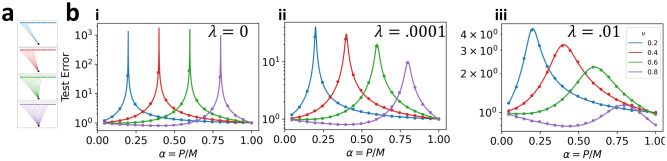
Subsampling alters the location of the double-descent peak of a linear predictor. (a) Illustrations of subsampled linear predictors with varying subsampling fraction ν. (b) Comparison between experiment and theory for subsampling linear regression on equicorrelated datasets. We choose ${\left[\Sigma_{s}\right]}_{ij}=\delta_{ij},{\left[\Sigma_{0}\right]}_{ij}=0,\zeta =0,\eta =0$, and (i) λ=0, (ii) $\lambda =10^{-4}$, (iii) $\lambda =10^{-2}$. Dots show results of numerical experiment. Lines are analytical prediction.

Double descent can arise from two possible sources of variance: explicit label noise (ζ>0) or implicit label noise induced by feature subsampling (ν<1). As $E_{g}\sim (\alpha -\nu {)}^{-1}$, we see that the generalization error diverges when α=ν. The subsampling fraction ν thus controls the sample complexity α at which the double-descent peak occurs. Intuitively, this occurs because subsampling changes the number of parameters of the regression model, and thus its interpolation threshold. To demonstrate this, we plot the learning curves for subsampled linear regression on equicorrelated data in Figure 2. While at finite ridge the test error no longer diverges when α=ν, it may still display a distinctive peak.

### E. Heterogeneous connectivity mitigates double-descent

**FIG. 3.**
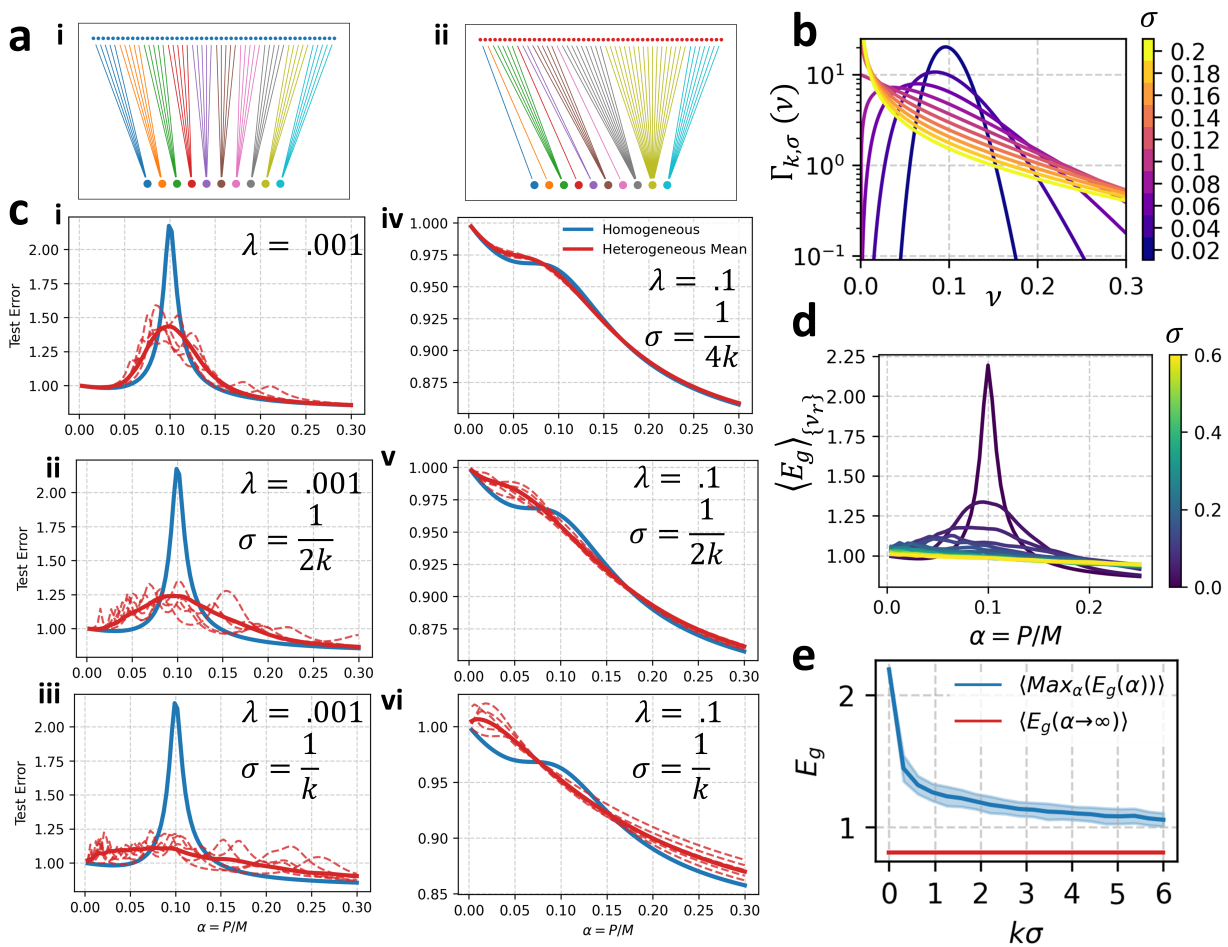
Homogeneous vs. Heterogeneous Ensembling on equicorrelated data. (a) We compare (i) homogeneous ensembling, in which each readout connects to the same number of feature neurons and (ii) heterogeneous ensembling, in which the number of feature neurons connected by a readout is drawn from a distribution. (b) We use the Gamma distribution with the convention that $\Gamma_{k,\sigma }(\nu )$ is the probability density function of the Gamma distribution with mean $k^{-1}$ and variance $\sigma^{2}$. Shown here for k=10 and σ indicated by the colorbar. (c) Generalization Error Curves for Homogeneous and Heterogeneous ensembling with k=10 and indicated values of λ and σ. Curves are calculated using analytical theory for equicorrelated data with c=0,η=0, ζ=0. Solid blue is the learning curve for homogeneous subsampling. Dotted red curves show loss curve for 5 realizations of the randomly drawn subsampling fractions ${\left\{\nu_{rr}\right\}}_{r=1}^{k}$. Solid red is the learning curve for heterogeneous ensembling averaged over 100 realizations of the subsampling fractions ${\left\{\nu_{rr}\right\}}_{r=1}^{k}$ drawn independently from $\Gamma_{k,\sigma }(\nu )$. (d) Average loss curves for heterogeneous ensembling with $k=10,\lambda =10^{-3}$, and σ indicated by the colorbar. (e) Average worst-case error and asymptotic error as a function of variance for heterogeneous ensembling. Worst-case error is calculated for each realization of the subsampling fractions as ${max}_{\alpha }\;E_{g}\left(\alpha |{\left\{\nu_{rr}\right\}}_{r=1}^{k}\right)$. Average worst-case error is the worst-case error averaged over realizations of the subsampling fractions. Shaded region shows standard deviation over realizations of the subsampling fractions.

The observed phenomenon of double-descent – over-fitting to noise in the training set near a model's interpolation threshold - poses a serious risk in practical machine-learning applications. Regularization is the canonical strategy employed to mitigate double descent. However, in order to achieve monotonic learning, the regularization parameter must be tuned to the structure of the task and the scale of the label noise [15] – no one choice for the regularization parameter can mitigate double descent for all tasks. Considering again the plots in Figure 2(b), we observe that at any value of α, the double-descent peak can be avoided with an acceptable choice of the subsampling fraction ν. This suggests another strategy to mitigate double descent: heterogeneous ensembling. Rather than training an ensemble of linear predictors, each with the same interpolation threshold, we may ensemble over predictors with a heterogeneous distribution of interpolation thresholds in the hopes that when one predictor fails, the other members of the ensemble compensate. In Figure 3, we demonstrate that in the absence of a sufficiently regularization, heterogeneous ensembling can mitigate double-descent. Specifically. We define two ensembling strategies: in homogeneous ensembling, each of the k readouts is connected to the same fraction $\nu_{rr}=\frac{1}{k}$ features. In heterogeneous ensembling, the number of features connected by each of the k readouts are drawn i.i.d. from a Gamma distribution with fixed mean 1/k and variance $\sigma^{2}$. We denote this $\nu_{rr}\sim \Gamma_{k,\sigma }$. After they are independently drawn, subsampling fractions are re-scaled so that they sum to unity: $\nu_{rr}/\sum_{r}\;\nu_{rr}\leftarrow \nu_{rr}$. This ensures fair competition, wherein the total number of readout weights utilized in homogeneous and heterogeneous ensembling are equal. Equivalently, we may consider the readout fractions $\nu_{rr}$ to be drawn from a Dirichlet distribution: $\left(\nu_{1},\ldots ,\nu_{k}\right)\sim Dir\left((\sigma k{)}^{-2},\ldots ,(\sigma k{)}^{-2}\right)$ [40]. These strategies for connecting readouts to the features are illustrated for k=10 in figures 3 a.i (homogeneous) and 3 a.ii (heterogeneous). The density of the distribution $\Gamma_{k,\sigma }(\nu )$ is plotted in figure 3b for k=10 and varying σ. In figure S1, we apply these ideas to a classification task on the CIFAR-10 dataset. We find that in this nonlinear setting, heterogeneous ensembling prevents catastrophic over-fitting, leading to monotonic learning curves without regularization (see SI for details).

In figure 3c, we use our analytical theory of equicorrelated data (see eqs. 17) to compare the performance of homogeneous and heterogeneous ensembling with k=10. We find that for an under-regularized predictor, (3c.i, c.ii, c.iii) heterogeneous ensembling reduces the height of the double-descent peak. At larger regularization (3c.iv, c.v, c.vi), homogeneous and heterogeneous ensembling perform similarly. We quantify the extent of double-descent through the worst-case error ${max}_{\alpha }\;\left(E_{g}(\alpha )\right)$. We find that as σ increases, the worst-case error decreases monotonically at no cost to the asymptotic error $E_{g}(\alpha \to \infty )$ (see Fig. 3d,e).

### F. Data correlations, readout noise, and task structure determine optimal ensemble size

We now ask whether ensembling is a fruitful strategy – i.e. whether it is preferable to have a single, fully connected readout or multiple sparsely connected readouts. Intuitively, the presence of correlations between features permits subsampling, as measurements from a subset of neurons will also confer information about the state of the others. In addition, ensembling over multiple readouts can average out the readout noise. To quantify these notions, we consider the special case of ensembling over k readouts, each connecting the same fraction $\nu_{rr}=\nu =\frac{1}{k}$ of features in an equicorrelated code with correlation strength c and readout noise scale η, and task alignment ρ. We set the label noise, feature noise, and overlap between readouts to zero $\left(\zeta =0,\omega =0,\nu_{rr^{\prime }}=0\right.$ when $\left.r\neq r^{\prime }\right)$. In the ridgeless limit, we can then express the error as : $E_{g}(k)=s(1-c)F(H,k,\rho ,\alpha )$, where $H\equiv \frac{\eta^{2}}{s(1-c)}$ is an effective inverse signal-to-noise ratio and F(H,k,ρ,α) is a rational function of its arguments (see SI for full expressions). Therefore, given fixed parameters s,c,ρ,α, the value $k^{*}$ which minimizes error depends on η,s, and c only through the ratio H.

**FIG. 4.**
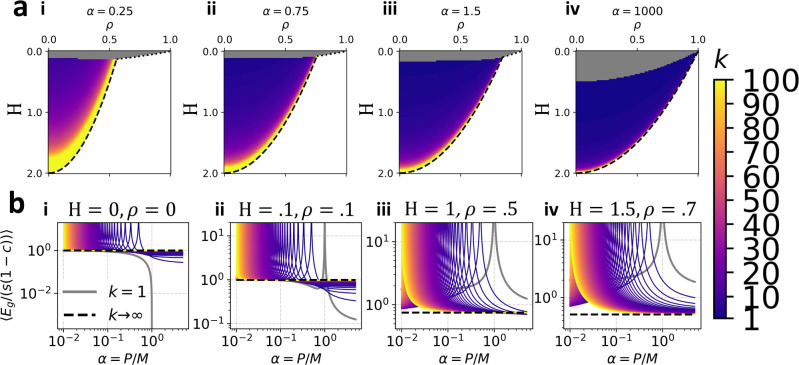
Noise level and data correlation strength determine optimal readout strategy: Using analytical theory (see eq. 17), we calculate the generalization error of linear predictors on equicorrelated data $\left({\left[\Sigma_{s}\right]}_{ij}=(1-c)\delta_{i}j+c,0<c\leq 1\right)$ with readout noise with variance $\eta^{2}$. Ground truth weights are drawn as in eq. 12. For convenience, we set λ=0, though results are qualitatively similar with small finite ridge. We consider k readouts, each connecting a fraction ν=1/k of the feature neurons, so that the total number of readout weights is conserved. (a) Phase diagrams of optimal k in the parameter space of task alignment ρ and the inverse effective signal-to-noise ratio $H\equiv \frac{\eta^{2}}{s(1-c)}$. Color indicates the optimal number of readouts $k^{*}$, with gray indicating $k^{*}=1$ and white indicating $k^{*}=\infty$ We consider (i) α=0.25, (ii) α=0.75, (iii) α=1.5, (iv) $\alpha =10^{3}$. Black lines are analytically derived phase boundaries between regions of parameter space where finite optimal $k^{*}$ exists and where $k^{*}=\infty$. Dotted black lines are phase boundaries of the type where $k^{*}$ jumps discontinuously from 1 to ∞. Dashed black lines are phase boundaries of the type where $k^{*}\to \infty$ from one side and $k^{*}=\infty$ on the other. (b) for three choices of the parameters (H,ρ) we plot the learning curve for ensembled linear regression for a variety of k values (see colorbar), as well as k=∞, indicated by the dotted black line. Depending on the region of parameter space, the optimal readout strategy may be to select k*=1,1<k*<∞, or k*=∞.

Using our analytical theory, we plot the optimal number of readouts k in the parameter space of H and ρ (see Fig. 4a). The resulting phase diagrams are naturally divided into three regions. In the signal-dominated phase a single fully-connected readout is optimal $\left(k^{*}=1\right)$. In an intermediate phase, $1<k^{*}<\infty$ minimizes error. And in a noise-dominated phase $k^{*}=\infty$. The boundary between the signal-dominated and noise-dominated phases (dotted lines in 4a) can be written $H=\left(1-\frac{1}{\alpha }\right)\left(1-\rho^{2}\right)$ when α>1 and $H=\alpha (1-\alpha )\left(1-\rho^{2}\right)$ when α<1. The boundary between the intermediate and noise-dominated phases (dashed lines in 4a) can be written $H=2-\left(2+\frac{1}{\alpha }\right)\rho^{2}$. As is evident in these phase diagrams, an increase in H causes an increase in $k^{*}$. This can occur because of a decrease in the signal-to-readout noise ratio $s/\eta^{2}$, or through an increase in the correlation strength c. An increase in ρ also leads to an increase in $k^{*}$, indicating that ensembling is more effective when there is alignment between the structure of the data and the task. Learning curves from each of these phases for varying k are plotted in Fig. 4b. The resulting shifts in the location of the double-descent peak resemble those observed in practice for ensembling methods applied to linear classifiers [6].

## III. CONCLUSION

In this paper, we provided a theory of feature-subsampled ensembling techniques focusing on feature-subsampled linear ridge regression. Our technique was the replica method from statistical physics which led us to derive an analytical formula for the typical case generalization error in the aforementioned setting. We solved these equations for a special case which revealed many interesting phenomena.

One of these phenomena relate to double descent [13, 14]. In most machine learning applications, the size of the dataset is known at the outset and suitable regularization may be determined to mitigate double descent, either by selecting a highly over-parameterized model [13] or by cross-validation techniques (see for example [19]). However, in contexts where a single network architecture is designed for an unknown task or a variety of tasks with varying structure and noise levels, heterogeneous ensembling may be used to smooth out the perils of double-descent. Our analysis of ensembling in noisy neural networks suggests that an ensembling approach may be useful in improving the stability of analog neural networks, where readout noise is a significant problem (see, for example, [41]).

Much work remains to achieve a full understanding of the interactions between data correlations, readout noise, and ensembling. In this work, we have given a thorough treatment of the convenient special case where features are equicorrelated and readouts do not overlap. Future work should analyze ensembling for codes with an arbitrary correlation structure, in which readouts access randomly chosen, potentially overlapping subsets of features. This will require to average our expressions for the generalization error over randomly drawn masks $\left\{A_{r}\right\}$. This problem has been thoroughly studied in the case where the entries of $A_{r}$ are i.i.d Gaussian [30], as in the ever-popular random feature model. Recent progress on the problem of non-Gaussian projections for a single readout has been made in [42].

## Figures and Tables

**FIG. 5.**
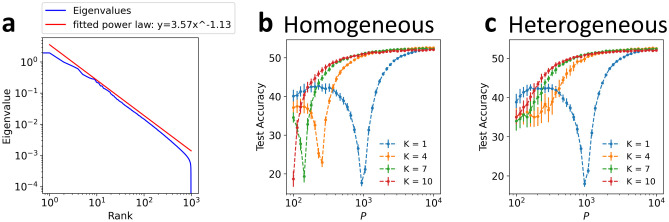
Homogeneous and Heterogeneous Subsampling applied to CIFAR10 demonstrates the benefit of heterogeneous ensembling. (a) Eigenvalue spectrum of the empirical feature covariance matrix $\left.E_{x}\left[\left(\psi (x)-E_{x}\psi (x)\right){\left(\psi (x)-E_{x}\psi (x)\right)}^{⊤}\right\rangle \right]$. Spectrum is well fit by a power law with exponent $\lambda_{n}\propto n^{-\alpha }$ with exponent α=1.13. (b,c) Test Accuracy defined as the fraction of test examples which are incorrectly classified by the ensemble of readouts using homogeneous ensembling strategy (b) and heterogeneous ensembling strategy (c) with $\sigma =\frac{1}{2k}$.

## Appendix A: Application to CIFAR10 Classification Task

In this section, we demonstrate that the qualitative insights gained from our analysis of the linear regression task with Gaussian data carries over to a practical machine learning task. In particular, we show that ensembling with heterogeneous readout connectivity can mitigate double-descent in the CIFAR10 classification task [43] without regularization. The experimental setup is as follows:

To obtain a useful feature map, we first train a deep, fully connected multi-layer perceptron (MLP) to solve the CIFAR10 classification task. The MLP has three hidden layers of size $N_{h}=1000$. We use a training set of 50,000 images $x_{\mu }\in R^{N_{0}},N_{0}=3072$ from $N_{\text{out}\;}=10$ classes. The targets are assigned as one-hot vectors We write this network as follows:

(A1)
$$h=ReLU\left(N_{\text{in }}^{-1/2}W_{\text{in }}x\right)$$

(A2)
$$h_{2}=ReLU\left(M^{-1/2}W_{1}h_{1}\right)$$

(A3)
$$\psi (x)=ReLU\left(M^{-1/2}W_{2}h_{2}\right)$$

(A4)
$$\overset{ˆ}{y}(x)=M^{-1/2}W_{\text{out }}\psi (x)$$

Where $W_{\text{in }}\in R^{N_{h}\times N_{\text{in }}},W_{1}\in R^{N_{h}\times M},W_{2}\in R^{N_{h}\times M},W_{\text{out}\;}\in R^{10\times M}$ We use a MSE loss function:

(A5)
$$\ell \left(\left\{W_{\text{in }},W_{1},W_{2},W_{\text{out}\;}\right\}\right)=\sum_{\mu }\;\left(\overset{ˆ}{y}\left(x_{\mu }\right)-y_{\mu }\right)$$

The predicted class is then assigned as the class corresponding to the component of $\overset{ˆ}{y}$ with maximum value. Training for 2000 steps with full-batch gradient descent and the Adam optimizer at a learning rate of .001, the network achieves a training accuracy of 90% and a test accuracy of ~ %50. The learned features ψ(x) are then extracted and new readout weights are trained using the homogeneous or heterogeneous ensembling strategies with modifications for handling multiple readout classes. An ensemble of k predictions is made:

(A6)
$$y^{r}(x)=\frac{1}{\sqrt{N_{r}}}W_{r}A_{r}\psi (x)$$

for r=1,…,k. Here, each $A_{r}\in R^{N_{r}\times N_{in}}$ implements subsampling of a randomly drawn subset of $N_{r}$ features, and $w_{r}\in R^{N_{\text{out}\;}\times N_{r}}$ predict the class of the input from these subsampled features. The weights $W_{r}$ are trained independently using a pseudoinverse rule, which is equivalent to ridge regression in the limit of zero regularization. Finally, the predictions of the ensemble of readouts are combined using a mean with a nonlinear threshold:

(A7)
$$\overset{ˆ}{y}(x)\;=\sum_{r=1}^{k}\;\;\phi \left(y^{r}(x)\right)$$

(A8)
$$\phi (x)\;=\frac{1}{2}\left(1+tanh\left(5\left(x-\frac{1}{2}\right)\right)\right)$$

In supplemental figure 1, we demonstrate the performance of re-learning ensembles of readout weights using the homogeneous and heterogeneous ensembling strategies. To review, in homogeneous ensembling, the subsampling fractions $\nu_{rr}=\frac{N_{r}}{M}$ are chosen as $\nu_{rr}=1/k,r=1,\ldots ,k$. However, in heterogeneous subsampling, the weights are drawn from a gamma distribution with mean 1/k and variance $\sigma^{2}$, then re-scaled so that $\sum_{r}\;\nu_{rr}=1$. In figure S1, we use σ=1/(2k).

We that the heterogeneous ensembling approach leads to a smooth learning curve without double descent. This effect is most pronounced in the plots of "test accuracy", which is the probability of incorrect classification of a test example. While homogeneous ensembling shifts the double-descent peak toward smaller P, heterogeneous ensembling eliminated the peak. Because the data is heavily correlated, there is no cost to the prediction performance at large P.

## Appendix B: Generalization error of ensembled linear regression from the replica trick

Here we use the Replica Trick from statistical physics to derive analytical expressions for $E_{rr^{\prime }}$. We treat the cases where $r=r^{\prime }$ and $r\neq r^{\prime }$ separately. Following a statistical mechanics approach, we calculate the average generalization error over a Gibbs measure with inverse temperature β;

(B1)
$$Z\;=\int \;\prod_{r}\;\;dwexp\left(-\frac{\beta }{2}\sum_{r}\;\;E_{t}^{r}-\frac{M\beta }{2}\sum_{r,r^{\prime }}\;\;J_{rr^{\prime }}E_{rr^{\prime }}\left(w_{r},w_{r^{\prime }}\right)\right)$$

(B2)
$$E_{t}^{r}\;=\sum_{\mu =1}^{P}\;\;{\left(\frac{1}{\sqrt{N_{r}}}w_{r}^{⊤}A_{r}{\overset{-}{\psi }}_{\mu }+\xi_{r}-y_{\mu }\right)}^{2}+\lambda \left|w_{r}^{2}\right|$$

In the limit where β→∞ the gibbs measure will concentrate around the weight vector ${\overset{ˆ}{w}}_{r}$ which minimizes the regularized loss function. The replica trick relies on the following identity:

(B3)
$$\langle log(Z[𝒟])\rangle_{𝒟}=\underset{n\to 0}{lim}\;\frac{1}{n}log\left({\left\langle Z^{n}\right\rangle }_{𝒟}\right)$$

where $\langle \cdot \rangle_{𝒟}$ represents an average over all quenched disorder in the system. In this case, quenched disorder – the disorder which is fixed prior to and throughout training of the weights – consists of the selected training examples along with their feature noise and label noise: $𝒟={\left\{\psi_{\mu },\sigma^{\mu },\epsilon^{\mu }\right\}}_{\mu =1}^{P}$. The calculation proceeds by first computing the average of the replicated partition function assuming n is a positive integer. Then, in a non-rigorous but standard step, we analytically extend the result to n→0.

### 1. Diagonal Terms

We start by calculating $E_{rr}$ for some fixed choice of r. Noting that the diagonal terms of the generalization error $E_{rr}$ only depend on the learned weights $w_{r}$, and the loss function separates over the readouts, we may consider the Gibbs measure over only these weights:

(B4)
$$Z=\int \;dw_{r}exp\left(-\frac{\beta }{2\lambda }E_{r}^{t}-\frac{JM\beta }{2}E_{rr}\left(w_{r}\right)\right)$$

(B5)
$${\left\langle Z^{n}\right\rangle }_{𝒟}=\int \;\prod_{a}\;\;dw_{r}^{a}E_{\left\{\psi_{\mu },\sigma^{\mu },\epsilon^{\mu }\right\}}\;exp\left(-\frac{\beta M}{2\lambda }\sum_{\mu ,a}\;\;\frac{1}{M}{\left[\frac{1}{\sqrt{\nu_{rr}}}w_{r}^{a⊤}A_{r}\left(\psi_{\mu }+\sigma^{\mu }\right)-w^{*⊤}\psi_{\mu }-\sqrt{M}\left(\epsilon^{\mu }-\xi_{r}^{\mu }\right)\right]}^{2}\right.\;\left.-\frac{\beta }{2}\sum_{a}\;\;{\left|w_{r}^{a}\right|}^{2}-\frac{JM\beta }{2}\sum_{a}\;\;E_{rr}\left(w^{a}\right)\right)$$

Next we must perform the averages over quenched disorder. We first integrate over ${\left\{\psi_{\mu },\sigma^{\mu },\xi_{r}^{\mu },\epsilon^{\mu }\right\}}_{\mu =1}^{P}$. Noting that the scalars

$$h_{\mu }^{ra}\equiv \frac{1}{\sqrt{M}}\left[\frac{1}{\sqrt{\nu_{rr}}}w_{r}^{a⊤}A_{r}\left(\psi_{\mu }+\sigma^{\mu }\right)-w^{*⊤}\psi_{\mu }-\sqrt{M}\left(\epsilon^{\mu }-\xi_{r}^{\mu }\right)\right]$$

are Gaussian random variables (when conditioned on $A_{r}$) with mean zero and covariance:

(B6)
$$\left\langle h_{\mu }^{ra}h_{\nu }^{rb}\right\rangle \;=\delta_{\mu \nu }Q_{ab}^{rr}$$

(B7)
$$Q_{ab}^{rr}\;=\frac{1}{M}\left[\left(\frac{1}{\sqrt{\nu_{rr}}}w_{r}^{a⊤}A_{r}-w^{*⊤}\right)\Sigma_{s}\left(\frac{1}{\sqrt{\nu_{rr}}}A_{r}^{⊤}w_{r}^{b}-w^{*}\right)\right.\;+\left.\frac{1}{\nu_{rr}}w_{r}^{a⊤}A_{r}\Sigma_{0}A_{r}^{⊤}w_{r}^{b}+M\left(\zeta^{2}+\eta_{r}^{2}\right)\right]$$

To perform this integral we re-write in terms of ${\left\{H_{\mu }^{r}\right\}}_{\mu =1}^{P}$, where

(B8)
$$H_{\mu }^{r}=\left[\begin{array}{c} h_{\mu }^{r1}\\ h_{\mu }^{r2}\\ \vdots \\ h_{\mu }^{rn} \end{array}\right]\in R^{n}$$

(B9)
$${\left\langle Z^{n}\right\rangle }_{𝒟}=\int \;\prod_{a}\;\;dw_{r}^{a}E_{\left\{\psi_{\mu },\sigma^{\mu },\epsilon^{\mu }\right\}}exp\left(-\frac{\beta }{2\lambda }\sum_{\mu }\;\;H_{\mu }^{r⊤}H_{\mu }^{r}-\frac{\beta }{2}\sum_{a}\;\;{\left|w_{r}^{a}\right|}^{2}-\frac{JM\beta }{2}\sum_{a}\;\;E_{rr}\left(w^{a}\right)\right)$$

Integrating over the $H_{\mu }^{r}$ we get:

(B10)
$${\left\langle Z^{n}\right\rangle }_{𝒟}=\int \;\prod_{a}\;dw_{r}^{a}exp\left(-\frac{P}{2}logdet\left(I_{n}+\frac{\beta }{\lambda }Q^{rr}\right)-\frac{\beta }{2}\sum_{a}\;\;{\left|w_{r}^{a}\right|}^{2}-\frac{JM\beta }{2}\sum_{a}\;\;E_{rr}\left(w_{r}\right)\right)$$

Next we integrate over $Q^{r}$ and add constraints. We use the following identity:

(B11)
$$1=\prod_{ab^{\prime }}\;\;\int \;dQ_{ab}^{rr}\delta \left(Q_{ab}^{rr}-\frac{1}{M}\left[\left(\frac{1}{\sqrt{\nu_{rr}}}w_{r}^{a⊤}A_{r}-w^{*⊤}\right)\Sigma_{s}\left(\frac{1}{\sqrt{\nu_{rr}}}A_{r}^{⊤}w_{r}^{b}-w^{*}\right)\right.\right.\;\left.\left.+\frac{1}{\nu_{rr}}w_{r}^{a⊤}A_{r}\Sigma_{0}A_{r}^{⊤}w_{r}^{b}+M\left(\zeta^{2}+\eta_{r}^{2}\right)\right]\right)$$

Using the Fourier representation of the delta function, we get:

(B12)
$$1=\prod_{ab}\;\;\int \;\frac{1}{4\pi i/M}dQ_{ab}^{rr}d{\overset{ˆ}{Q}}_{ab}^{rr}exp\left(\frac{M}{2}{\overset{ˆ}{Q}}_{ab}^{rr}\left(Q_{ab}^{rr}-\frac{1}{M}\left[\left(\frac{1}{\sqrt{\nu_{rr}}}w_{r}^{a⊤}A_{r}-w^{*⊤}\right)\Sigma_{s}\left(\frac{1}{\sqrt{\nu_{rr}}}A_{r}^{⊤}w_{r}^{b}-w^{*}\right)\right.\right.\right.\;+\left.\left.\left.\frac{1}{\nu_{rr}}w_{r}^{a⊤}A_{r}\Sigma_{0}A_{r}^{⊤}w_{r}^{b}+M\left(\zeta^{2}+\eta_{r}^{2}\right)\right]\right)\right)$$

Inserting this identity into the replicated partition function and substituting $E_{rr}\left(w_{r}^{a}\right)=Q_{aa}^{rr}-\zeta^{2}$ we find:

(B13)
$${\left\langle Z^{n}\right\rangle }_{𝒟}\propto \;\;\int \;\prod_{ab}\;\;dQ_{ab}^{rr}d{\overset{ˆ}{Q}}_{ab}^{rr}exp\left(-\frac{P}{2}logdet\left(I_{n}+\frac{\beta }{\lambda }Q^{rr}\right)+\frac{1}{2}\sum_{ab}\;\;M{\overset{ˆ}{Q}}_{ab}^{rr}Q_{ab}^{rr}-\frac{JM\beta }{2}\sum_{a}\;\;\left(Q_{aa}^{rr}-\zeta^{2}\right)\right)\;\;\int \;\prod_{a}\;\;dw_{r}^{a}exp\left(-\frac{\beta }{2}\sum_{a}\;\;{\left|w_{r}^{a}\right|}^{2}-\frac{1}{2}\sum_{ab}\;\;{\overset{ˆ}{Q}}_{ab}^{rr}\left[\left(\frac{1}{\sqrt{\nu_{rr}}}w_{r}^{a⊤}A_{r}-w^{*⊤}\right)\Sigma_{s}\left(\frac{1}{\sqrt{\nu_{rr}}}A_{r}^{⊤}w_{r}^{b}-w^{*}\right)\right.\right.\;\left.\left.+\frac{1}{\nu_{rr}}w_{r}^{a⊤}A_{r}\Sigma_{0}A_{r}^{⊤}w_{r}^{b}+M\left(\zeta^{2}+\eta_{r}^{2}\right)\right]\right)$$

In order to perform the Gaussian integral over the $\left\{w_{r}^{a}\right\}$, we unfold over the replica index a. We first define the following:

(B14)
$$\begin{array}{rr} w_{r}^{\cdot } & \;\equiv \left[\begin{array}{c} w_{r}^{1}\\ \vdots \\ w_{r}^{n} \end{array}\right]\\ \; \end{array}$$

(B15)
$$T^{r}\;\equiv \beta I_{n}⊗I_{N_{r}}+{\overset{ˆ}{Q}}^{rr}⊗\left(\frac{1}{\nu_{rr}}A_{r}\left(\Sigma_{s}+\Sigma_{0}\right)A_{r}^{⊤}\right)$$

(B16)
$$V^{r}\;\equiv \left({\overset{ˆ}{Q}}^{rr}⊗I_{N_{r}}\right)\left(1_{n}⊗\frac{1}{\sqrt{\nu_{rr}}}A_{r}\Sigma_{s}w^{*}\right)$$

We then have for the integral over w

(B17)
$$\int \;dw_{r}^{\cdot }exp\left(-\frac{1}{2}w_{r}^{\cdot ⊤}T^{r}w_{r^{\prime }}^{\cdot }+V^{r⊤}w_{r}^{\cdot }\right)$$

(B18)
$$=exp\left(\frac{1}{2}V^{r⊤}{\left(T^{r}\right)}^{-1}V^{r}-\frac{1}{2}logdet\left(T^{r}\right)\right)$$

We can finally write the replicated partition function as:

(B19)
$${\left\langle Z^{n}\right\rangle }_{𝒟}\propto \;\;\int \;\prod_{ab}\;\;dQ_{ab}^{rr}d{\overset{ˆ}{Q}}_{ab}^{r}exp\left(-\frac{P}{2}logdet\left(I_{n}+\frac{\beta }{\lambda }Q^{rr}\right)+\frac{1}{2}\sum_{ab}\;\;M{\overset{ˆ}{Q}}_{ab}^{rr}Q_{ab}^{rr}-\frac{JM\beta }{2}\sum_{a}\;\;\left(Q_{aa}^{rr}-\zeta^{2}\right)\right)\;exp\left(\frac{1}{2}V^{r⊤}{\left(T^{r}\right)}^{-1}V^{r}-\frac{1}{2}logdet\left(T^{r}\right)-\frac{1}{2}\sum_{ab}\;\;{\overset{ˆ}{Q}}_{ab}^{rr}\left(M\left(\zeta^{2}+\eta_{r}^{2}\right)+w^{*⊤}\Sigma_{s}w^{*}\right)\right)$$

We now make the following replica-symmetric ansatz:

(B20)
$$\beta Q_{ab}^{rr}\;=q\delta_{ab}+q_{0}$$

(B21)
$$\beta^{-1}{\overset{ˆ}{Q}}_{ab}^{rr}\;=\overset{ˆ}{q}\delta_{ab}+{\overset{ˆ}{q}}_{0}$$

which is well-motivated because the loss function is convex. We may then rewrite the partition function as follows:

(B22)
$${\left\langle Z^{n}\right\rangle }_{𝒟}=exp\left(-\frac{nM}{2}g\left[q,q_{0},\overset{ˆ}{q},{\overset{ˆ}{q}}_{0}\right]\right)$$

Where the effective action is written:

(B23)
$$g\left[q,q_{0},\overset{ˆ}{q},{\overset{ˆ}{q}}_{0}\right]=\alpha \left[log\left(1+\frac{q}{\lambda }\right)+\frac{q_{0}}{\lambda +q}\right]-\left(q\overset{ˆ}{q}+q{\overset{ˆ}{q}}_{0}+q_{0}\overset{ˆ}{q}\right)+J\left[\left(q+q_{0}\right)-\beta \zeta^{2}\right]\;\;-\frac{\beta }{\nu_{rr}M}{\overset{ˆ}{q}}^{2}w^{*⊤}\Sigma_{s}A_{r}^{⊤}G^{-1}A_{r}\Sigma_{s}w^{*}+\frac{1}{M}\left[logdet(G)+{\overset{ˆ}{q}}_{0}tr\left[G^{-1}\tilde{\Sigma }\right]\right]+\beta \overset{ˆ}{q}\left(\zeta^{2}+\eta_{r}^{2}+\frac{1}{M}w^{*⊤}\Sigma_{s}w^{*}\right)$$

Where $G\equiv I_{N_{r}}+\overset{ˆ}{q}\tilde{\Sigma }$ and $\tilde{\Sigma }\equiv \frac{1}{\nu_{rr}}A_{r}\left(\Sigma_{s}+\Sigma_{0}\right)A_{r}^{⊤}$

We have

(B24)
$$E_{rr}=\frac{\partial }{\partial J}\underset{\beta \to \infty }{lim}\;\frac{1}{\beta }g\left[q,q_{0},\overset{ˆ}{q},{\overset{ˆ}{q}}_{0}\right]$$

Where g is evaluated at the values of $q,q_{0},\overset{ˆ}{q},{\overset{ˆ}{q}}_{0}$ which minimize g, in accordance with the method of steepest descent.

(B25)
$$E_{rr}\;=\partial_{J}\left(\frac{1}{M}w^{⋆⊤}\left[\overset{ˆ}{q}\Sigma_{s}-\frac{1}{\nu_{rr}}{\overset{ˆ}{q}}^{2}\Sigma_{s}A_{r}^{⊤}G^{-1}A_{r}\Sigma_{s}\right]w^{*}+\overset{ˆ}{q}\left(\zeta^{2}+\eta_{r}^{2}\right)-J\zeta^{2}\right)\;\;=\left[\partial_{J}\overset{ˆ}{q}\right]\left(\frac{1}{M}w^{⋆⊤}\left[\Sigma_{s}-\frac{2}{\nu_{rr}}\overset{ˆ}{q}\Sigma_{s}A_{r}^{⊤}G^{-1}A_{r}\Sigma_{s}+\frac{1}{\nu_{rr}}{\overset{ˆ}{q}}^{2}\Sigma_{s}A_{r}^{⊤}G^{-1}\tilde{\Sigma }G^{-1}A_{r}\Sigma_{s}\right]w^{*}+\zeta^{2}+\eta_{r}^{2}\right)-\zeta^{2}$$

To complete the calculation, we need to find $\partial_{J}\overset{ˆ}{q}$. We may do this by examining two of the saddle-point equations:

(B26)
$$\frac{\partial g}{\partial q_{0}}=0=\frac{\alpha }{\lambda +q}-\overset{ˆ}{q}+J\;\;\Rightarrow \overset{ˆ}{q}=\frac{\alpha }{\lambda +q}+J$$

(B27)
$$\frac{\partial g}{\partial {\overset{ˆ}{q}}_{0}}=0=-q+\frac{1}{M}tr\left[G^{-1}\tilde{\Sigma }\right]\;\Rightarrow q=\frac{1}{M}tr\left[G^{-1}\tilde{\Sigma }\right]$$

These two equations may in principle be solved for the dominant values of q and $\overset{ˆ}{q}$. Letting κ=λ+q, we get:

(B28)
$$\partial_{J}\overset{ˆ}{q}=-\frac{\alpha }{\kappa^{2}}\partial_{J}q+1$$

(B29)
$$\partial_{J}q=-\frac{1}{M}\partial_{J}\overset{ˆ}{q}tr\left[{\left(G^{-1}\tilde{\Sigma }\right)}^{2}\right]$$

Solving this system of equations, we find $\partial_{J}\overset{ˆ}{q}=\frac{1}{1-\gamma }$ where $\gamma \equiv \frac{\alpha }{M\kappa^{2}}tr\left[{\left(G^{-1}\tilde{\Sigma }\right)}^{2}\right]$

(B30)
$$E_{rr}\;=\frac{1}{1-\gamma }\frac{1}{M}w^{⋆⊤}\left[\Sigma_{s}-\frac{2}{\nu_{rr}}\overset{ˆ}{q}\Sigma_{s}A_{r}^{⊤}G^{-1}A_{r}\Sigma_{s}+\frac{1}{\nu_{rr}}{\overset{ˆ}{q}}^{2}\Sigma_{s}A_{r}^{⊤}G^{-1}\tilde{\Sigma }G^{-1}A_{r}\Sigma_{s}\right]w^{*}+\frac{\gamma \zeta^{2}+\eta_{r}^{2}}{1-\gamma }$$

(B31)
$$=\frac{1}{1-\gamma }\frac{1}{M}w^{⋆⊤}\left[\Sigma_{s}-\frac{1}{\nu_{rr}}\overset{ˆ}{q}\Sigma_{s}A_{r}^{⊤}G^{-1}A_{r}\Sigma_{s}-\frac{1}{\nu_{rr}}\overset{ˆ}{q}\Sigma_{s}A_{r}^{⊤}G^{-2}A_{r}\Sigma_{s}\right]w^{*}+\frac{\gamma \zeta^{2}+\eta_{r}^{2}}{1-\gamma }$$

### 2. Off-Diagonal Terms

We now calculate $E_{rr^{\prime }}$ for $r\neq r^{\prime }$. We now must consider the joint Gibbs Measure over $w_{r}$ and $w_{r^{\prime }}$ :

(B32)
$$Z=\int \;dw_{r}dw_{r^{\prime }}exp\left(-\frac{\beta }{2\lambda }\left(E_{r}^{t}+E_{r^{\prime }}^{t}\right)-\frac{JM\beta }{2}E_{rr^{\prime }}\left(w_{r},w_{r^{\prime }}\right)\right)$$

(B33)
$${\left\langle Z^{n}\right\rangle }_{𝒟}=\int \;\prod_{a}\;\;dw_{r}^{a}dw_{r^{\prime }}^{a}E_{\left\{\psi_{\mu },\sigma^{\mu },\epsilon^{\mu }\right\}}$$

(B34)
$$exp\left(-\frac{\beta M}{2\lambda }\sum_{\mu ,a}\;\;\frac{1}{M}\left[{\left(h_{\mu }^{ra}\right)}^{2}+{\left(h_{\mu }^{r^{\prime }a}\right)}^{2}\right]-\frac{\beta }{2}\sum_{a}\;\;\left[{\left|w_{r}^{a}\right|}^{2}+{\left|w_{r^{\prime }}^{a}\right|}^{2}\right]-\frac{JM\beta }{2}\sum_{a}\;\;E_{rr^{\prime }}\left(w_{r}^{a},w_{r^{\prime }}^{a}\right)\right)$$

Where the $h_{\mu }^{ra}$ are defined as before. Next we must perform the averages over quenched disorder. We first integrate over $\left\{\psi_{\mu },\epsilon_{\mu }\right\}$. To do so, we note that the $h_{\mu }^{ra}$ are Gaussian random variables with covariance structure:

(B35)
$$\left\langle h_{\mu }^{ra}h_{\nu }^{r^{\prime }b}\right\rangle \;=\delta_{\mu \nu }Q_{ab}^{rr^{\prime }}$$

(B36)
$$Q_{ab}^{rr^{\prime }}\;=\frac{1}{M}\left[\left(\frac{1}{\sqrt{\nu_{rr}}}w_{r}^{a⊤}A_{r}-w^{*⊤}\right)\Sigma_{s}\left(\frac{1}{\sqrt{\nu_{r^{\prime }r^{\prime }}}}A_{r^{\prime }}^{⊤}w_{r^{\prime }}^{b}-w^{*}\right)\right.\;+\left.\frac{1}{\sqrt{\nu_{rr}\nu_{r^{\prime }r^{\prime }}}}w_{r}^{a⊤}A_{r}\Sigma_{0}A_{r^{\prime }}^{⊤}w_{r^{\prime }}^{b}+M\zeta^{2}\right]$$

To perform this integral we re-write in terms of ${\left\{H_{\mu }\right\}}_{\mu =1}^{P}$, where

(B37)
$$H_{\mu }=\left[\begin{array}{c} H_{\mu }^{r}\\ H_{\mu }^{r^{\prime }} \end{array}\right]\in R^{2n}$$

(B38)
$${\left\langle Z^{n}\right\rangle }_{𝒟}=\int \;\prod_{a}\;\;dw_{r}^{a}dw_{r^{\prime }}^{a}E_{\left\{\psi_{\mu },\sigma^{\mu },\epsilon^{\mu }\right\}}\;exp\left(-\frac{\beta }{2\lambda }\sum_{\mu }\;\;H_{\mu }^{⊤}H_{\mu }-\frac{\beta }{2}\sum_{a}\;\;\left[{\left|w_{r}^{a}\right|}^{2}+{\left|w_{r^{\prime }}^{a}\right|}^{2}\right]-\frac{JM\beta }{2}\sum_{a}\;\;E_{rr^{\prime }}\left(w_{r}^{a},w_{r^{\prime }}^{a}\right)\right)$$

Integrating over $H_{\mu }$ we get:

(B39)
$${\left\langle Z^{n}\right\rangle }_{𝒟}=\int \;\prod_{a}\;\;dw_{r}^{a}dw_{r^{\prime }}^{a}\;exp\left(-\frac{P}{2}logdet\left(I_{2n}+\frac{\beta }{\lambda }Q\right)-\frac{\beta }{2}\sum_{a}\;\;\left[{\left|w_{r}^{a}\right|}^{2}+{\left|w_{r^{\prime }}^{a}\right|}^{2}\right]-\frac{JM\beta }{2}\sum_{a}\;\;E_{rr}\left(w_{r}^{a},w_{r^{\prime }}^{a}\right)\right)$$

Where we have defined the matrix Q so that:

(B40)
$$Q=\left[\begin{array}{ll} Q^{rr} & Q^{rr^{\prime }}\\ Q^{rr^{\prime }} & Q^{r^{\prime }r^{\prime }} \end{array}\right]$$

Next we integrate over Q and add constraints. We use the following identity:

(B41)
$$1=\prod_{ab}\;\;\int \;dQ_{ab}^{rr^{\prime }}\delta \left(Q_{ab}^{rr^{\prime }}-\frac{1}{M}\left[\left(\frac{1}{\sqrt{\nu_{rr}}}w_{r}^{a⊤}A_{r}-w^{*⊤}\right)\Sigma_{s}\left(\frac{1}{\sqrt{\nu_{r^{\prime }r^{\prime }}}}A_{r^{\prime }}^{⊤}w_{r^{\prime }}^{b}-w^{*}\right)\right.\right.\;\left.\left.+\frac{1}{\sqrt{\nu_{rr}\nu_{r^{\prime }r^{\prime }}}}w_{r}^{a⊤}A_{r}\Sigma_{0}A_{r^{\prime }}^{⊤}w_{r^{\prime }}^{b}+M\zeta^{2}\right]\right)$$

Using the Fourier representation of the delta function, we get:

(B42)
$$1=\prod_{ab}\;\;\int \;\frac{1}{4\pi i/M}dQ_{ab}^{rr^{\prime }}d{\overset{ˆ}{Q}}_{ab}^{rr^{\prime }}exp\left(\frac{M}{2}{\overset{ˆ}{Q}}_{ab}^{rr^{\prime }}\left(Q_{ab}^{rr^{\prime }}-\frac{1}{M}\left[\left(\frac{1}{\sqrt{\nu_{rr}}}w_{r}^{a⊤}A_{r}-w^{*⊤}\right)\Sigma_{s}\left(\frac{1}{\sqrt{\nu_{r^{\prime }r^{\prime }}}}A_{r^{\prime }}^{⊤}w_{r^{\prime }}^{b}-w^{*}\right)\right.\right.\right.\;\left.\left.\left.+\frac{1}{\nu_{rr}}w_{r}^{a⊤}A_{r}\Sigma_{0}A_{r^{\prime }}^{⊤}w_{r^{\prime }}^{b}+M\zeta^{2}\right]\right)\right)$$

Inserting this identity and the corresponding statements for $Q_{ab}^{rr}$ and $Q_{ab}^{r^{\prime }r^{\prime }}$ into the replicated partition function and substituting $E_{rr^{\prime }}\left(w^{a}\right)=Q_{aa}^{rr^{\prime }}-\zeta^{2}$ we find:

(B43)
$${\langle Z^{n}\rangle }_{𝒟}\propto \int \prod_{abr_{1}r_{2}}dQ_{ab}^{r_{1}r_{2}}d{\hat{Q}}_{ab}^{r_{1}r_{2}}\;\exp\left(-\frac{P}{2}\log\;\det\left(I_{2n}+\frac{\beta }{\lambda }Q\right)+\frac{1}{2}\sum_{abr_{1}r_{2}}M{\hat{Q}}_{ab}^{r_{1}r_{2}}Q_{ab}^{r_{1}r_{2}}-\frac{JM\beta }{2}\sum_{a}\left(Q_{aa}^{rr^{\prime }}-\zeta^{2}\right)\right)\;\int \prod_{a}dw_{r}^{a}dw_{r^{\prime }}^{a}\exp(-\frac{\beta }{2}\sum_{a}\left[{\left|w_{r}^{a}\right|}^{2}+{\left|w_{r^{\prime }}^{a}\right|}^{2}\right]-\frac{1}{2}\sum_{abr_{1}r_{2}}{\hat{Q}}_{ab}^{r_{1}r_{2}}[\left(\frac{1}{\sqrt{\nu_{r_{1}}}}w_{r_{1}}^{a⊤}A_{r_{1}}-w^{*⊤}\right)\Sigma_{s}\left(\frac{1}{\sqrt{\nu_{r_{2}}}}A_{r_{2}}^{⊤}w_{r_{2}}^{b}-w^{*}\right)\;+\frac{1}{\sqrt{\nu_{r_{1}}\nu_{r_{2}}}}w_{r_{1}}^{a⊤}A_{r_{1}}\Sigma_{0}A_{r_{2}}^{⊤}w_{r_{2}}^{b}+M\zeta^{2}])$$

Where sums over $r_{1}$ and $r_{2}$ run over $\left\{r,r^{\prime }\right\}$.

In order to perform the Gaussian integral over the $\left\{w_{r}^{a}\right\}$, we unfold in two steps. We first define the following:

(B44)
$$\begin{array}{rr} w_{r}^{\cdot } & \;\equiv \left[\begin{array}{c} w_{r}^{1}\\ \vdots \\ w_{r}^{n} \end{array}\right] \end{array}$$

(B45)
$${\left[{\overset{ˆ}{Q}}^{rr^{\prime }}\right]}_{ab}\;\equiv {\overset{ˆ}{Q}}_{ab}^{rr^{\prime }}$$

(B46)
$${\tilde{\Sigma }}_{rr^{\prime }}\;\equiv \frac{1}{\sqrt{\nu_{rr}\nu_{r^{\prime }r^{\prime }}}}A_{r}\left[\Sigma_{s}+\Sigma_{0}\right]A_{r^{\prime }}^{⊤}$$

(B47)
$$T^{rr^{\prime }}\;\equiv \beta \delta_{rr^{\prime }}I_{n}⊗I_{N_{r}}+{\overset{ˆ}{Q}}^{rr^{\prime }}⊗{\tilde{\Sigma }}_{rr^{\prime }}$$

Unfolding over the replica indices, we then get:

(B48)
$${\left\langle Z^{n}\right\rangle }_{𝒟}\propto \int \;\prod_{abr_{1}r_{2}}\;\;dQ_{ab}^{r_{1}r_{2}}d{\overset{ˆ}{Q}}_{ab}^{r_{1}r_{2}}\;exp\left(-\frac{P}{2}logdet\left(I_{2n}+\frac{\beta }{\lambda }Q\right)+\frac{1}{2}\sum_{abr_{1}r_{2}}\;\;M{\overset{ˆ}{Q}}_{ab}^{r_{1}r_{2}}Q_{ab}^{r_{1}r_{2}}-\frac{JM\beta }{2}\sum_{a}\;\;\left(Q_{aa}^{rr^{\prime }}-\zeta^{2}\right)\right)\;exp\left(-\frac{1}{2}\sum_{abr_{1}r_{2}}\;\;{\overset{ˆ}{Q}}_{ab}^{r_{1}r_{2}}\left(w^{*⊤}\Sigma_{s}w^{*}+M\zeta^{2}\right)\right)\;\;\int \;dw_{r}^{\cdot }dw_{r^{\prime }}^{\cdot }exp\left(-\frac{1}{2}\sum_{r_{1}r_{2}}\;\;w_{r_{1}}^{\cdot ⊤}T^{r_{1}r_{2}}w_{r_{2}}+\sum_{r_{1}r_{2}}\;\;{\left[\left({\overset{ˆ}{Q}}^{r_{1}r_{2}}⊗I_{N_{r_{1}}}\right)\left(1_{n}⊗\frac{1}{\sqrt{\nu_{r_{1}}}}A_{r_{1}}\Sigma_{s}w^{*}\right)\right]}^{⊤}w_{r_{1}}\right)$$

Note that the dimensionality of $T^{r_{1}r_{2}}$ varies for different choices of $r_{1}$ and $r_{2}$. Next, we unfold over the two readouts:

(B49)
$$\begin{array}{rr} w & \;\equiv \left[\begin{array}{c} w_{r}^{\cdot }\\ w_{r^{\prime }} \end{array}\right] \end{array}$$

(B50)
$$\begin{array}{rr} T & \;\equiv \left[\begin{array}{cc} T^{rr} & T^{rr^{\prime }}\\ T^{r^{\prime }r} & T^{r^{\prime }r^{\prime }} \end{array}\right] \end{array}$$

(B51)
$$\begin{array}{rr} V & \;\equiv \left[\begin{array}{c} \left(\left({\overset{ˆ}{Q}}^{rr}+{\overset{ˆ}{Q}}^{rr^{\prime }}\right)⊗I_{N_{r}}\right)\left(1_{n}⊗\frac{1}{\sqrt{\nu_{rr}}}A_{r}\Sigma_{s}w^{*}\right)\\ \left.\left(\left({\overset{ˆ}{Q}}^{r^{\prime }r^{\prime }}+{\overset{ˆ}{Q}}^{r^{\prime }r}\right)⊗I_{N_{r^{\prime }}}\right)\left(1_{n}⊗\frac{1}{\sqrt{\nu_{r^{\prime }r^{\prime }}}}A_{r^{\prime }}\Sigma_{s}w^{*}\right)\right] \end{array}\right] \end{array}$$

The integral over w then becomes:

(B52)
$$\int \;dwexp\left(-\frac{1}{2}w^{⊤}Tw+V^{⊤}w\right)\propto exp\left(\frac{1}{2}V^{⊤}T^{-1}V-\frac{1}{2}logdetT\right)$$

We are now ready to make a replica-symmetric ansatz. The order parameter that we wish to constrain is $Q_{ab}^{rr^{\prime }}$. Overlaps go between the weights from different replicas of the system as well as different readouts. The scale of the overlap between two measurements depends on their overlap with each other and with the principal components of the data distribution. An ansatz which is replica-symmetric but makes no assumptions about the overlaps between different measurements is as follows:

(B53)
$$\beta Q_{ab}^{r_{1}r_{2}}\;=Q^{r_{1}r_{2}}\delta_{ab}+q^{r_{1}r_{2}}$$

(B54)
$$\beta^{-1}{\overset{ˆ}{Q}}_{ab}^{r_{1}r_{2}}\;={\overset{ˆ}{Q}}^{r_{1}r_{2}}\delta_{ab}+{\overset{ˆ}{q}}^{r_{1}r_{2}}$$

Next step is to plug the RS ansatz into the free energy and simplify. To make calculations more transparent, we re-label the paramters in the RS ansatz as follows:

(B55)
$$\beta Q^{rr}\;=qI+Q11^{⊤}$$

(B56)
$$\beta Q^{r^{\prime }r^{\prime }}\;=rI+R11^{⊤}$$

(B57)
$$\beta Q^{rr^{\prime }}\;=cI+C11^{⊤}$$

(B58)
$$\beta^{-1}{\overset{ˆ}{Q}}^{rr}\;=\overset{ˆ}{q}I+\overset{ˆ}{Q}11^{⊤}$$

(B59)
$$\beta^{-1}{\overset{ˆ}{Q}}^{r^{\prime }r^{\prime }}\;=\overset{ˆ}{r}I+\overset{ˆ}{R}11^{⊤}$$

(B60)
$$\beta^{-1}{\overset{ˆ}{Q}}^{rr^{\prime }}\;=\overset{ˆ}{c}I+\overset{ˆ}{C}11^{⊤}$$

In order to simplify $logdet\left(\lambda I_{2n}+\beta Q\right)$, we note that this is a symmetric 2-by-2-block matrix, where each block commutes with all other blocks. We may then use [44]'s result to simplify.

(B61)
$$\text{log}\text{det}\left(\lambda I_{2n}+\beta Q\right)=n\left[\text{log}\left(\left(\lambda +q\right)\left(\lambda +r\right)-c^{2}\right)+\frac{\left(\lambda +q\right)R+\left(\lambda +r\right)Q-2cC}{\left(\lambda +q\right)\left(\lambda +r\right)-c^{2}}\right]+𝒪\left(n^{2}\right)$$

(B62)
$$\sum_{abr_{1}r_{2}}\;\;{\overset{ˆ}{Q}}_{ab}^{r_{1}r_{2}}Q_{ab}^{r_{1}r_{2}}=n[(q\overset{ˆ}{q}+\overset{ˆ}{q}Q+q\overset{ˆ}{Q})+(r\overset{ˆ}{r}+\overset{ˆ}{r}R+r\overset{ˆ}{R})+2(c\overset{ˆ}{c}+\overset{ˆ}{c}C+c\overset{ˆ}{C})]+𝒪\left(n^{2}\right)$$

(B63)
$$\sum_{a}\;\;\left(Q_{aa}^{rr^{\prime }}-\zeta^{2}\right)=n\left[\frac{1}{\beta }(c+C)-\zeta^{2}\right]+𝒪\left(n^{2}\right)$$

(B64)
$$\sum_{abr_{1}r_{2}}\;\;{\overset{ˆ}{Q}}_{ab}^{r_{1}r_{2}}=\beta n[\overset{ˆ}{q}+\overset{ˆ}{r}+2\overset{ˆ}{c}]$$

(B65)
$$\text{log}\text{det}\left(T\right)=n\left[\text{log}\left(\beta \right)+\text{log}\text{det}\left[\begin{array}{cc} G_{rr} & G_{rr^{\prime }}\\ G_{r^{\prime }r} & G_{r^{\prime }r^{\prime }} \end{array}\right]\right.+\overset{ˆ}{q}\;tr\left(D_{rr}{\tilde{\Sigma }}_{rr}+\overset{ˆ}{c\;}\text{tr}\left(D_{rr^{\prime }}{\tilde{\Sigma }}_{r^{\prime }r}\right)+\overset{ˆ}{r}\;tr\left(D_{r^{\prime }r^{\prime }}{\tilde{\Sigma }}_{r^{\prime }r^{\prime }}\right)+\overset{ˆ}{c\;}tr\left(D_{r^{\prime }r}{\tilde{\Sigma }}_{rr^{\prime }}\right)\right]+𝒪\left(n^{2}\right)$$

(B66)
$$\text{where }G_{rr}=I_{N_{r}}+\overset{ˆ}{q}{\tilde{\Sigma }}_{rr}\;G_{r^{\prime }r^{\prime }}=I_{N_{r^{\prime }}}+\overset{ˆ}{r}{\tilde{\Sigma }}_{r^{\prime }r^{\prime }}\;G_{rr^{\prime }}=\overset{ˆ}{c}{\tilde{\Sigma }}_{rr^{\prime }}\;G_{r^{\prime }r}=\overset{ˆ}{c}{\tilde{\Sigma }}_{r^{\prime }r}$$

and where the $D_{r_{1}r_{2}}$ matrices are defined implicitly through the following equation:

(B67)
$${\left[\begin{array}{cc} G_{rr} & G_{rr^{\prime }}\\ G_{r^{\prime }r} & G_{r^{\prime }r^{\prime }} \end{array}\right]}^{-1}=\left[\begin{array}{cc} D_{rr} & D_{rr^{\prime }}\\ D_{r^{\prime }r} & D_{r^{\prime }r^{\prime }} \end{array}\right]$$

The $D_{r_{1}r_{2}}$ matrices can be expressed in terms of the $G_{r_{1}r_{2}}$ and their inverses by the standard 2 × 2 block matrix inversion formula (see, for example, [45]). Applying this formula gives the following:

(B68)
$$D_{rr}={\left[I+\overset{ˆ}{q}{\tilde{\Sigma }}_{rr}-{\overset{ˆ}{c}}^{2}{\tilde{\Sigma }}_{rr^{\prime }}{\left(I+\overset{ˆ}{r}{\tilde{\Sigma }}_{r^{\prime }r^{\prime }}\right)}^{-1}{\tilde{\Sigma }}_{r^{\prime }r}\right]}^{-1}$$

(B69)
$$D_{r^{\prime }r^{\prime }}={\left[I+\overset{ˆ}{r}{\tilde{\Sigma }}_{r^{\prime }r^{\prime }}-{\overset{ˆ}{c}}^{2}{\tilde{\Sigma }}_{r^{\prime }r}{\left(I+\overset{ˆ}{q}{\tilde{\Sigma }}_{rr}\right)}^{-1}{\tilde{\Sigma }}_{rr^{\prime }}\right]}^{-1}$$

(B70)
$$D_{rr^{\prime }}=-\overset{ˆ}{c}D_{rr}{\tilde{\Sigma }}_{rr^{\prime }}G_{r^{\prime }r^{\prime }}^{-1}$$

(B71)
$$D_{r^{\prime }r}=-\overset{ˆ}{c}D_{r^{\prime }r^{\prime }}{\tilde{\Sigma }}_{r^{\prime }r}G_{rr}^{-1}$$

(B72)
$$V^{⊤}T^{-1}V=n\beta w^{*⊤}{\left[\begin{array}{c} \frac{1}{\sqrt{\nu_{rr}}}(\overset{ˆ}{q}+\overset{ˆ}{c})A_{r}\Sigma_{s}\\ \frac{1}{\sqrt{\nu_{r^{\prime }r^{\prime }}}}(\overset{ˆ}{r}+\overset{ˆ}{c})A_{r^{\prime }}\Sigma_{s} \end{array}\right]}^{⊤}{\left[\begin{array}{ll} G_{rr} & G_{rr^{\prime }}\\ G_{r^{\prime }r} & G_{r^{\prime }r^{\prime }} \end{array}\right]}^{-1}\left[\begin{array}{c} \frac{1}{\sqrt{\nu_{rr}}}(\overset{ˆ}{q}+\overset{ˆ}{c})A_{r}\Sigma_{s}\\ \frac{1}{\sqrt{\nu_{r^{\prime }r^{\prime }}}}(\overset{ˆ}{r}+\overset{ˆ}{c})A_{r^{\prime }}\Sigma_{s} \end{array}\right]w^{*}+𝒪\left(n^{2}\right)$$

Collecting these terms, we may write the replicated partition function as follows:

(B73)
$${\left\langle Z^{n}\right\rangle }_{𝒟}=exp\left(-\frac{nM}{2}g[q,Q,r,R,c,C,\overset{ˆ}{q},\overset{ˆ}{Q},\overset{ˆ}{r},\overset{ˆ}{R},\overset{ˆ}{c},\overset{ˆ}{C}]\right)$$

Where the effective action is written:

(B74)
$$g[q,Q,r,R,c,C,\overset{ˆ}{q},\overset{ˆ}{Q},\overset{ˆ}{r},\overset{ˆ}{R},\overset{ˆ}{c},\overset{ˆ}{C}]=$$

(B75)
$$\alpha \left[log\left((\lambda +q)(\lambda +r)-c^{2}\right)+\frac{(\lambda +q)R+(\lambda +r)Q-2Gg}{(\lambda +q)(\lambda +r)-c^{2}}\right]$$

(B76)
$$-[(q\overset{ˆ}{q}+\overset{ˆ}{q}Q+q\overset{ˆ}{Q})+(r\overset{ˆ}{r}+\overset{ˆ}{r}R+r\overset{ˆ}{R})+2(c\overset{ˆ}{c}+\overset{ˆ}{c}C+c\overset{ˆ}{C})]$$

(B77)
$$+J(c+C)-\beta J\zeta^{2}$$

(B78)
$$+\beta [\overset{ˆ}{q}+\overset{ˆ}{r}+2\overset{ˆ}{c}]\left(\frac{1}{M}w^{*⊤}\Sigma w^{*}+\zeta^{2}\right)$$

(B79)
$$-\beta w^{\text{*}⊤}{\left[\frac{\frac{1}{\sqrt{\nu_{rr}}}\left(\hat{q}+\hat{c}\right)A_{r}\Sigma_{s}}{\frac{1}{\sqrt{\nu_{r^{\prime }r^{\prime }}}}\left(\hat{r}+\hat{c}\right)A_{r^{\prime }}\Sigma_{s}}\right]}^{⊤}G^{-1}\left[\frac{\frac{1}{\sqrt{\nu_{rr}}}\left(\hat{q}+\hat{c}\right)A_{r}\Sigma_{s}}{\frac{1}{\sqrt{\nu_{r^{\prime }r^{\prime }}}}\left(\hat{r}+\hat{c}\right)A_{r^{\prime }}\Sigma_{s}}\right]w^{\text{*}}$$

(B80)
$$+\frac{1}{M}\left[\text{log}\left(\beta \right)+\text{log}\text{det}\left(G\right)+\overset{ˆ}{Q\;}\text{tr}\left(D_{rr}{\tilde{\Sigma }}_{rr}\right)+\overset{ˆ}{C\;}\text{tr}\left(D_{rr^{\prime }}{\tilde{\Sigma }}_{r^{\prime }r}\right)+\overset{ˆ}{R}\;tr\left(D_{r^{\prime }r^{\prime }}{\tilde{\Sigma }}_{r^{\prime }r^{\prime }}\right)+\overset{ˆ}{C\;}tr\left(D_{r^{\prime }r}{\tilde{\Sigma }}_{rr^{\prime }}\right)\right]$$

where we have defined $G\equiv \left[\begin{array}{ll} G_{rr} & G_{rr^{\prime }}\\ G_{r^{\prime }r} & G_{r^{\prime }r^{\prime }} \end{array}\right]$

We have:

(B81)
$$E_{rr^{\prime }}=\frac{\partial }{\partial J}\underset{\beta \to \infty }{lim}\;\frac{1}{\beta }g[q,Q,r,R,c,C,\overset{ˆ}{q},\overset{ˆ}{Q},\overset{ˆ}{r},\overset{ˆ}{R},\overset{ˆ}{c},\overset{ˆ}{C}]$$

Where g is evaluated at the values of $Q,q,R,r,C,c,\overset{ˆ}{Q},\overset{ˆ}{q},\overset{ˆ}{R},\overset{ˆ}{r},\overset{ˆ}{C},\overset{ˆ}{c}$ which minimize g, in accordance with the method of steepest descent (and thus implicitly depend on J). This gives:

(B82)
$$E_{rr^{\prime }}=-\zeta^{2}+\left[\frac{\partial \overset{ˆ}{q}}{\partial J}+\frac{\partial \overset{ˆ}{r}}{\partial J}+2\frac{\partial \overset{ˆ}{c}}{\partial J}\right]\left(\frac{1}{M}w^{*⊤}\Sigma_{s}w^{*}+\zeta^{2}\right)$$

(B83)
$$-\frac{2}{M}w^{*⊤}{\left[\begin{array}{c} \frac{1}{\sqrt{\nu_{rr}}}\left(\frac{\partial \overset{ˆ}{q}}{\partial J}+\frac{\partial \overset{ˆ}{c}}{\partial J}\right)A_{r}\Sigma_{s}\\ \frac{1}{\sqrt{\nu_{r^{\prime }r^{\prime }}}}\left(\frac{\partial \overset{ˆ}{r}}{\partial J}+\frac{\partial \overset{ˆ}{c}}{\partial J}\right)A_{r^{\prime }}\Sigma_{s} \end{array}\right]}^{⊤}G^{-1}\left[\begin{array}{c} \frac{1}{\sqrt{\nu_{rr}}}(\overset{ˆ}{q}+\overset{ˆ}{c})A_{r}\Sigma_{s}\\ \frac{1}{\sqrt{\nu_{r^{\prime }r^{\prime }}}}(\overset{ˆ}{r}+\overset{ˆ}{c})A_{r^{\prime }}\Sigma_{s} \end{array}\right]w^{*}$$

(B84)
$$+\frac{1}{M}w^{*⊤}{\left[\begin{array}{c} \frac{1}{\sqrt{\nu_{rr}}}(\overset{ˆ}{q}+\overset{ˆ}{c})A_{r}\Sigma_{s}\\ \frac{1}{\sqrt{\nu_{r^{\prime }r^{\prime }}}}(\overset{ˆ}{r}+\overset{ˆ}{c})A_{r^{\prime }}\Sigma_{s} \end{array}\right]}^{⊤}G^{-1}\frac{\partial G}{\partial J}G^{-1}\left[\begin{array}{c} \frac{1}{\sqrt{\nu_{rr}}}(\overset{ˆ}{q}+\overset{ˆ}{c})A_{r}\Sigma_{s}\\ \frac{1}{\sqrt{\nu_{r^{\prime }r^{\prime }}}}(\overset{ˆ}{r}+\overset{ˆ}{c})A_{r^{\prime }}\Sigma_{s} \end{array}\right]w^{*}$$

(B85)
$$\frac{\partial G}{\partial J}=\frac{\partial G}{\partial \overset{ˆ}{q}}\frac{\partial \overset{ˆ}{q}}{\partial J}+\frac{\partial G}{\partial \overset{ˆ}{r}}\frac{\partial \overset{ˆ}{r}}{\partial J}+\frac{\partial G}{\partial \overset{ˆ}{c}}\frac{\partial \overset{ˆ}{c}}{\partial J}=\left[\begin{array}{ll} \frac{\partial \overset{ˆ}{q}}{\partial J}{\tilde{\Sigma }}_{rr} & \frac{\partial \overset{ˆ}{c}}{\partial J}{\tilde{\Sigma }}_{rr^{\prime }}\\ \frac{\partial \overset{ˆ}{c}}{\partial J}{\tilde{\Sigma }}_{r^{\prime }r} & \frac{\partial \overset{ˆ}{r}}{\partial J}{\tilde{\Sigma }}_{r^{\prime }r^{\prime }} \end{array}\right]$$

All that remains is to calculate the saddle-point values of $\overset{ˆ}{q},\overset{ˆ}{r},\overset{ˆ}{c}$ and their derivatives with respect to J at J=0.

(B86)
$$\frac{\partial g}{\partial Q}=0=\frac{\alpha (\lambda +r)}{(\lambda +q)(\lambda +r)-C^{2}}-\overset{ˆ}{q}$$

(B87)
$$\frac{\partial g}{\partial \overset{ˆ}{Q}}=0=-q+\frac{1}{M}tr\left(D_{rr}{\tilde{\Sigma }}_{rr}\right)$$

(B88)
$$\frac{\partial g}{\partial R}=0=\frac{\alpha (\lambda +q)}{(\lambda +q)(\lambda +r)-C^{2}}-\overset{ˆ}{r}$$

(B89)
$$\frac{\partial g}{\partial \overset{ˆ}{R}}=0=-r+\frac{1}{M}tr\left(D_{r^{\prime }r^{\prime }}{\tilde{\Sigma }}_{r^{\prime }r^{\prime }}\right)$$

(B90)
$$\frac{\partial g}{\partial g}=0=\frac{-2\alpha C}{(\lambda +q)(\lambda +r)-C^{2}}-2\overset{ˆ}{C}+J$$

(B91)
$$\frac{\partial g}{\partial \overset{ˆ}{c}}=0=-2C+\frac{1}{M}tr\left(D_{rr^{\prime }}{\tilde{\Sigma }}_{r^{\prime }r}+D_{r^{\prime }r}{\tilde{\Sigma }}_{rr^{\prime }}\right)$$

These 6 equations can in principle be solved for $\left\{q,r,c,\overset{ˆ}{q},\overset{ˆ}{r},\overset{ˆ}{c}\right\}$ and their derivatives with respect to J. Note that when J=0, the saddle point equations B90, B91 are solved by setting $c=\overset{ˆ}{c}=0$, and in this case the remaining saddle-point equations decouple over the readouts (as expected for independently trained ensemble members) giving: For readout r :

(B92)
$$0\;=\frac{\alpha }{\left(\lambda +q\right)}-\overset{ˆ}{q}$$

(B93)
$$0\;=-q+\frac{1}{M}tr\left(G_{rr}^{-1}{\tilde{\Sigma }}_{rr}\right)$$

and for readout $r^{\prime }$ :

(B94)
$$0=\frac{\alpha }{\left(\lambda +r\right)}-\overset{ˆ}{r}$$

(B95)
$$0=-r+\frac{1}{M}tr\left(G_{r^{\prime }r^{\prime }}^{-1}{\tilde{\Sigma }}_{r^{\prime }r^{\prime }}\right)$$

These are equivalent to the saddle-point equations for a single readout given in equation B87, B86 as expected for independently trained readouts. It is physically sensible that c=0 when J=0, because at zero source, there is no term in the replicated system energy function which would distinguish the overlap between two readouts from the same replica from the overlap between two readouts in separate replicas (we expect that the total overlap between readouts is non-zero, as we may still have C>0). Differentiating the saddle point equations with respect to J will give a 6 by 6 system of equations for the derivatives of the order parameters. With foresight, we first calculate $\partial_{J}D$

(B96)
$$\partial_{J}D=\partial_{J}G^{-1}=-G^{-1}\left(\partial_{J}G\right)G^{-1}$$

Evaluated at J=0, we have the following:

(B97)
$$\partial_{J}D_{rr}\;=-\partial_{J}\overset{ˆ}{q}G_{rr}^{-1}{\tilde{\Sigma }}_{rr}G_{rr}^{-1}$$

(B98)
$$\partial_{J}D_{r^{\prime }r^{\prime }}\;=-\partial_{J}\overset{ˆ}{r}G_{r^{\prime }r^{\prime }}^{-1}{\tilde{\Sigma }}_{r^{\prime }r^{\prime }}G_{r^{\prime }r^{\prime }}^{-1}$$

(B99)
$$\partial_{J}D_{rr^{\prime }}\;=-\partial_{J}\overset{ˆ}{c}G_{rr}^{-1}{\tilde{\Sigma }}_{rr^{\prime }}G_{r^{\prime }r^{\prime }}^{-1}$$

(B100)
$$\partial_{J}D_{r^{\prime }r^{\prime }}\;=-\partial_{J}\overset{ˆ}{c}G_{r^{\prime }r^{\prime }}^{-1}{\tilde{\Sigma }}_{r^{\prime }r}G_{rr}^{-1}$$

Differentiating equations B86, B87, B88, B89, B90, B91 and evaluating at $J,c,\overset{ˆ}{c}=0$ we get:

(B101)
$$0\;=\partial_{J}\overset{ˆ}{q}+\frac{\alpha }{(\lambda +q{)}^{2}}\partial_{J}q$$

(B102)
$$0\;=\partial_{J}q+\partial_{J}\overset{ˆ}{q}\frac{1}{M}tr\left[{\left(G_{rr}^{-1}{\tilde{\Sigma }}_{rr}\right)}^{2}\right]$$

(B103)
$$0\;=\partial_{J}\overset{ˆ}{r}+\frac{\alpha }{(\lambda +r{)}^{2}}\partial_{J}r$$

(B104)
$$0\;=\partial_{J}r+\partial_{J}\overset{ˆ}{r}\frac{1}{M}tr\left[{\left(G_{r^{\prime }r^{\prime }}^{-1}{\tilde{\Sigma }}_{r^{\prime }r^{\prime }}\right)}^{2}\right]$$

(B105)
$$\frac{1}{2}\;=\partial_{J}\overset{ˆ}{c}+\frac{\alpha }{\left(\lambda +q)(\lambda +r\right)}\partial_{J}c$$

(B106)
$$0\;=\partial_{J}c+\partial_{J}\overset{ˆ}{c}\frac{1}{M}tr\left[G_{rr}^{-1}{\tilde{\Sigma }}_{rr^{\prime }}G_{r^{\prime }r^{\prime }}^{-1}{\tilde{\Sigma }}_{r^{\prime }r}\right]$$

Solving these equations, we obtain:

(B107)
$$\partial_{J}\overset{ˆ}{q}\;=0$$

(B108)
$$\partial_{J}\overset{ˆ}{r}\;=0$$

(B109)
$$\partial_{J}\overset{ˆ}{c}\;=\frac{1}{2(1-\gamma )}$$

(B110)
$$\text{where }\gamma \;\equiv \frac{\alpha }{\left(\lambda +q)(\lambda +r\right)}\frac{1}{M}tr\left[G_{rr}^{-1}{\tilde{\Sigma }}_{rr^{\prime }}G_{r^{\prime }r^{\prime }}^{-1}{\tilde{\Sigma }}_{r^{\prime }r}\right]$$

We may then simplify the expression for the generalization error as follows:

(B111)
$$E_{rr^{\prime }}\;=\frac{\gamma }{1-\gamma }\zeta^{2}+\frac{1}{1-\gamma }\left(\frac{1}{M}w^{*⊤}\Sigma_{s}w^{*}\right)\;\;-\frac{1}{M(1-\gamma )}w^{*⊤}\Sigma_{s}\left[\frac{1}{\nu_{rr}}\overset{ˆ}{q}A_{r}^{⊤}G_{rr}^{-1}A_{r}+\frac{1}{\nu_{r^{\prime }r^{\prime }}}\overset{ˆ}{r}A_{r^{\prime }}^{⊤}G_{r^{\prime }r^{\prime }}^{-1}A_{r^{\prime }}\right]\Sigma_{s}w^{*}\;\;+\frac{1}{M(1-\gamma )}\overset{ˆ}{q}\overset{ˆ}{r}\frac{1}{\sqrt{\nu_{rr}\nu_{r^{\prime }r^{\prime }}}}w^{*⊤}\Sigma_{s}A_{r}^{⊤}G_{rr}^{-1}{\tilde{\Sigma }}_{rr^{\prime }}G_{r^{\prime }r^{\prime }}^{-1}A_{r^{\prime }}\Sigma_{s}w^{*}$$

Re-labeling the order parameters: $\overset{ˆ}{q}\to {\overset{ˆ}{q}}_{r},\overset{ˆ}{r}\to {\overset{ˆ}{q}}_{r^{\prime }},\gamma \to \gamma_{rr^{\prime }}$ and $G_{rr}\to G_{r}$, we obtain the result given in the main text.

## Appendix C: Equicorrelated Data Model

To gain an intuition for the joint effects of correlated data, subsampling, ensembling, feature noise, and readout noise, we simplify the formulas for the generalization error in the following special case:

(C1)
$$\Sigma_{s}=s\left[(1-c)I_{M}+c1_{M}1_{M}^{⊤}\right]$$

(C2)
$$\Sigma_{0}=\omega I_{M}$$

Here s is a parameter which sets the overall scale of the data and c∈[0,1] tunes the correlation structure in the data and ω sets the scale of an isotropic feature noise. We consider an ensemble of k readouts, each of which sees a subset of the features. Due to the isotropic nature of the equicorrelated data model and the pairwise decomposition of the generalization error, we expect that the generalization error will depend on the partition of features among the readout neurons through only:

- The number of features sampled by each readout: $N_{r}\equiv \nu_{rr}M$, for r=1,…,k
- The number of features jointly sampled by each pair of readouts $n_{rr^{\prime }}\equiv \nu_{rr^{\prime }}M$ for $r,r^{\prime }\in \{1,\ldots ,k\}$

Here, we have introduced the subsampling fractions $\nu_{rr}=\frac{N_{r}}{M}$ and the overlap fractions $\nu_{rr^{\prime }}=\frac{n_{rr^{\prime }}}{M}$

We will average the generalization error over readout weights drawn randomly from the space perpendicular to $1_{M}$, with an added spike along the direction of $1_{M}$ :

(C3)
$$w^{*}=\sqrt{1-\rho^{2}}P_{⊥}w_{0}^{*}+\rho 1_{M}$$

(C4)
$$w_{0}^{*}\sim 𝒩\left(0,I_{M}\right)$$

The projection matrix may be written $P_{⊥}=I_{M}-\frac{1}{N}1_{M}1_{M}^{⊤}$. The two components of the ground truth weights will yield independent contributions to the generalization error in the sense that

(C5)
$$\left\langle E_{rr^{\prime }}\right\rangle =\left(1-\rho^{2}\right)E_{rr^{\prime }}(\rho =0)+\rho^{2}E_{rr^{\prime }}(\rho =1)$$

Calculating $E_{rr}$ and $E_{rr^{\prime }}$ is an exercise in linear algebra which is straightforward but tedious. To assist with the tedious algebra, we wrote a Mathematica package which can handle multiplication, addition, and inversion of matrices of symbolic dimension of the specific form encountered in this problem. This form consists of block matrices, where the blocks may be written as $a\delta_{MN}I_{M}+b1_{M}1_{N}^{⊤}$, where a,b are scalars and $\delta_{MN}$ ensures that there is only a diagonal component for square blocks (when M=N). This package is included as supplemental material to this publication.

### 1. Diagonal Terms and Saddle-Point Equations

Here, we solve for the dominant values of $q_{r}$ and ${\overset{ˆ}{q}}_{r}$ and simplify the expressions for $E_{rr}$ in the case of equicorrelated features described above. In this isotropic setting, $E_{rr},q_{r},{\overset{ˆ}{q}}_{r}$ will depend on the subsampling only through $N_{r}=\nu_{rr}M$. We may then write, without loss of generality $A_{r}=\left(\begin{array}{ll} I_{N_{r}} & 0 \end{array}\right)\in R^{N_{r}\times M}$ where **0** denotes a matrix of all zeros, of the appropriate dimensionality.

We start by simplifying the saddle-point equations B87,B86. Expanding $\frac{1}{M}tr\left(G_{r}^{-1}{\tilde{\Sigma }}_{rr}\right)$ and keeping only leading order terms, the saddle-point equations for $q_{r}$ and ${\overset{ˆ}{q}}_{r}$ reduce to:

(C6)
$$q_{r}\;=\frac{\nu_{rr}(s(1-c)+\omega )}{{\overset{ˆ}{q}}_{r}(s(1-c)+\omega )+\nu_{r}}$$

(C7)
$${\overset{ˆ}{q}}_{r}\;=\frac{\alpha }{\lambda +q_{r}}$$

Defining a≡s(1-c)+ω and solving this system of equations, we find:

(C8)
$$q_{r}=\frac{\sqrt{a^{2}\alpha^{2}+2a\alpha (\lambda -a)\nu_{r}+(a+\lambda {)}^{2}\nu_{r}^{2}}-a\alpha +(a-\lambda )\nu_{r}}{2\nu_{r}}$$

(C9)
$${\overset{ˆ}{q}}_{r}=\frac{\sqrt{a^{2}\alpha^{2}+2a\alpha (\lambda -a)\nu_{r}+(a+\lambda {)}^{2}\nu_{r}^{2}}+a\alpha -(a+\lambda )\nu_{r}}{2a\lambda }$$

We have selected the solution with $q_{r}>0$ because self-overlaps must be at least as large as overlaps between different replicas. This solution to the saddle-point equatios can be applied to each of the k readouts.

Next, we calculate $E_{rr}$. Expanding $\gamma_{rr}\equiv \frac{\alpha }{M\kappa^{2}}tr\left[{\left(G^{-1}\tilde{\Sigma }\right)}^{2}\right]$ to leading order in M, we find:

(C10)
$$\gamma_{rr}=\frac{a^{2}\alpha \nu_{r}}{{\left(\lambda +q_{r}\right)}^{2}{\left(a{\overset{ˆ}{q}}_{r}+\nu_{r}\right)}^{2}}$$

(C11)
$${\left\langle E_{rr}\right\rangle }_{𝒟,w^{*}}(\rho =0)=\frac{1}{1-\gamma_{rr}}\frac{1}{M}tr\left[P_{⊥}\left(\Sigma_{s}-\frac{2}{\nu_{rr}}{\overset{ˆ}{q}}_{r}\Sigma_{s}A_{r}^{⊤}G_{r}^{-1}A_{r}\Sigma_{s}+\frac{1}{\nu_{rr}}{\overset{ˆ}{q}}_{r}^{2}\Sigma_{s}A_{r}^{⊤}G_{r}^{-1}\tilde{\Sigma }G_{r}^{-1}A_{r}\Sigma_{s}\right)P_{⊥}\right]\;\;+\frac{\gamma_{rr}}{1-\gamma_{rr}}\zeta^{2}+\eta_{r}^{2},$$

(C12)
$${\left\langle E_{rr}\right\rangle }_{𝒟,w^{*}}(\rho =1)=\frac{1}{1-\gamma_{rr}}\frac{1}{M}1_{M}^{⊤}\left[\Sigma_{s}-\frac{2}{\nu_{rr}}{\overset{ˆ}{q}}_{r}\Sigma_{s}A_{r}^{⊤}G_{r}^{-1}A_{r}\Sigma_{s}+\frac{1}{\nu_{rr}}{\overset{ˆ}{q}}_{r}^{2}\Sigma_{s}A_{r}^{⊤}G_{r}^{-1}\tilde{\Sigma }G_{r}^{-1}A_{r}\Sigma_{s}\right]1_{m}+\frac{\gamma_{rr}}{1-\gamma_{rr}}\zeta^{2}+\eta_{r}^{2},$$

With the aid of our custom Mathematica package, we calculate the traces and contractions in these expressions and expand them to leading order in M, finding:

(C13)
$${\left\langle E_{rr}\right\rangle }_{𝒟,w^{*}}(\rho =0)=\frac{1}{1-\gamma_{rr}}\left(s(1-c)\left(1-\frac{(1-c)s{\overset{ˆ}{q}}_{r}\nu_{r}\left({\overset{ˆ}{q}}_{r}(s(1-c)+\omega )+2\nu_{r}\right)}{{\left({\overset{ˆ}{q}}_{r}(s(1-c)+\omega )+\nu_{r}\right)}^{2}}\right)\right)+\frac{\gamma_{rr}\zeta^{2}+\eta_{r}^{2}}{1-\gamma_{rr}}$$

(C14)
$${\left\langle E_{rr}\right\rangle }_{𝒟,w^{*}}(\rho =1)=\frac{1}{1-\gamma_{rr}}\left(\frac{s(1-c)\left(1-\nu_{rr}\right)+\omega }{\nu_{rr}}\right)+\frac{\gamma_{rr}\zeta^{2}+\eta_{r}^{2}}{1-\gamma_{rr}}$$

In the "ridgeless" limit where λ→0, we obtain:

(C15)
$$\gamma_{rr}=\frac{4\alpha \nu_{rr}}{{\left(\alpha +\nu_{rr}+\left|\alpha -\nu_{rr}\right|\right)}^{2}}$$

(C16)
$${\left\langle E_{rr}(\rho =0)\right\rangle }_{𝒟,w^{*}}=\left\{\begin{array}{ll} \frac{s(1-c)\nu_{rr}}{\nu_{rr}-\alpha }\left(1+\frac{s\alpha (1-c)\left(\alpha -2\nu_{rr}\right)}{\nu_{rr}[s(1-c)+\omega ]}\right)+\frac{\alpha \zeta^{2}+\nu_{rr}\eta_{r}^{2}}{\nu_{rr}-\alpha }, & \text{ if }\alpha <\nu_{rr}\\ \frac{s(1-c)\alpha }{\alpha -\nu_{rr}}\left(1-\frac{s(1-c)\nu_{rr}}{s(1-c)+\omega }\right)+\frac{\nu_{rr}\zeta^{2}+\alpha \eta_{r}^{2}}{\alpha -\nu_{rr}}, & \text{ if }\alpha >\nu_{rr} \end{array}\right\}\;(\lambda \to 0)$$

(C17)
$$\begin{array}{r} {\left\langle E_{rr}(\rho =1)\right\rangle }_{𝒟,w^{*}}=\left\{\begin{array}{l} \frac{\nu_{rr}}{\nu_{rr}-\alpha }\left(\frac{s(1-c)\left(1-\nu_{rr}\right)+\omega }{\nu_{rr}}\right)+\frac{\alpha \zeta^{2}+\nu_{rr}\eta_{r}^{2}}{\nu_{rr}-\alpha },\text{ if }\alpha <\nu_{rr}\\ \frac{\alpha }{\alpha -\nu_{rr}}\left(\frac{s(1-c)\left(1-\nu_{rr}\right)+\omega }{\nu_{rr}}\right)+\frac{\nu_{rr}\zeta^{2}+\alpha \eta_{r}^{2}}{\alpha -\nu_{rr}},\text{ if }\alpha >\nu_{rr} \end{array}\right\}\;(\lambda \to 0) \end{array}$$

### 2. Off-Diagonal Terms

In this section, we calculate the off-diagonal error terms $E_{rr^{\prime }}$ for $r\neq r^{\prime }$, again making use of our custom Mathematica package to simplify contractions of block matrices of the prescribed form. By the isotropic nature of the equicorrelated data model, $E_{rr^{\prime }}$ can only depend on the subsampling scheme through $\nu_{rr},\nu_{r^{\prime }r^{\prime }}$, and $\nu_{rr^{\prime }}$. We can thus, without loss of generality, write:

(C18)
$$A_{r}=\left(\begin{array}{cccc} I_{n_{r}\times n_{r}} & 0_{n_{r}\times n_{r^{\prime }}} & 0_{n_{r}\times n_{s}} & 0_{n_{r}\times l}\\ 0_{n_{s}\times n_{r}} & 0_{n_{s}\times n_{r^{\prime }}} & I_{n_{s}\times n_{s}} & 0_{n_{s}\times l} \end{array}\right)\in R^{N_{r}\times M}$$

(C19)
$$A_{r^{\prime }}=\left(\begin{array}{cccc} 0_{n_{r^{\prime }}\times n_{r}} & I_{n_{r^{\prime }}\times n_{r^{\prime }}} & 0_{n_{r^{\prime }}\times n_{s}} & 0_{n_{r^{\prime }}\times l}\\ 0_{n_{s}\times n_{r}} & 0_{n_{s}\times n_{r^{\prime }}} & I_{n_{s}\times n_{s}} & 0_{n_{s}\times l} \end{array}\right)\in R^{N_{r^{\prime }}\times M}$$

where we have defined $n_{s}$ to be the number of features shared between the readouts, $n_{r}=N_{r}-n_{s}$ and $n_{r^{\prime }}=N_{r^{\prime }}-n_{s}$ and the count of remaining features $l=M-n_{r}-n_{r^{\prime }}-n_{s}$.

Then, to leading order in M, we find:

(C20)
$$\gamma_{rr^{\prime }}=\frac{\alpha \nu_{rr^{\prime }}(s(1-c)+\omega {)}^{2}}{\left(\lambda +q_{r}\right)\left(\lambda +q_{r^{\prime }}\right)\left(\nu_{rr}+(s(1-c)+\omega ){\overset{ˆ}{q}}_{r}\right)\left(\nu_{r^{\prime }r^{\prime }}+(s(1-c)+\omega ){\overset{ˆ}{q}}_{r^{\prime }}\right)}$$

Averaging $E_{rr^{\prime }}$ over $w_{0}^{*}\sim 𝒩\left(0,I_{M}\right)$, we get:

(C21)
$${\left\langle E_{rr^{\prime }}(𝒟)\right\rangle }_{𝒟,w^{*}}(\rho =0)=\frac{\gamma_{rr^{\prime }}}{1-\gamma_{rr^{\prime }}}\zeta^{2}+\frac{1}{1-\gamma_{rr^{\prime }}}\left(\frac{1}{M}tr\left[P_{⊥}\Sigma_{s}P_{⊥}\right]\right)\;\;-\frac{1}{M\left(1-\gamma_{rr^{\prime }}\right)}tr\left[P_{⊥}\Sigma_{s}\left(\frac{1}{\nu_{rr}}{\overset{ˆ}{q}}_{r}A_{r}^{⊤}G_{r}^{-1}A_{r}+\frac{1}{\nu_{r^{\prime }r^{\prime }}}{\overset{ˆ}{q}}_{r^{\prime }}A_{r^{\prime }}^{⊤}G_{r^{\prime }}^{-1}A_{r^{\prime }}\right)\Sigma_{s}P_{⊥}\right]\;\;+\frac{{\overset{ˆ}{q}}_{r}{\overset{ˆ}{q}}_{r^{\prime }}}{M\left(1-\gamma_{rr^{\prime }}\right)}\frac{1}{\sqrt{\nu_{rr}\nu_{r^{\prime }r^{\prime }}}}tr\left[P_{⊥}\Sigma_{s}A_{r}^{⊤}G_{r}^{-1}{\tilde{\Sigma }}_{rr^{\prime }}G_{r^{\prime }}^{-1}A_{r^{\prime }}\Sigma_{s}P_{⊥}\right],$$

(C22)
$${\left\langle E_{rr^{\prime }}(𝒟)\right\rangle }_{𝒟,w^{*}}(\rho =1)=\frac{\gamma_{rr^{\prime }}}{1-\gamma_{rr^{\prime }}}\zeta^{2}+\frac{1}{M\left(1-\gamma_{rr^{\prime }}\right)}\left(1_{M}^{⊤}\Sigma_{s}1_{M}\right)\;\;-\frac{1}{M\left(1-\gamma_{rr^{\prime }}\right)}1_{M}^{⊤}\Sigma_{s}\left(\frac{1}{\nu_{rr}}{\overset{ˆ}{q}}_{r}A_{r}^{⊤}G_{r}^{-1}A_{r}+\frac{1}{\nu_{r^{\prime }r^{\prime }}}{\overset{ˆ}{q}}_{r^{\prime }}A_{r^{\prime }}^{⊤}G_{r^{\prime }}^{-1}A_{r^{\prime }}\right)\Sigma_{s}1_{M}^{⊤}\;\;+\frac{{\overset{ˆ}{q}}_{r}{\overset{ˆ}{q}}_{r^{\prime }}}{M\left(1-\gamma_{rr^{\prime }}\right)}\frac{1}{\sqrt{\nu_{rr}\nu_{r^{\prime }r^{\prime }}}}1_{M}^{⊤}\Sigma_{s}A_{r}^{⊤}G_{r}^{-1}{\tilde{\Sigma }}_{rr^{\prime }}G_{r^{\prime }}^{-1}A_{r^{\prime }}\Sigma_{s}1_{M}$$

Calculating these contractions and traces and expanding to leading order in M, we get:

(C23)
$${\left\langle E_{rr^{\prime }}(𝒟)\right\rangle }_{𝒟,w^{*}}(\rho =0)=\frac{s(1-c)}{1-\gamma_{rr^{\prime }}}\left(1-\frac{s(1-c)\nu_{rr}{\overset{ˆ}{q}}_{r}}{\nu_{rr}+(s(1-c)+\omega ){\overset{ˆ}{q}}_{r}}-\frac{s(1-c)\nu_{r^{\prime }r^{\prime }}{\overset{ˆ}{q}}_{r^{\prime }}}{\nu_{r^{\prime }r^{\prime }}+(s(1-c)+\omega ){\overset{ˆ}{q}}_{r^{\prime }}}\right.\;\left.+\frac{s(1-c)(s(1-c)+\omega )\nu_{rr^{\prime }}{\overset{ˆ}{q}}_{r}{\overset{ˆ}{q}}_{r^{\prime }}}{\left(\nu_{rr}+(s(1-c)+\omega ){\overset{ˆ}{q}}_{r}\right)\left(\nu_{r^{\prime }r^{\prime }}+(s(1-c)+\omega ){\overset{ˆ}{q}}_{r^{\prime }}\right)}\right)\;\;+\frac{\gamma_{rr^{\prime }}}{1-\gamma_{rr^{\prime }}}\zeta^{2}$$

(C24)
$${\left\langle E_{rr^{\prime }}(𝒟)\right\rangle }_{𝒟,w^{*}}(\rho =1)=\frac{1}{1-\gamma_{rr^{\prime }}}\left(\frac{s(1-c)\left(\nu_{rr^{\prime }}-\nu_{rr}\nu_{r^{\prime }r^{\prime }}\right)+\omega \nu_{rr^{\prime }}}{\nu_{rr}\nu_{r^{\prime }r^{\prime }}}\right)+\frac{\gamma_{rr^{\prime }}}{1-\gamma_{rr^{\prime }}}\zeta^{2}$$

Taking λ→0 we get the ridgeless limit:

(C25)
$$\gamma_{rr^{\prime }}\to \frac{4\alpha \nu_{rr^{\prime }}}{\left(\alpha +\nu_{rr}+\left|\alpha -\nu_{rr}\right|\right)\left(\alpha +\nu_{r^{\prime }r^{\prime }}+\left|\alpha -\nu_{r^{\prime }r^{\prime }}\right|\right)}\;(\lambda \to 0)$$

(C26)
$${\left\langle E_{rr^{\prime }}(𝒟)\right\rangle }_{𝒟,w^{*}}(\rho =0)=\frac{1}{1-\gamma_{rr^{\prime }}}F_{0}(\alpha )+\frac{\gamma_{rr^{\prime }}}{1-\gamma_{rr^{\prime }}}\zeta^{2}\;\left(r\neq r^{\prime }\right)$$

where

(C27)
$$\begin{array}{c} F_{0}(\alpha )\equiv \left\{\begin{array}{ll} \frac{(c-1)s\left(\nu_{r}\nu_{r^{\prime }}((2\alpha -1)(c-1)s+\omega )-\alpha^{2}(c-1)s\nu_{rr^{\prime }}\right)}{\nu_{r}((c-1)s-\omega )\nu_{\nu^{\prime }}}, & \text{ if }\alpha \leq \nu_{rr}\leq \nu_{r^{\prime }r^{\prime }}\\ \frac{(c-1)s\left(\nu_{r^{\prime }}\left((c-1)s\nu_{r}+(\alpha -1)(c-1)s+\omega \right)-\alpha (c-1)s\nu_{rr^{\prime }}\right)}{\left.((c-1)s-\omega )\nu_{r^{\prime }}\right)}, & \text{ if }\nu_{rr}\leq \alpha \leq \nu_{r^{\prime }r^{\prime }}\\ \frac{(c-1)s\left((c-1)s\nu_{r^{\prime }}-cs\nu_{rr^{\prime }}+(c-1)s\nu_{r}-cs+s\nu_{rr^{\prime }}+s+\omega \right)}{(c-1)s-\omega }, & \text{ if }\nu_{rr}\leq \nu_{r^{\prime }r^{\prime }}\leq \alpha \end{array}\right\} \end{array}$$

(C28)
$${\left\langle E_{rr^{\prime }}(𝒟)\right\rangle }_{𝒟,w^{*}}(\rho =1)=\frac{1}{1-\gamma_{rr^{\prime }}}\left(\frac{s(1-c)\left(\nu_{rr^{\prime }}-\nu_{rr}\nu_{r^{\prime }r^{\prime }}\right)+\omega \nu_{rr^{\prime }}}{\nu_{rr}\nu_{r^{\prime }r^{\prime }}}\right)+\frac{\gamma_{rr^{\prime }}}{1-\gamma_{rr^{\prime }}}\zeta^{2}\;(\lambda \to 0)$$

### 3. Phase Transitions in Uniform Resource-Constrained Ensembling.

We make further simplifications in the special case where $\omega =\zeta =0,\eta_{r}=\eta ,\nu_{rr}=\frac{1}{k}$ for all r=1,…k, and $\nu_{rr^{\prime }}=0$ for all $r\neq r^{\prime }$. in the ridgeless limit λ→0.

(C29)
$$E_{k}=\frac{1}{k}E_{rr}\left(\nu_{rr}=1/k\right)+\frac{k-1}{k}E_{rr^{\prime }}\left(\nu_{rr}=\nu_{r^{\prime }r^{\prime }}=1/k,\nu_{rr^{\prime }}=0\right)$$

Or, in full:

(C30)
$$E_{k}=\left\{\begin{array}{ll} -\frac{\alpha (c-1)k^{2}s\left(2\alpha \left(\rho^{2}-1\right)-2\rho^{2}+1\right)-(c-1)ks\left(\alpha^{2}\left(\rho^{2}-1\right)-\alpha -\rho^{2}+1\right)+\eta^{2}}{k(\alpha k-1)}, & \text{ if }\alpha \leq \frac{1}{k}\\ \frac{(c-1)(k-1)s\left(\alpha k^{2}\left(\rho^{2}-1\right)+k\left(-\alpha \rho^{2}+\alpha -2\rho^{2}+1\right)+2\left(\rho^{2}-1\right)\right)+\alpha \eta^{2}k^{2}}{k^{2}(\alpha k-1)}, & \text{ if }\alpha \geq \frac{1}{k} \end{array}\right\}\;\left(r\neq r^{\prime }\right)$$

To find the boundary between the signal-dominated and noise-dominated regions, we set $E_{\infty }=E_{1}$, and rearrange to get:

(C31)
$$H=\left\{\begin{array}{ll} \alpha (1-\alpha )\left(1-\rho^{2}\right), & \text{ if }0<\alpha <1\\ \frac{(\alpha -1)\left(1-\rho^{2}\right)}{\alpha }, & \text{ if }\alpha >1 \end{array}\right\}$$

To find the boundary between the intermediate and noisy phases, we set: $E_{k+1}=E_{k}$ and take the limit k→∞, then rearrange to get:

(C32)
$$H=2-\left(\frac{1+2\alpha }{\alpha }\right)\rho^{2}$$

### 4. Infinite Data Limit

In this section we consider the behavior of generalization error in the equicorrelated data model as α→∞ while keeping the λ~𝒪(1). For simplicity, we assume $\nu_{rr^{\prime }}=0$ for $r\neq r^{\prime }$, isotropic features (c=0), no feature noise (ω=0) and uniform readout noise $\eta_{r}=\eta$ as in main text figure 3. This limit corresponds to data-rich learning, where the number of training examples is large relative to the number of model parameters. In this case, the saddle point equations reduce to:

(C33)
$${\overset{ˆ}{q}}_{r}\;\to \frac{\alpha }{\lambda }$$

(C34)
$$q_{r}\;\to \frac{\nu_{rr}\lambda }{\alpha }$$

In this limit, we find that $\gamma_{rr^{\prime }}\to 0$. Using this, we can simplify the generalization error as follows:

(C35)
$$E_{g}=\frac{1}{k^{2}}\sum_{rr^{\prime }=1}^{k}\;E_{rr^{\prime }}=s\left[1-\left(2-\frac{1}{k}\right)\left(\frac{1}{k}\sum_{r=1}^{k}\;\;\nu_{rr}\right)\right]+\frac{\eta^{2}}{k}$$

Interestingly, we find that the readout error in this case depends on the subsampling fractions $\nu_{rr}$ only through their mean. Therefore, with infinite data, there will be no distinction between homogeneous and heterogeneous subsampling.

## Appendix D: Numerical Experiments

Numerical experiments are performed by generating synthetic datasets by drawing data randomly from multivariate Gaussian distributions, assigning feature noise and noisy labels. Writing the training set in terms of a data matrix $\Psi \in R^{M\times P}$ in which column μ consist of the training point $\psi_{\mu }$ and the labels are organized into a column vector y such that $y_{\mu }=y_{\mu }$, the learned weights are calculated as:

(D1)
$$\overset{ˆ}{w}=\Psi {\left(\Psi^{⊤}\Psi +\lambda I_{p}\right)}^{-1}y$$

In the ridgeless case, a pseudoinverse is used:

(D2)
$$\overset{ˆ}{w}=\Psi^{†}y$$

Numerical experiments were performed using the PyTorch library [46]. The code used to perform numerical experiments and generate plots is provided in a zip file with this submission, and will be made publicly available on GitHub upon acceptance of this manuscript. Numerical computations necessary for this work may be performed in a small amount of time (less than one hour) using an Nvidia GPU.

### 1. Details of Heterogeneous Subsampling Theory

In this section, we describe the method used to calculate loss curves for heterogeneous subsampling experiments seen in main text fig. 3. In each trial, Subsampling fractions $\left\{\nu_{1},\ldots ,\nu_{k}\right\}$ are generated according to the following process:

1. Each fraction $\nu_{rr}$ is drawn independently from a Γ distribution with mean $\frac{1}{k}$ and variance $\sigma^{2}.\nu_{rr}\sim \Gamma_{k,\sigma }$
2. The fractions are re-scaled in order to sum to unity: $\nu_{rr}\to \nu_{rr}/\left(\nu_{1}+\cdots +\nu_{k}\right)$

Equivaltly, the fractions $\nu_{rr}$ are drawn from a Dirichlet distribution [40]. Then, the loss curves are calculated from the given fractions $\left\{\nu_{rr}\right\}$ using equations C16, C17, C23, C24. The dotted red lines show the loss curves for 5 single trials. The solid red lines show the average loss curves from 100 trials. Note that we have defined our own convention for the parameterization of the Γ distribution in which the inverse of the mean and the standard deviation are specified. In terms of the standard "shape" and "scale" parameters, we have:

(D3)
$$\Gamma_{k,\sigma }\equiv \Gamma \left(\text{ shape }=(k\sigma {)}^{-2},\text{ scale }=k\sigma^{2}\right)$$

## Appendix E: Code Availability

All Code used in this paper has been made available online (see https://github.com/benruben87/Learning-Curves-for-Heterogeneous-Feature-Subsampled-Ridge-Ensembles.git). This includes code used to perform numerical experiments, calculate theoretical learning curves, and produce plots as well as the custom Mathematica libraries used to simplify the generalization error in the special case of equicorrelated data.
