# Learning Curves for Noisy Heterogeneous Feature-Subsampled Ridge Ensembles

**Published:** 2023-10-31

**Authors:** Benjamin S. Ruben, Cengiz Pehlevan

**Affiliations:** 1 Biophysics Graduate Program, Harvard University Cambridge, MA 02138; 2 Center for Brain Science, Harvard University Cambridge, MA 02138; 3 John A. Paulson School of Engineering and Applied Sciences, Harvard University Cambridge, MA 02138; 4 Kempner Institute for the Study of Natural and Artificial Intelligence, Harvard University Cambridge, MA 02138

## Abstract

Feature bagging is a well-established ensembling method which aims to reduce prediction variance by combining predictions of many estimators trained on subsets or projections of features. Here, we develop a theory of feature-bagging in noisy least-squares ridge ensembles and simplify the resulting learning curves in the special case of equicorrelated data. Using analytical learning curves, we demonstrate that subsampling shifts the double-descent peak of a linear predictor. This leads us to introduce heterogeneous feature ensembling, with estimators built on varying numbers of feature dimensions, as a computationally efficient method to mitigate double-descent. Then, we compare the performance of a feature-subsampling ensemble to a single linear predictor, describing a trade-off between noise amplification due to subsampling and noise reduction due to ensembling. Our qualitative insights carry over to linear classifiers applied to image classification tasks with realistic datasets constructed using a state-of-the-art deep learning feature map.

## 1 Introduction

Ensembling methods are ubiquitous in machine learning practice [1]. A class of ensembling methods (known as attribute bagging [2] or the random subspace method [3]) is based on feature subsampling [2–6], where predictors are independently trained on subsets of the features, and their predictions are combined to achieve a stronger prediction. The random forest method is a popular example [3, 7].

While commonly used in practice, a theoretical understanding of ensembling via feature subsampling is not well developed. Here, we provide an analysis of this technique in the linear ridge regression setting. Using methods from statistical physics [8–12], we obtain analytical expressions for typicalcase generalization error in linear ridge ensembles (proposition 1), and simplify these expressions in the special case of equicorrelated data with isotropic feature noise (proposition 2). The result provides a powerful tool to quickly probe the generalization error of ensembled regression under a rich set of conditions. In section 3, we study the behavior of a single feature-subsampling regression model. We observe that subsampling shifts the location of a predictor’s sample-wise double-descent peak [13–15]. This motivates section 4, where we study ensembles built on predictors which are heterogeneous in the number of features they access, as a method to mitigate double-descent. We demonstrate this method’s efficacy in a realistic image classification task. In section 5 we apply our theory to the trade-off between ensembling and subsampling in resource-constrained settings. We characterize how a variety of factors influence the optimal ensembling strategy, finding a particular significance to the level of noise in the predictions made by ensemble members.

In summary, we make the following contributions:

- Using the replica trick from statistical physics [8, 11], we derive the generalization error of ensembled least-squares ridge regression in a general setting, and simplify the resulting expressions in the tractable special case where features are equicorrelated.
- We demonstrate benefits of heterogeneous ensembling as a robust and computationally efficient regularizer for mitigating double-descent with analytical theory and in a realistic image classification task.
- We describe the ensembling-subsampling trade-off in resource-constrained settings, and characterize the effect of label noise, feature noise, readout noise, regularization, sample size and task structure on the optimal ensembling strategy.

### Related works:

A substantial body of work has elucidated the behavior of linear predictors for a variety of feature maps [14, 16–30]. Several recent works have extended this research to characterize the behavior of ensembled regression using solvable models [24, 31–33]. Additional recent works study the performance of ridge ensembles with example-wise subsampling [34, 35] and simultaneous subsampling of features and examples [32], finding that subsampling behaves as an implicit regularization. Methods from statistical physics have long been used for machine learning theory [10–12, 26, 27, 30, 36, 37]. Relevant work in this domain include [38] which studied ensembling by data-subsampling in linear regression.

## 2 Learning Curves for Ensembled Ridge Regression

We consider noisy ensembled ridge regression in the setting where ensemble members are trained independently on masked versions of the available features. We derive our main analytical formula for generalization error of ensembled linear regression, as well as analytical expressions for generalization error in the special case of equicorrelated features with isotropic noise.

### 2.1 Problem Setup

Consider a training set $𝒟={\left\{{\overset{-}{\psi }}^{\mu },y^{\mu }\right\}}_{\mu =1}^{P}$ of size P. The training examples ${\overset{-}{\psi }}^{\mu }\in R^{M}$ are drawn from a Gaussian distribution with Gaussian feature noise: ${\overset{‾}{\psi }}^{\mu }=\psi^{\mu }+\sigma^{\mu }$, where $\psi^{\mu }\sim 𝒩\left(0,\Sigma_{s}\right)$ and $\sigma^{\mu }\sim 𝒩\left(0,\Sigma_{0}\right)$. Data and noise are drawn i.i.d. so that $E\left[\psi^{\mu }\psi^{\nu ⊤}\right]=\delta_{\mu \nu }\Sigma_{s}$ and $E\left[\sigma^{\mu }\sigma^{\nu ⊤}\right]=\delta_{\mu \nu }\Sigma_{0}$. Labels are generated from a noisy teacher function $y^{\mu }=\frac{1}{\sqrt{M}}w^{*⊤}\psi^{\mu }+\epsilon^{\mu }$ where $\epsilon^{\mu }\sim 𝒩\left(0,\zeta^{2}\right)$. Label noises are drawn i.i.d. so that $E\left[\epsilon^{\mu }\epsilon^{\nu }\right]=\delta_{\mu \nu }\zeta^{2}$.

We seek to analyze the quality of predictions which are averaged over an ensemble of ridge regression models, each with access to a subset of the features. We consider k linear predictors with weights ${\overset{ˆ}{w}}_{r}\in R^{N_{r}},r=1,\ldots ,k$. Critically, we allow $N_{r}\neq N_{r^{'}}$ for $r\neq r^{'}$, which allows us to introduce *structural* heterogeneity into the ensemble of predictors. A forward pass of the model is given as:

(1) $$f\left(\psi \right)=\frac{1}{k}\sum_{r=1}^{k}\;f_{r}\left(\psi \right),\;f_{r}\left(\psi \right)=\frac{1}{\sqrt{N_{r}}}{\overset{ˆ}{w}}_{r}^{⊤}A_{r}\left(\psi +\sigma \right)+\xi_{r}.$$

The model’s prediction f(ψ) is an average over k linear predictors. The “measurement matrices” $A_{r}\in R^{N_{r}\times M}$ act as linear masks restricting the information about the features available to each member of the ensemble. Subsampling may be implemented by choosing the rows of each $A_{r}$ to coincide with the rows of the identity matrix – the row indices corresponding to indices of the sampled features. The feature noise $\sigma \sim 𝒩\left(0,\Sigma_{0}\right)$ and the readout noises $\xi_{r}\sim 𝒩\left(0,\eta_{r}^{2}\right)$, are drawn independently at the execution of each forward pass of the model. Note that while the feature noise is shared across the ensemble, readout noise is drawn independently for each readout: $E\left[\xi_{r}\xi_{r^{'}}\right]=\delta_{rr^{'}}\eta_{r}^{2}$.

The weight vectors are trained separately in order to minimize an ordinary least-squares loss function with ridge regularization:

(2) $${\overset{ˆ}{w}}_{r}=\underset{w_{r}\in R^{N_{r}}}{argmin}\left[\sum_{\mu =1}^{P}\;\;{\left(\frac{1}{\sqrt{N_{r}}}w_{r}^{⊤}A_{r}{\overset{-}{\psi }}^{\mu }+\xi_{r}^{\mu }-y^{\mu }\right)}^{2}+\lambda_{r}\left|w_{r}^{2}\right|\right]$$

Here $\left\{\xi_{r}^{\mu }\right\}$ represents the readout noise which is present during training, and independently drawn: $\xi_{r}^{\mu }\sim 𝒩\left(0,\eta_{r}^{2}\right),E\left[\xi_{r}^{\mu }\xi_{r}^{\nu }\right]=\eta_{r}^{2}\delta_{\mu \nu }$. As a measure of model performance, we consider the generalization error, given by the mean-squared-error (MSE) on ensemble-averaged prediction:

(3) $$E_{g}(𝒟)=E_{\psi ,\sigma ,\left\{\xi_{r}\right\}}\left[{\left(f(\psi )-\frac{1}{\sqrt{M}}w^{*⊤}\psi \right)}^{2}\right]$$

Here, the angular brackets represent an average over the data distribution and noise: $\psi \sim 𝒩\left(0,\Sigma_{s}\right),\;\sigma \sim 𝒩\left(0,\Sigma_{0}\right),\xi_{r}\sim 𝒩\left(0,\eta_{r}^{2}\right)$. The generalization error depends on the particular realization of the dataset 𝒟 through the learned weights $\left\{{\overset{ˆ}{w}}^{*}\right\}$. We may decompose the generalization error as follows:

(4) $$E_{g}(𝒟)=\frac{1}{k^{2}}\sum_{r,r^{'}=1}^{k}\;\;E_{rr^{'}}(𝒟)$$

(5) $$E_{rr^{'}}\left(𝒟\right)\equiv \frac{1}{M}\left[{\left(\frac{1}{\sqrt{\nu_{rr}}}A_{r}^{⊤}{\overset{ˆ}{w}}_{r}-w^{*}\right)}^{⊤}\Sigma_{s}\left(\frac{1}{\sqrt{\nu_{r^{'}r^{'}}}}A_{r^{'}}^{⊤}{\overset{ˆ}{w}}_{r^{'}}-w^{*}\right)\right.\left.\;+\frac{1}{\sqrt{\nu_{rr}\nu_{r^{'}r^{'}}}}{\overset{ˆ}{w}}_{r}^{⊤}A_{r}\Sigma_{0}A_{r^{'}}^{⊤}{\overset{ˆ}{w}}_{r^{'}}+M\delta_{rr^{'}}\eta_{r}^{2}\right]$$

Computing the generalization error of the model is then a matter of calculating $E_{rr^{'}}$ in the cases where $r=r^{'}$ and $r\neq r^{'}$. In the asymptotic limit we consider, we expect that the generalization error concentrates over randomly drawn datasets 𝒟.

### 2.2 Main Result

We calculate the generalization error using the replica trick from statistical physics, and present the calculation in Appendix F. The result of our calculation is stated in proposition 1.

**Proposition 1.** *Consider the ensembled ridge regression problem described in* Section 2.1. *Consider the asymptotic limit where* $M,\;P,\;\left\{N_{r}\right\}\to \infty$ *while the ratios* $\alpha =\frac{P}{M}$ *and* $\nu_{rr}=\frac{N_{r}}{M},\;r=1,\ldots ,k$ *remain fixed. Define the following quantities:*

(6) $${\tilde{\Sigma }}_{rr^{'}}\;\equiv \frac{1}{\sqrt{\nu_{rr}\nu_{r^{'}r^{'}}}}A_{r}\left[\Sigma_{s}+\Sigma_{0}\right]A_{r^{'}}^{⊤}$$

(7) $$G_{r}\;\equiv I_{N_{r}}+{\overset{ˆ}{q}}_{r}{\tilde{\Sigma }}_{rr}$$

(8) $$\gamma_{rr^{'}}\;\equiv \frac{\alpha }{\left.M\left(\lambda_{r}+q_{r}\right)\left(\lambda_{r^{'}}+q_{r^{'}}\right)\right)}tr\left[G_{r}^{-1}{\tilde{\Sigma }}_{rr^{'}}G_{r^{'}}^{-1}{\tilde{\Sigma }}_{r^{'}r}\right]$$

*Then the terms of the average generalization error (eq. 5) may be written as:*

(9) $$\begin{array}{l} \langle E_{rr^{\prime }}{(𝒟)\rangle }_{𝒟}=\frac{\gamma_{rr^{\prime }}\zeta^{2}+\delta_{rr^{\prime }}\eta_{r}^{2}}{1-\gamma_{rr^{\prime }}}+\frac{1}{1-\gamma_{rr^{\prime }}}\left(\frac{1}{M}w^{*⊤}\Sigma_{s}w^{*}\right)\\ -\frac{1}{M\left(1-\gamma_{rr^{\prime }}\right)}w^{*⊤}\Sigma_{s}\left[\frac{1}{\nu_{rr}}{\hat{q}}_{r}A_{r}^{⊤}G_{r}^{-1}A_{r}+\frac{1}{\nu_{r^{\prime }r^{\prime }}}{\hat{q}}_{r^{\prime }}A_{r^{\prime }}^{⊤}G_{r^{\prime }}^{-1}A_{r^{\prime }}\right]\Sigma_{s}w^{*}\\ +\frac{{\hat{q}}_{r}{\hat{q}}_{r^{\prime }}}{M\left(1-\gamma_{rr^{\prime }}\right)}\frac{1}{\sqrt{\nu_{rr}\nu_{r^{\prime }r^{\prime }}}}w^{*⊤}\Sigma_{s}A_{r}^{⊤}G_{r}^{-1}{\tilde{\Sigma }}_{rr^{\prime }}G_{r^{\prime }}^{-1}A_{r^{\prime }}\Sigma_{s}w^{*} \end{array}$$

*where the pairs of order parameters* $\left\{q_{r},{\overset{ˆ}{q}}_{r}\right\}$ *for* r=1,…,K, *satisfy the following self-consistent saddle-point equations*

(10) $${\overset{ˆ}{q}}_{r}=\frac{\alpha }{\lambda_{r}+q_{r}},\;q_{r}=\frac{1}{M}\mathrm{tr}\left[G_{r}^{-1}{\tilde{\Sigma }}_{rr}\right].$$

*Proof*. We calculate the terms in the generalization error using the replica trick, a standard but non-rigorous method from the statistical physics of disordered systems. The full derivation may be found in the Appendix F. When $\Sigma_{s},\Sigma_{0}$ have bounded spectra, this result may be obtained by extending the results of [31] to include readout noise, as shown in Appendix G.

We make the following remarks:

- *Remark* 1. This result applies for any (well-behaved) linear masks $\left\{A_{r}\right\}$. We will focus on the case where each $A_{r}$ implements subsampling of an extensive fraction $\nu_{rr}$ of the features.
- *Remark* 2. When k=1, our result reduces to the generalization error of a single ridge regression model, as studied in refs. [36, 39].
- *Remark* 3. We include “readout noise” which independently corrupts the predictions of each ensemble member. This models sources of variation between ensemble members not otherwise accounted for. For example, ensembles of deep networks will vary due to random initialization of parameters [24, 31, 36]. Readout noise is more directly present in physical neural networks, such as an analog neural networks [40] or biological neural circuits[41] due to their inherent stochasticity.

**Figure 1:**
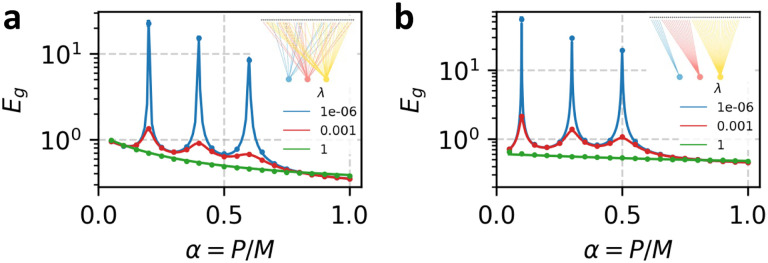
Comparison of numerical and theoretical learning curves for ensembled linear regression. Circles represent numerical results averaged over 100 trials; lines indicate theoretical predictions. Error bars represent the standard error of the mean but are often smaller than the markers. (a) Testing of proposition 1 with $M=2000,{\left[\Sigma_{s}\right]}_{ij}=.8^{\left|i-j\right|},{\left[\Sigma_{0}\right]}_{ij}=\frac{1}{10}(0.3{)}^{\left|i-j\right|},\zeta =0.1$, and all $\eta_{r}=0.2$ and $\lambda_{r}=\lambda$ (see legend). k=3 linear predictors access fixed, randomly selected (with replacement) subsets of the features with fractional sizes $\nu_{rr}=0.2,\;0.4,\;0.6$. Fixed ground-truth weights $w^{*}$ are drawn from an isotropic Gaussian distribution. (b) Testing of proposition 2 with $M=5000,\;s=1,\;c=0.6,\omega^{2}=0.1,\;\zeta =0.1$, all $\eta_{r}=0.1$, and all $\lambda_{r}=\lambda$ (see legend). Ground truth weights sampled as in eq. 11 with ρ=0.3. Feature subsets accessed by each readout are mutually exclusive (inset) with fractional sizes $\nu_{rr}=0.1,\;0.3,\;0.5$.

In Figure 1a, we confirm the result of the general calculation by comparing with numerical experiments using a synthetic dataset with M=2000 highly structured features (see caption for details). k=3 readouts see random, fixed subsets of features. Theory curves are calculated by solving the fixed-point equations 10 numerically for the chosen $\Sigma_{s},\Sigma_{0}$ and ${\left\{A_{r}\right\}}_{r=1}^{k}$ then evaluating eq. 9.

### 2.3 Equicorrelated Data

Our general result allows the freedom to tune many important parameters of the learning problem: the correlation structure of the dataset, the number of ensemble members, the scales of noise, etc. However, the derived expressions are rather opaque. In order to better understand the phenomena captured by these expressions, we examine the following special case:

**Proposition 2.** *In the setting of* section 2.1 *and proposition 1, consider the following special case:*

(11) $$w^{*}\;=\sqrt{1-\rho^{2}}P_{⊥}w_{0}^{*}+\rho 1_{M}$$

(12) $$w_{0}^{*}\;\sim 𝒩\left(0,I_{M}\right)$$

(13) $$\Sigma_{s}\;=s\left[(1-c)I_{M}+c1_{M}1_{M}^{⊤}\right]$$

(14) $$\Sigma_{0}\;=\omega^{2}I_{M}$$

*with* $c\in \left[0,1\right],\rho \in [-1,1]$. *Label and readout noises* $\zeta ,\eta_{r}\geq 0$ *are permitted. Here* $P_{⊥}=I_{M}-\frac{1}{M}1_{M}1_{M}^{⊤}$ is a projection matrix which removes the component of $w_{0}^{*}$ which is parallel to $1_{M}$. *The matrices* ${\left\{A_{r}\right\}}_{r=1}^{k}$ *have rows consisting of distinct one-hot vectors so that each of the* k *readouts has access to a subset of $N_{r}=\nu_{rr}M$ features. For* $r\neq r^{'}$, *denote by* $n_{rr^{'}}$ *the number of neurons sampled by both* $A_{r}$ *and* $A_{r^{'}}$ *and let* $\nu_{rr^{'}}\equiv n_{rr^{'}}/M$ *remain fixed as* M→∞.

*Define the following quantities:*

(15) $$a\equiv s(1-c)+\omega^{2}\;S_{r}\equiv \frac{{\overset{ˆ}{q}}_{r}}{\nu_{rr}+a{\overset{ˆ}{q}}_{r}},\;\gamma_{rr^{'}}\equiv \frac{a^{2}\nu_{rr^{'}}S_{r}S_{r^{'}}}{\alpha }$$

*The terms of the decomposed generalization error may then be written:*

(16) $${\left\langle E_{rr^{'}}\right\rangle }_{𝒟,w_{0}^{*}}=\frac{1}{1-\gamma_{rr^{'}}}\left(\left(1-\rho^{2}\right)I_{rr^{'}}^{0}+\rho^{2}I_{rr^{'}}^{1}\right)+\frac{\gamma_{rr^{'}}\zeta^{2}+\delta_{rr^{'}}\eta_{r}^{2}}{1-\gamma_{rr^{'}}}$$

*where we have defined*

(17) $$I_{rr^{'}}^{0}\equiv s(1-c)\left(1-s(1-c)\nu_{rr}S_{r}-s(1-c)\nu_{r^{'}r^{'}}S_{r^{'}}+as(1-c)\nu_{rr^{'}}S_{r}S_{r^{'}}\right)$$

(18) $$I_{rr^{'}}^{1}\equiv \left\{\begin{array}{ll} \frac{s(1-c)\left(\nu_{rr^{'}}-\nu_{rr}\nu_{r^{'}r^{'}}\right)+\omega^{2}\nu_{rr^{'}}}{\nu_{rr}\nu_{r^{'}r^{'}}} & \text{ if }0<c\leq 1\\ I_{rr^{'}}^{0} & \text{ if }c=0 \end{array}\right.$$

*and where* $\left\{q_{r},{\overset{ˆ}{q}}_{r}\right\}$ *may be obtained analytically as the solution (with $q_{r}>0$*) *to:*

(19) $$q_{r}=\frac{a\nu_{rr}}{\nu_{rr}+a{\overset{ˆ}{q}}_{r}}\;,\;{\overset{ˆ}{q}}_{r}=\frac{\alpha }{\lambda_{r}+q_{r}}$$

*In the “ridgeless” limit where all* $\lambda_{r}\to 0$, *we may make the following simplifications:*

$$S_{r}\to \frac{2\alpha }{a\left(\alpha +\nu_{rr}+\left|\alpha -\nu_{rr}\right|\right)},\;\gamma_{rr^{'}}\;\to \frac{4\alpha \nu_{rr^{'}}}{\left(\alpha +\nu_{rr}+\left|\alpha -\nu_{rr}\right|\right)\left(\alpha +\nu_{r^{'}r^{'}}+\left|\alpha -\nu_{r^{'}r^{'}}\right|\right)}$$

*Proof*. Simplifying the fixed-point equations and generalization error formulas in this special case is an exercise in linear algebra. The main tools used are the Sherman-Morrison formula [42] and the fact that the data distribution is isotropic in the features so that the form of ${\tilde{\Sigma }}_{rr}$ and ${\tilde{\Sigma }}_{rr^{'}}$ depend only on the subsampling and overlap fractions $\nu_{rr},\nu_{r^{'}r^{'}},\nu_{rr^{'}}$. To aid in computing the necessary matrix contractions we developed a custom Mathematica package which handles block matrices of symbolic dimension, with blocks containing matrices of the form $M=c_{1}I+c_{2}11^{⊤}$. This package and the Mathematica notebook used to derive these results are available online (see Appendix B)

In this tractable special case, c∈[0, 1] is a parameter which tunes the strength of correlations between features of the data. When c=0, the features are independent, and when c=1 the features are always equivalent. s sets the overall scale of the features and the “Data-Task alignment” ρ tunes the alignment of the ground truth weights with the special direction in the covariance matrix (analogous to “task-model” alignment [14, 27]). A table of parameters is provided in Appendix A. In Figure 1b, we test these results by comparing the theoretical expressions for generalization error with the results of numerical experiments, finding perfect agreement.

With an analytical formula for the generalization error, we can compute the optimal regularization parameters $\lambda_{r}$ which minimize the generalization error. These may, in general, depend on both r and the sample size α. Rather than minimizing the error of the ensemble, we may minimize the generalization error of predictions made by the ensemble members independently. We find that this “locally optimal” regularization, denoted $\lambda^{*}$, is independent of α, generalizing results from [14, 43] to correlated data distributions (see Appendix H.3).

## 3 Subsampling shifts the double-descent peak of a linear predictor

Consider a single linear regressor (k=1) which connects to a subset of N=νM features in the equicorrelated data setting of proposition 2. Also setting c=0, s=1, and $\eta_{r}=\omega =0$ and taking the limit λ→0 the generalization error reads:

(21) $${\left\langle E_{g}\right\rangle }_{𝒟,w^{*}}=\left\{\begin{array}{ll} \frac{\nu }{\nu -\alpha }\left[(1-\nu )+\frac{1}{\nu }(\alpha -\nu {)}^{2}\right]+\frac{\alpha }{\nu -\alpha }\zeta^{2}, & \text{ if }\alpha <\nu \\ \frac{\alpha }{\alpha -\nu }[1-\nu ]+\frac{\nu }{\alpha -\nu }\zeta^{2}, & \text{ if }\alpha >\nu \end{array}\right\}$$

**Figure 2:**
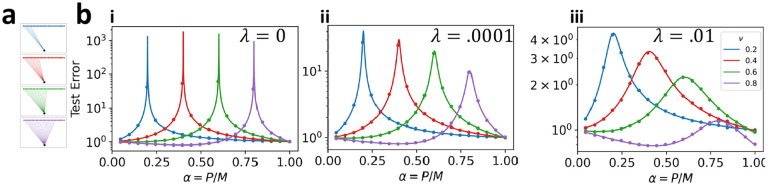
Subsampling alters the location of the double-descent peak of a linear predictor. (a) Illustrations of subsampled linear predictors with varying subsampling fraction ν. (b) Comparison between experiment and theory for subsampling linear regression on equicorrelated datasets. We choose task parameters as in proposition 2 with c=ω=ζ=η=0,s=1, and (i) λ=0, (ii) $\lambda =10^{-4}$, (iii) $\lambda =10^{-2}$. All learning curves are for a single linear predictor k=1 with subsampling fraction ν shown in legend. Circles show results of numerical experiment. Lines are analytical prediction.

We thus see that double descent can arise from two possible sources of variance: explicit label noise (if ζ>0) or implicit label noise induced by feature subsampling (ν<1). As $E_{g}\sim (\alpha -\nu {)}^{-1}$, generalization error diverges when sample size is equal to the number of sampled features. Intuitively, this occurs because subsampling changes the number of parameters of the regression model, and thus its interpolation threshold. To demonstrate this, we plot the learning curves for subsampled linear regression on equicorrelated data in Figure 2. At small finite ridge the test error no longer diverges when α=ν, but still displays a distinctive peak.

## 4 Heterogeneous connectivity mitigates double-descent

Double-descent – over-fitting to noise in the training set near a model’s interpolation threshold – poses a serious risk in practical machine-learning applications [22]. Cross-validating the regularization strength against the training set is the canonical approach to avoiding double-descent [17, 43], but in practice requires a computationally expensive parameter sweep and prior knowledge of the task. In situations where computational resources are limited or hyperparameters are fixed prior to specification of the task, it is natural to seek an alternative solution. Considering again the plots in Figure 2(b), we observe that at any value of α, the double-descent peak can be avoided with an acceptable choice of the subsampling fraction ν. This suggests another strategy to mitigate double descent: heterogeneous ensembling. Ensembling over predictors with a heterogeneous distribution of interpolation thresholds, we may expect that when one predictor fails due to over-fitting, the other members of the ensemble compensate with accurate predictions.

**Figure 3:**
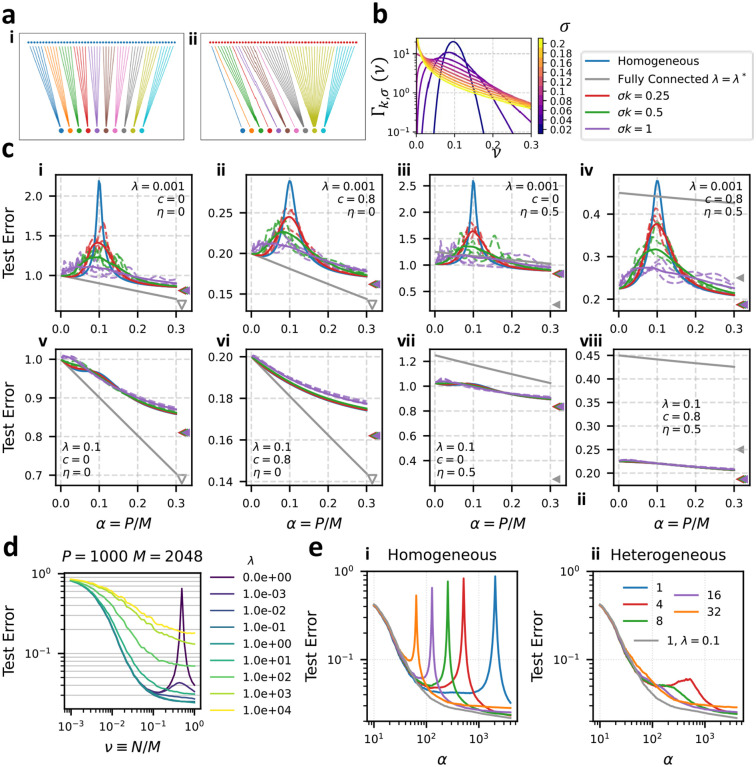
Heterogeneous ensembling mitigates double-descent. (a) We compare (i) homogeneous ensembling, in which k readouts connect to the same fraction ν=1/k of features, and (ii) heterogeneous ensembling (b) In heterogeneous ensembling subsampling fractions are drawn i.i.d. from $\Gamma_{k,\sigma }(\nu )$, shown here for k=10, then re-scaled to sum to 1. (c) Generalization Error Curves for Homogeneous and Heterogeneous ensembling with k=10, ζ=0, ρ=0.3 and indicated values of λ, c, and η. Blue: homogeneous subsampling. Red, green, and purple show heterogeneous subsampling with σ=0.25/k, 0.5/k, 1/k respectively. Dashed lines show learning curves for 3 particular realizations of $\left\{\nu_{11},\ldots ,\nu_{kk}\right\}$. Solid curves show the average over 100 realizations. Gray shows the learning curve for a single linear readout with ν=1 and optimal regularization (eq. 213). Triangular marks show the asymptotic generalization error (α→∞), with downward-pointing gray triangles indicating an asymptotic error of zero. (d,e) Generalization error of linear classifiers applied to the imagewoof dataset with ResNext features averaged over 100 trials. (d) P=100, k=1 varying subsampling fraction ν and regularization λ (legend). (e) Generalization error of (i) homogeneous and (ii) heterogeneous (with σ=0.75/k) ensembles of classifiers. Legend indicates k values. λ=0 except for gray curves, where λ=0.1

**Figure 4:**
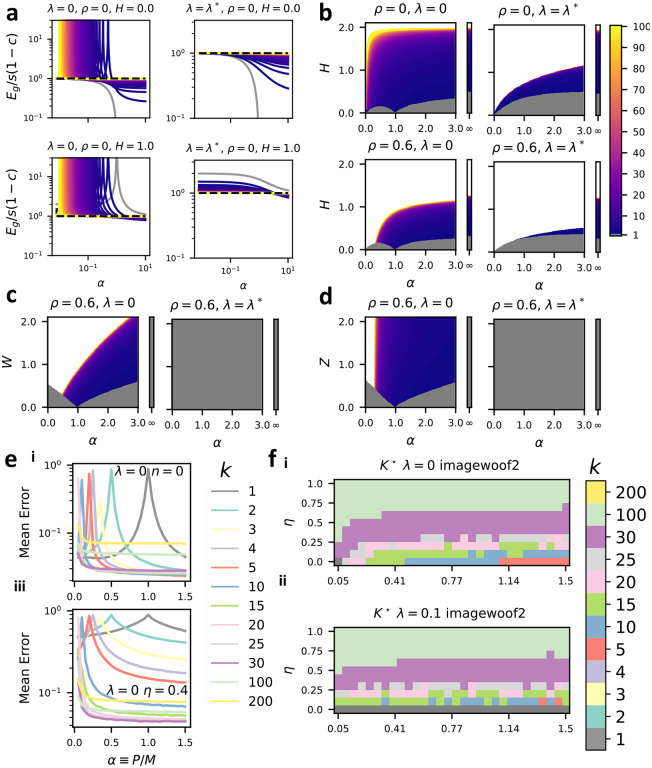
Task parameters dictate the ensembling-subsampling trade-off: (a-d) In the setting of proposition 2 in the special case where all $\nu_{rr^{'}}=\frac{1}{k}\delta_{rr^{'}}$ so that feature subsets are mutually exclusive and the total number of weights is conserved. (a) We plot the reduced generalization errors ℰ (for λ=0, using eq. 22) and ${ℰ}^{*}$ (for $\lambda =\lambda^{*}$ using eq. 23) of linear ridge ensembles of varying size k with ρ=0 and H=0,1 (values indicated above plots). Grey lines indicate k=1, dashed black lines k→∞, and intermediate k values by the colorbar. (b) We plot optimal ensemble size $k^{*}$ (eqs. 25, 26) in the parameter space of sample size α and reduced readout noise scale H setting W=Z=0. Grey indicates $k^{*}=1$ and white indicates $k^{*}\to \infty$, with intermediate values given by the colorbar. Appended vertical bars show α→∞. (c) optimal readout $k^{*}$ phase diagrams as in (b) but showing W-dependence with H=Z=0. (d) optimal readout $k^{*}$ phase diagrams as in (b) but showing Z-dependence with H=W=0. (e) Learning curves for feature-subsampling ensembles of linear classifiers combined using a majority vote rule on the imagewoof classification task (see Appendix E). As in (a-d) we set $\nu_{rr^{'}}=\frac{1}{k}\delta_{rr^{'}}$. Error is calculated as the probability of incorrectly classifying a test example. λ and η values are indicated in each panel. (f) Numerical phase diagrams showing the value of k which minimizes test error in the parameter space of sample size P and readout noise scale η, with regularization (i) λ=0 (pseudoinverse rule) (ii) λ=0.1.

In Figure 3, we show that heterogeneous ensembling can guard against double-descent. We define two ensembling strategies: in homogeneous ensembling, each of k readouts connects a fraction $\nu_{rr}=1/k$ features. In heterogeneous ensembling, the number of features connected by each of the k readouts are drawn from a Gamma distribution $\Gamma_{k,\sigma }(\nu )$ with mean 1/k and standard deviation σ (see Fig. 3b) then r-scaled to sum to 1 (see Appendix C for details). All feature subsets are mutually exclusive ($\nu_{rr^{'}}=0$ for $r\neq r^{'}$). Homogeneous and heterogeneous ensembling are illustrated for k=10 in Figs. 3 a.i and 3 a.ii respectively. We test this hypothesis using eq. 16 in 3c. At small regularization (λ=.001), we find that heterogeneity of the distribution of subsampling fractions (σ>0) lowers the double-descent peak of an ensemble of linear predictors, while at larger regularization (λ=0.1), there is little difference between homogeneous and heterogeneous learning curves. The asymptotic (α→∞) error is unaffected by the presence of heterogeneity in the degrees of connectivity, which can be seen as the coincidence of the triangular markers in Fig. 3c, as well as from the α→∞ limit of eq. 16 (see Appendix H.5). Fig. 3c also shows the learning curve of a single linear predictor with no feature subsampling and optimal regularization. We see that the feature-subsampling ensemble appreciably outperforms the fully-connected model when c=0.8 and η=0.5, suggesting the important roles of data correlations and readout noise in determining the optimal readout strategy. These roles are further explored in section 5 and fig 4.

We also test the effect of heterogeneous ensembling in the a realistic classification task. Specifically, we train ensembles of linear classifiers to predict the labels of imagenet [44] images corresponding to 10 different dog breeds (the “Imagewoof” task [45]) from their top-hidden-layer representations in a pre-trained ResNext deep network [46] (see Appendix E for details). We characterize the statistics of the resulting M=2048-dimensional feature set in Fig. S1. This “ResNext-Imagewoof” classification task has multiple features which make it amenable to learning with a feature-subsampling ensemble. First, the ResNext features have a high degree of redundancy [47], allowing classification to be performed accurately using only a fraction of the available features (see Fig. 3d and S1c). Second, when classifications of multiple predictors are combined by a majority vote, there is a natural upper bound on the influence of a single erring ensemble member (unlike in regression where predictions can diverge). Calculating learning curves for the imagewoof classification task using homogeneous ensembles, we see sharp double-descent peaks in an ensemble of size k when P=M/k (Fig. 3e.i). Using a heterogeneous ensemble mitigates this catastrophic over-fitting, leading to monotonically decreasing error without regularization (Fig. 3e.ii). A single linear predictor with a tuned regularization of λ=0.1 performs only marginally better than the heterogeneous feature-subsampling ensemble with k=16 or k=32. This suggests heterogeneous ensembling can be an effective alternative to regularization in real-world classification tasks using pre-trained deep learning feature maps.

Note that training a feature-subsampling ensemble also benefits from improved computational complexity. Training an estimator of dimension $N_{r}$ involves, in the worst case, inverting an $N_{r}\times N_{r}$ matrix, which requires $𝒪\left(N_{r}^{3}\right)$ operations. Setting $N_{r}=M/k$, we see that the number of operations required to train an ensemble of k predictors scales as $𝒪\left(k^{-2}\right)$.

## 5 Correlations, Noise, and Task Structure Dictate the Ensembling-Subsampling Trade-off

In resource-constrained settings, one must decide between training a single large predictor or an ensemble of smaller predictors. When the number of weights is constrained, ensembling may benefit generalization by averaging over multiple predictions, but at the expense of each prediction incorporating fewer features. Intuitively, the presence of correlations between features limits the penalty incurred by subsampling, as measurements from a subset of features will also confer information about the unsampled features. The equicorrelated data model of proposition 2 permits a solvable toy model for these competing effects. We consider the special case of ensembling over k readouts, each connecting the same fraction $\nu_{rr}=\nu =1/k$ of all features. For simplicity, we set $\nu_{rr^{'}}=0$ for $r\neq r^{'}$. We asses the learning curves of this toy model in both the ridgeless limit λ→0 where double-descent has a large effect on test error, and at ‘locally optimal’ regularization $\lambda =\lambda^{*}$ for which double-descent is eliminated. In these special cases, one can write the generalization error in the following forms (see Appendix H.4 for derivation):

(22) $$E_{g}(k,s,c,\eta ,\omega ,\zeta ,\rho ,\alpha ,\lambda =0)=s(1-c)ℰ(k,\rho ,\alpha ,H,W,Z)$$

(23) $$E_{g}\left(k,s,c,\eta ,\omega ,\zeta ,\rho ,\alpha ,\lambda =\lambda^{*}\right)=s(1-c){ℰ}^{*}(k,\rho ,\alpha ,H,W,Z)$$

where we have defined the effective noise-to-signal ratios:

(24) $$H\equiv \frac{\eta^{2}}{s(1-c)},\;W=\frac{\omega^{2}}{s(1-c)},\;Z=\frac{\zeta^{2}}{s(1-c)}$$

Therefore, given fixed parameters s, c, ρ, α, the value $k^{*}$ which minimizes error depends on the noise scales, s, and c only through the ratios H, W and Z:

(25) $$k_{\lambda =0}^{*}(H,W,Z,\rho ,\alpha )\equiv \underset{k\in N}{argmin}E_{g}(k)=\underset{k\in N}{argmin}ℰ(k,\rho ,\alpha ,H,W,Z)$$

(26) $$k_{\lambda =\lambda^{*}}^{*}(H,W,Z,\rho ,\alpha )\equiv \underset{k\in N}{argmin}E_{g}(k)=\underset{k\in N}{argmin}{ℰ}^{*}(k,\rho ,\alpha ,H,W,Z)$$

In Fig. 4a, we plot these reduced errors curves $ℰ,{ℰ}^{*}$ as a function of α for varying ensemble sizes k and reduced readout noise scales H. At zero regularization learning curves diverge at their interpolation threshold. At locally optimal regularization $\lambda =\lambda^{*}$, learning curves decrease monotonically with sample size. Increasing readout noise H raises generalization error more sharply for smaller k. In Fig. 4b we plot the optimal $k^{*}$ in various two-dimensional slices of parameter space in which ρ is fixed and W=Z=0 while α and H vary. The resulting phase diagrams are naturally divided into three regions. In the signal-dominated phase a single fully-connected readout is optimal $\left(k^{*}=1\right)$. In an intermediate phase, $1<k^{*}<\infty$ minimizes error. And in a noise-dominated phase $k^{*}=\infty$. These three phases persist even when $\lambda =\lambda^{*}$, removing the effects of double descent. In all panels, an increase in H causes an increase in $k^{*}$. This can occur because of a decrease in the signal-to-readout noise ratio $s/\eta^{2}$, or through an increase in the correlation strength c. An increase in ρ also leads to an increase in $k^{*}$, indicating that ensembling is more effective for easier tasks. Figs 4c, d show analogous phase diagrams where W or Z are varied. Signal-dominated, intermediate, and noise-dominated regimes are visible in the resulting phase diagrams at zero regularization. However, when optimal regularization is used, $k^{*}=1$ is always optimal. The presence of regions where $k^{*}>1$ can thus be attributed to double-descent at sub-optimal regularization or to the presence of readout noise which is independent across predictors. We chart the parameter-space of the reduced errors and optimal ensemble size $k^{*}$ extensively in Appendix I. We plot learning curves for the “ResNext-Imagewoof” ensembled linear classification task with varying strength of readout noise in Fig. 4e, and phase diagrams of optimal ensemble size k in Fig. 4f, finding similar behavior to the toy model. See Figs. S3, S4, S5 and Appendix E.4.3 for further discussion.

## 6 Conclusion

In this paper, we provided a theory of feature-subsampled linear ridge regression. We identified the special case in which features of the data are “equicorrelated” as a minimal toy model to explore the combined effects of subsampling, ensembling, and different types of noise on generalization error. The resulting learning curves displayed two potentially useful phenomena.

First, we demonstrated that heterogeneous ensembling can mitigate over-fitting, reducing or eliminating the double-descent peak of an under-regularized model. In most machine learning applications, the size of the dataset is known at the outset and suitable regularization may be determined to mitigate double descent, either by selecting a highly over-parameterized model [22] or by cross-validation techniques (see for example [17]). However, in contexts where a single network architecture is designed for an unknown task or a variety of tasks with varying structure and noise levels, heterogeneous ensembling may be used to smooth out the perils of double-descent.

Next, we described a trade-off between noise reduction due to ensembling and noise amplification due to subsampling in a resource-constrained setting where the total number of weights is fixed. Our analysis suggests that ensembling is particularly useful in neural networks with an inherent noise. Physical neural networks, such as analog neural networks[40] and biological neural circuits [41] present such a resource-constrained environments where intrinsic noise is a significant issue.

Much work remains to achieve a full understanding of the interactions between data correlations, readout noise, and ensembling. In this work, we have given a thorough treatment of the convenient special case where features are equicorrelated. Future work should analyze subsampling and ensembling for codes with realistic correlation structure, such as the power-law spectra (see Fig. S1) [27, 30, 48, 49] and sparse activation patterns [50].

## Supplementary Material

1
