# Supplementary material for: Learning Curves for Noisy Heterogeneous Feature-Subsampled Ridge Ensembles
Source: ArXiv. 2024 Jan 9:arXiv:2307.03176v3. Originally published 2023 Jul 6. Preprint. [Version 3] (PMC10350086)
Supplement: 1 [file NIHPP2307.03176V3-supplement-1.pdf]

A Table of Parameters from Proposition 2 and Figures 2,3,4

Data Scale	s
Correlation Strength	c
Data-Task Alignment	ρ
Readout Noise Scale	η
Feature noise scale	ω
Label Noise Scale	ζ
Ensemble Size	k
Subsampling Fractions	$\nu_{rr} \quad r = 1, \ldots, K$
Subsampling Overlap Fractions	$\nu_{rr'} \quad r \neq r'$
Sample Size	$P \to \infty$
Data Dimensionality	$M \to \infty$
Sample Complexity	$\alpha \equiv \frac{P}{M}$ (finite)
Effective Noise-To-Signal Ratios	$H \equiv \frac{\eta^2}{s(1-c)}, W \equiv \frac{\omega^2}{s(1-c)}, Z \equiv \frac{\zeta^2}{s(1-c)}$

B Code Availability and Compute

All Code used in this paper has been made available online (see https://github.com/benruben87/Learning-Curves-for-Heterogeneous-Feature-Subsampled-Ridge-Ensembles.git). This includes code used to perform numerical experiments, calculate theoretical learning curves, and produce plots as well as the custom Mathematica libraries used to simplify the generalization error in the special case of equicorrelated data. The compute time required to do all of the calculations in this paper is approximately 3 GPU days.

C Homogeneous and Heterogeneous Subsampling

In this section, we describe the homoegeneous and heterogeneous subsampling strategies, as used in this work, in detail. This sampling method was used in calculating the theoretical loss curves for heterogeneous subsampling experiments seen in main text Fig. 3, and in the heterogeneous subsampling experiments applied to the ResNext-features-based image classification task in Figs. 3e, S2.

In homogeneous ensembling, the subsampling fractions $\nu_{rr} = N_r/M$ are chosen as $\nu_{rr} = 1/k$ for all $r = 1, \ldots, k$. In heterogeneous ensembling, the subsampling fractions $\{\nu_{11}, \ldots, \nu_{kk}\}$ are generated according to the following statistical process:

1. Each fraction ν_{rr} is drawn independently as $\nu_{rr} \sim \Gamma_{k,\sigma}$, where a $\Gamma_{k,\sigma}$ represents a Gamma distribution with mean $\frac{1}{k}$ and variance σ^2.
2. The fractions are re-scaled in order to sum to 1: $\nu_{rr} \to \nu_{rr}/(\nu_1 + \cdots + \nu_k)$

Equivalently, the subsampling fractions are drawn from a Dirichlet distribution parameterized by the ensemble size k and a chosen variance σ as $(\nu_{11}, \ldots, \nu_{kk}) \sim \mathrm{Dir}((\sigma k)^{-2}, \ldots, (\sigma k)^{-2})$ [51].

In main text Fig. 3c, we combine this sampling strategy with theoretical learning curves for the equicorrelated data model in a quasi-numerical experiment. At each trial of the experiment, we draw a particular realization of the subsampling fractions $\{\nu_{11}, \ldots, \nu_{kk}\}$, then use the analytical expression (eq. 16) to calculate the resulting learning curve. Dotted lines show the loss curves for 3 single trials, corresponding to three particular realizations of the subsampling fractions. The solid lines show the average over 100 trials.

Note that we have defined our own convention for the parameterization of the Γ distribution in which the inverse of the mean and the standard deviation are specified. In terms of the standard "shape" and "scale" parameters, we have:

$$\Gamma_{k,\sigma} \equiv \Gamma\left(\text{shape } = (k\sigma)^{-2}, \text{scale } = k\sigma^2\right) \tag{27}$$

D Numerical Linear Regression with Synthetic Datasets

Numerical experiments were performed using the PyTorch library [52]. The code used to perform numerical experiments and generate plots has been made publicly available (see section B).

In numerical regression experiments, synthetic datasets with label noise are constructed as described in section 2.1, drawing data randomly from multivariate Gaussian distributions and adding label noise (see "DatasetMaker.py" in available code). Representing the training set in terms of a data matrix $\boldsymbol{\Psi} \in \mathbb{R}^{M \times P}$ in which column μ consist of the training point $\boldsymbol{\psi}_\mu$, and the labels with a column vector $\boldsymbol{y}$ such that $\boldsymbol{y}_\mu = y_\mu$, the learned weights are calculated as:

$$\hat{\boldsymbol{w}} = \boldsymbol{\Psi} \left(\boldsymbol{\Psi}^\top \boldsymbol{\Psi} + \lambda \boldsymbol{I}_p \right)^{-1} \boldsymbol{y} \tag{28}$$

In the ridgeless case, a pseudoinverse is used:

$$\hat{\boldsymbol{w}} = \boldsymbol{\Psi}^\dagger \boldsymbol{y} \tag{29}$$

E Ensembled Linear Classification of Imagenet Images

In this section, we provide the details of numerical experiments which demonstrate that qualitative insights gained from our analysis of the linear regression task with Gaussian data carries over to a practical machine learning task. In particular, we apply ensembles of linear classifiers to datasets constructed using a pre-trained ResNext [46] a specific type of Convolutional Neural Network (CNN).

E.1 Dataset Construction

To construct the dataset, we start with a set of n images $\{\boldsymbol{x}^\mu\}_{\mu=1}^n$ from a subset of $C = 10$ classes of the imagenet dataset. For each image $\boldsymbol{x}^\mu$, we obtain a corresponding feature vector $\boldsymbol{\psi}^\mu \in \mathbb{R}^M$ as the last-hidden-layer activation of the ResNext [46], which has been pre-trained on the imagenet classification task [44]. The architecture we use produces $M = 2048$ features per image. These features will serve as the data input to the downstream linear classifier. The corresponding labels $\boldsymbol{y}^\mu \in \mathbb{R}^C$ are one-hot vectors.

We construct two datasets using this method, using images from the "Imagenette" and "Imagewoof" datasets [45]. For the "Imagenette" task, we a construct a training set of size $n_{tr} = 9469$ and a test set of size $n_{test} = 3925$ containing features corresponding to images from 10 unrelated classes (tench, English springer, cassette player, chain saw, church, French horn, garbage truck, gas pump, golf ball, parachute). For the "Imagewoof" task, we a construct a training set of size $n_{tr} = 9025$ and a test set of size $n_{test} = 3929$ containing features corresponding to images of 10 different dog breeds (Australian terrier, Border terrier, Samoyed, Beagle, Shih-Tzu, English foxhound, Rhodesian ridgeback, Dingo, Golden retriever, Old English sheepdog). The imagewoof classification task is naturally more difficult. The statistics of the resulting datasets are described in Fig. S1, where we plot the data-data covariance matrix, feature-feature covariance matrix, and the eigenvalue spectrum for both the "Imagenette" and "Imagewoof" tasks.

E.2 Model Training

At training time a dataset of P examples is constructed: $\mathcal{D} = \{\boldsymbol{\psi}^\mu, \boldsymbol{y}^\mu\}_{\mu=1}^P$. We represent the training set with a "design matrix" $\boldsymbol{\Phi} \in \mathbb{R}^{P \times M}$ and a label matrix $\boldsymbol{Y} \in \{0, 1\}^{P \times C}$. The loss function for each ensemble member is generalized to multi-class regression. With ridge regularization, the objective for each ensemble member r becomes:

$$\hat{\boldsymbol{W}}_r = \underset{\boldsymbol{W}_r \in \mathbb{R}^{N_r \times C}}{\arg\min} \left[\sum_{\mu=1}^P \| \frac{1}{\sqrt{N_r}} \boldsymbol{A}_r \bar{\boldsymbol{\psi}}^\mu \boldsymbol{W}_r + \boldsymbol{\xi}_r^\mu - \boldsymbol{y}^\mu \|_2^2 + \lambda_r \|\boldsymbol{W}_r\|_F^2 \right],$$

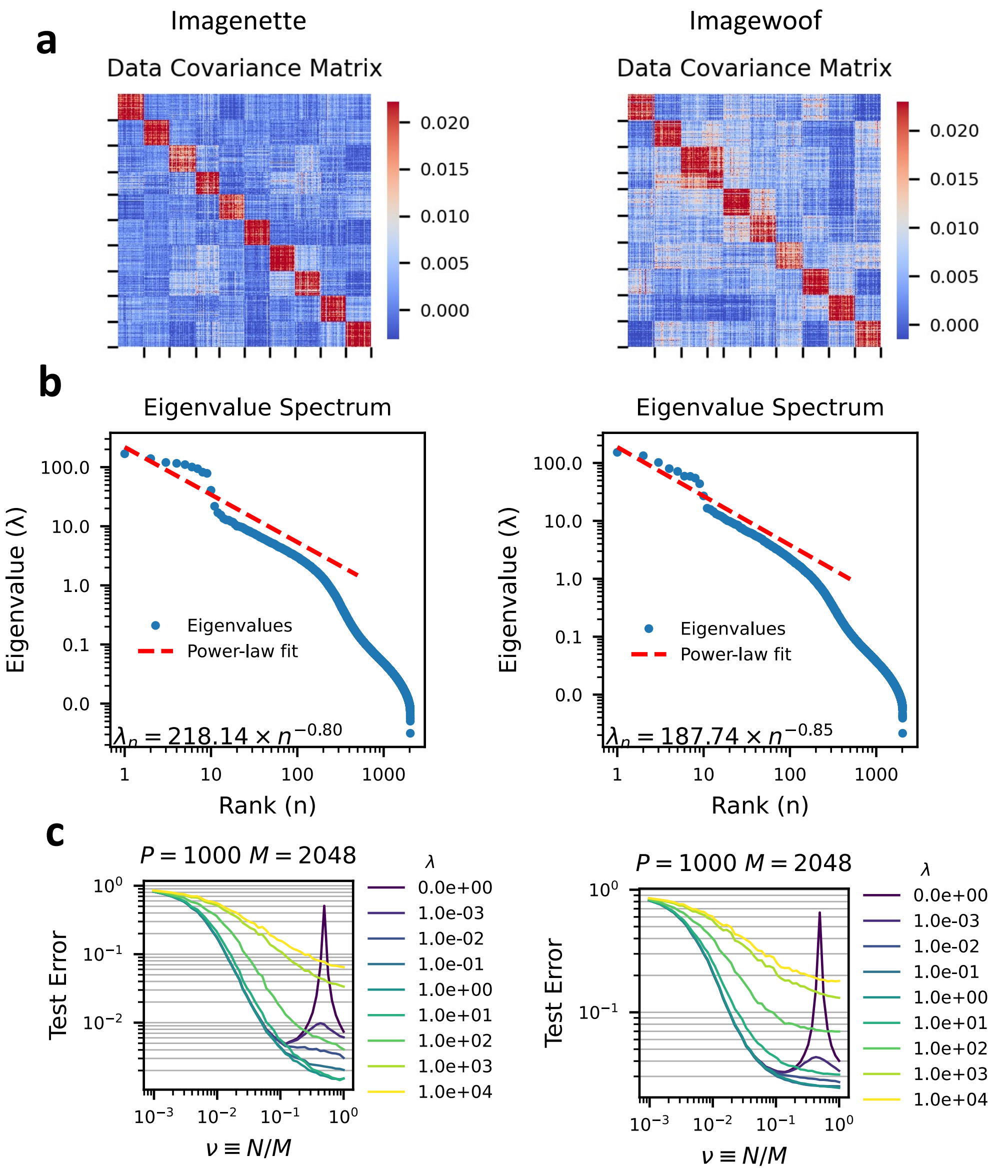

Figure S1: In numerical experiments, we train linear classifiers to predict labels of imagenet images based on their last-hidden-layer representations in a pre-trained RexNext deep learning architecture [46]. Here, we show the structure of the datasets constructed using the ResNext feature map for the Imagenette task (left), which consists of categorizing images from 10 unrelated categories, and the Imagewoof task (right), which consists of categorizing images from 10 different dog breeds. (a) Gram matrix of the centered ResNext features defined as $\frac{1}{P}\left(\mathbf{\Phi}-\bar{\mathbf{\Phi}}\right)^{\top}\left(\mathbf{\Phi}-\bar{\mathbf{\Phi}}\right)$ for data matrix $\Phi \in \mathbb{R}^{P\times M}$ where P is the total size of the dataset. Dataset is sorted by label and tick marks show the boundaries between classes. (b) The covariance eigenspectrum of the ResNext features is well described by a power law decay. (c) Generalization error of Linear classification with a single linear predictor with access to a fraction $\nu = N/M$ of the ResNext features averaged over 100 trials (see discussion in section E.4.1)

where $\|\cdot\|_F$ denotes the Frobenius norm and where the readout noise vector $\boldsymbol{\xi}_r^\mu \sim \mathcal{N}(0, \eta^2 \boldsymbol{I}_C)$ is a C - dimensional readout noise which corrupts the model's prediction. The weights $\hat{\boldsymbol{W}}_r$ can be determined in closed form as follows:

$$\hat{\boldsymbol{W}}_r = \frac{1}{\sqrt{N_r}} \left(\frac{1}{N_r} \boldsymbol{A}_r \boldsymbol{\Phi}^\top \boldsymbol{\Phi} \boldsymbol{A}_r^\top + \lambda_r \boldsymbol{I} \right)^{-1} \left(\boldsymbol{A}_r \boldsymbol{\Phi}^\top (\boldsymbol{Y} - \boldsymbol{\Xi}_r) \right).$$

where we have defined $[\boldsymbol{\Xi}_r]_{\mu c} = [\boldsymbol{\xi}_r^\mu]_c$. When $\lambda = 0$ we instead use a pseudoinverse rule. In all experiments presented here, we use measurement matrices $\boldsymbol{A}_r$ which implement an ordinary subsampling of the features, so that the rows and columns of $\boldsymbol{A}_r$ consist of one-hot vectors.

E.3 Model Prediction

Once trained, the learned weights may be used to predict the label of a new example $\boldsymbol{\psi}$ as follows. For $r = 1, \ldots, k$ we calculate the prediction of each ensemble member by first assigning each class a "score". The scores of predictor r are stored in a vector $\boldsymbol{f}_r \in \mathbb{R}^C$:

$$\boldsymbol{f}_r(\boldsymbol{\psi}) = \frac{1}{\sqrt{N_r}} \boldsymbol{A}_r \boldsymbol{\psi} \hat{\boldsymbol{W}}_r + \boldsymbol{\xi}_r$$

Where $\boldsymbol{\xi}_r \sim \mathcal{N}(0, \eta^2 \boldsymbol{I}_C)$ is drawn randomly at model evaluation. Each ensemble member's "vote" corresponds to the class with the largest score. The prediction of the ensemble is then calculated as a majority vote of the ensemble members. Generalization error is then calculated as the probability of misclassifying an example from the test set.

E.4 Linear Classification Experiments

We apply the described majority-vote linear classifier ensembles to the ResNext-Imagenette and ResNext-Imagewoof tasks in three different experiments.

E.4.1 Reduncancy of ResNext Features

In the first experiment, we investigate the performance of a single linear classifier ($k = 1$) as the fraction of features ν which it has access to varies. We set $P = 1000$ and vary ν over 50 values on a logarithmic scale from 10^{-3} to 1. We also vary the regularization strength over 0 and a logarithmic scale from 10^-3 to 10^4. We average over 100 trials. At each trial the particular subset of $P = 1000$ training examples and the particular subset of $\nu * M$ features is randomly re-sampled. We find that ResNext features are highly redundant – classification accuracy is very robust to subsampling of the features. For example, in the Imagewoof classification task with the best regularization tested ($\lambda = 0.1$), test error increases from about 1% to about 2% as the subsampling fraction decreases from $\nu = 1$ to $\nu = 0.1$ (meaning 90% of the features are ignored) (see Fig. 3d). Similarly, for the imagenette task, test error increases from about 0.1% to about 0.2% as the subsampling fraction decreases from $\nu = 1$ to $\nu = 0.1$ (see Fig. S1c). Code used to run these experiments may be found in the folder "DeepNet_Subsamp" in the GitHub repository.

E.4.2 Heterogeneous Ensembling Mitigates Double-Descent

In the second experiment, we compare learning curves for homogeneous ensembling and heterogeneous ensembling applied to the ResNext-Imagewoof classification task. In each trial, we train ensembles of $k = \{1, 4, 8, 16, 32\}$ linear predictors whose subsampling fractions $\{\nu_{rr}, \ldots, \nu_{kk}\}$ are assigned either with Homogeneous ensembling or with Heterogeneous Ensembling with $\sigma = 0.75/k$ C. After subsampling fraction are assigned, the training set is randomly shuffled. We iterate over 50 sample sizes P logarithmically distributed from 400 to 4000, and then add the values M/k for each k to the list of P values. We repeat for 100 trials for both $\lambda_r = 0$ (pseudoinverse rule) and $\lambda = 0.1$,

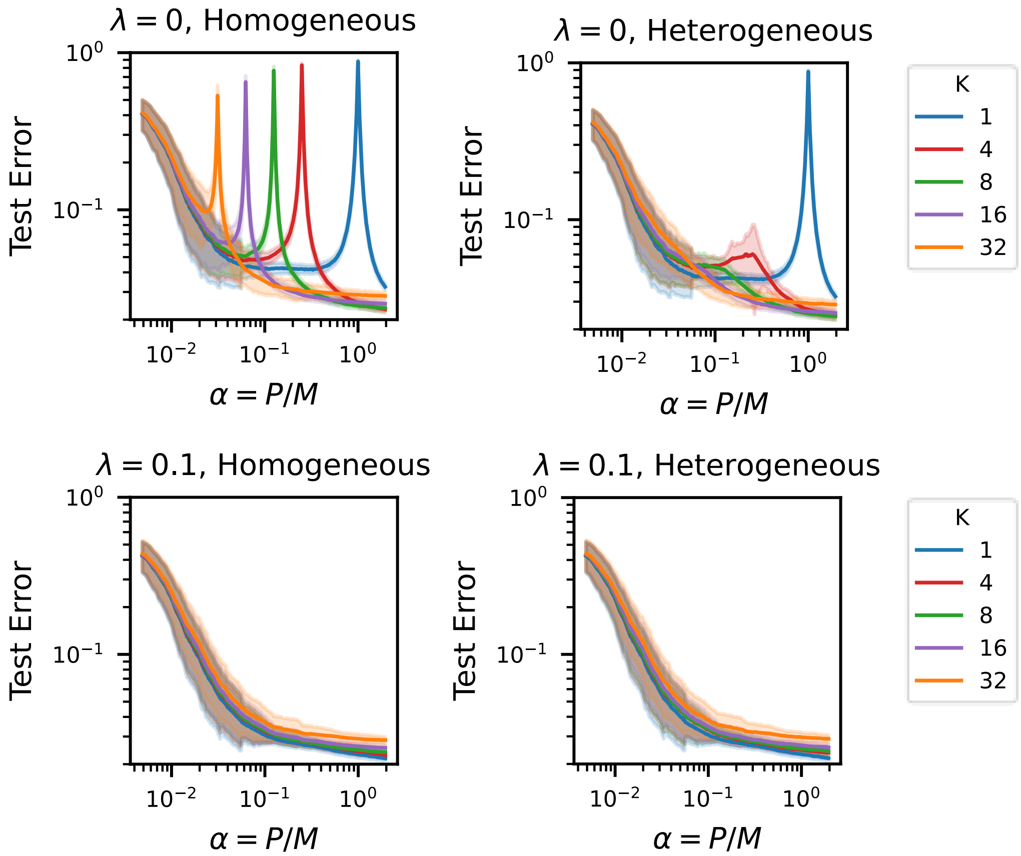

Figure S2: Results of the experiment described in section E.4.2. Learning curves for ResNext-Imagenewoof classification task in linear classifier ensembles. In Homogeneous ensembling (left) all $\nu_{rr} = 1/k$ with k indicated by the legend. In heterogeneous ensembling ν_{rr} are drawn from a Dirichlet distribution as described in section C, with $\sigma = 3/(4k)$. We use regularization $\lambda = 0$ (top) and $\lambda = 0.1$ (bottom). $\lambda = 0, 0.1$. Lines represent an average over 100 trials, shaded regions show standard deviation. We set $\nu_{rr'} = 0$ for $r \neq r'$.

which was found to mitigate double-descent by the parameter sweep in Fig. S1c. In main text Fig. 3e, we plot the mean learning curves over 100 trials. In Fig. S2 we show standard deviation over the 100 trials as shaded error bars. When $\lambda = 0$, heterogeneous ensembling mitigates double-descent, leading to a monotonically decreasing learning curve for sufficiently high k. When $\lambda = 0.1$, homogeneous and heterogeneous ensembles of size k perform similarly. Code used to run these experiments may be found in the folder "DeepNet_HomVHet" in the GitHub repository.

E.4.3 Readout Noise Encourages Ensembling

In the third experiment, we test the effect of a readout noise which is independent across the members of the ensemble on generalization error. We do this by sweeping over the readout noise scale η as defined in sections E.2, E.3. For $\lambda = 0, 0.1$ and $\nu = 0, .1, \ldots, 1$ we compute the learning curves of linear predictors with $k = 1, 2, 3, 4, 5, 10, 15, 20, 25, 30, 100, 200$ and all $\nu_{rr} = 1/k$, averaged over 50 trials. These learning curves are shown in Fig. S3 for both the ResNext-Imagenette and ResNext-Imagewoof task. In Figs. S4a and S5a, we plot the value k^* which minimizes error as pase diagrams in the parameter space of α and η, analogous to the phase diagrams in Fig. 4b. We see that the qualitative shape of these phase diagrams is similar to the equicorrelated model. The differences may be attributed to the nonlinear nature of the classification task. Furthermore, we find

that, in general the optimal k^* tends to be higher with the Imagenette dataset than with the Imagewoof dataset, in agreement with our finding in the equicorrelated regression model that as ρ increases (making the classification task easier), optimal ensemble size tends to increase (Fig. 4b and S6, S7, S8). In Fig S3 we see that there are often a number of k values for which test error is at or near to its lowest. To quantify this, we also plot diagrams of the minimum and maximum values of k that are within an small margin ϵ of the minimum measured error. For the ResNext-Imagenette task, we use $\epsilon = 0.001$ and for ResNext-Imagewoof $\epsilon = 0.01$. We see that there is a wide array of k values which bring error near-to-minimum in practice (Figs S4b,c, S5b,c). We also plot the minimum achieved error, and the difference between minimum errors at $\lambda = 0.1, 0$ (Figs. S4d, S5d). Code used to run these experiments may be found in the folder "DeepNet_PD" in the GitHub repository.

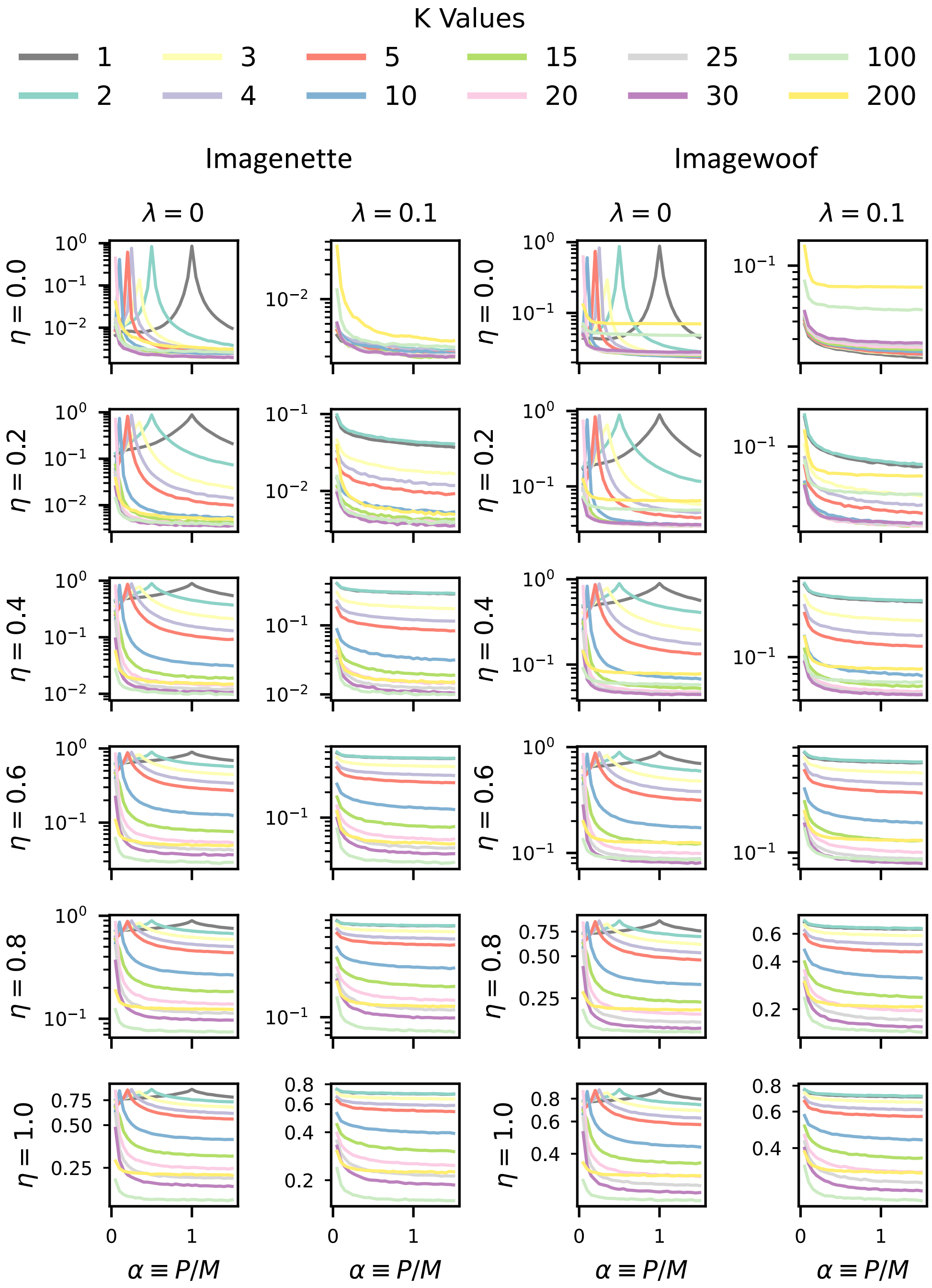

Figure S3: Learning curves for ensembles of linear classifiers with homogeneous subsampling for $\lambda = 0, 0.1$ and readout noise η values indicated in the figure. Results are averaged over 50 trials. Experiments are described in section E.4.3

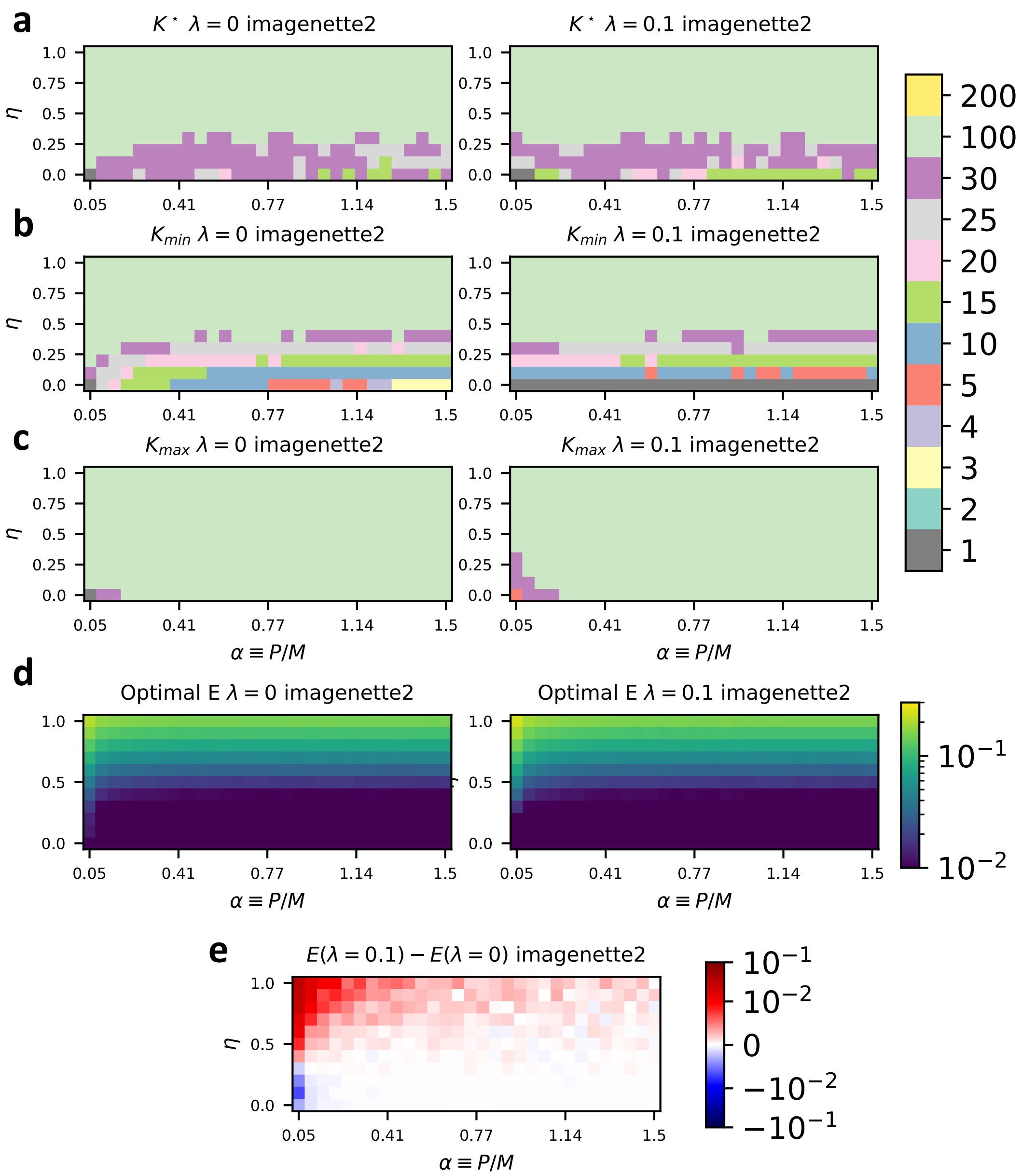

Figure S4: Diagrams described in section E.4.3 for the ResNext-Imagenette experiment. Using learning curves in Fig. S3 (and for additional values of η not shown there) , we plot (a) Optimal k^* in the parameter space of α and η, (b) Minimum value of k for which error is within a tolerance $\epsilon = .001$ of its value for k^*, (b) Maximum value of k for which error is within a tolerance $\epsilon = .001$ of its value for k^*, (d) the value of the minimum error $E(k^*)$, and (e) the difference between this optimal error for $\lambda = 0.1$ and $\lambda = 0$.

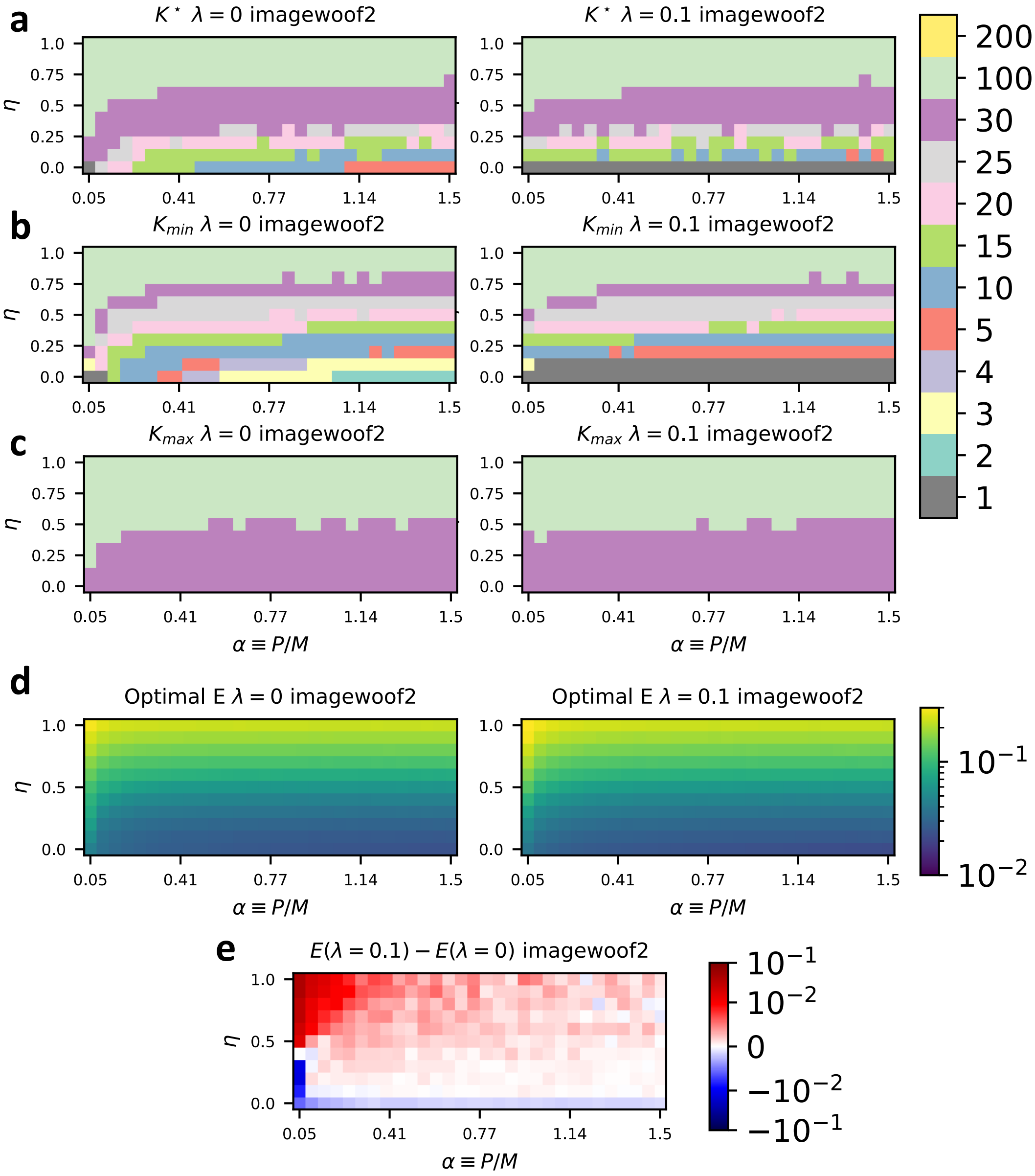

Figure S5: Diagrams described in section E.4.3 for the ResNext-Imagewoof experiment. Using learning curves in Fig. S3 (and for additional values of η not shown there) , we plot (a) Optimal k^* in the parameter space of α and η, (b) Minimum value of k for which error is within a tolerance $\epsilon = .001$ of its value for k^*, (b) Maximum value of k for which error is within a tolerance $\epsilon = .001$ of its value for k^*, (d) the value of the minimum error $E(k^*)$, and (e) the difference between this optimal error for $\lambda = 0.1$ and $\lambda = 0$.

F Generalization error of ensembled linear regression from the replica trick

Here we use the Replica Trick from statistical physics to derive analytical expressions for $E_{rr'}$. We treat the cases where $r = r'$ and $r \neq r'$ separately. Following a statistical mechanics approach, we calculate the average generalization error over a Gibbs measure with inverse temperature β;

$$Z = \int \prod_r d\boldsymbol{w} \exp\left(-\frac{\beta}{2}\sum_r E_t^r - \frac{M\beta}{2}\sum_{r,r'} J_{rr'}E_{rr'}(\boldsymbol{w_r}, \boldsymbol{w}_{r'})\right) \tag{30}$$

$$E_t^r = \sum_{\mu=1}^{P}\left(\frac{1}{\sqrt{N_r}}\boldsymbol{w}_r^\top \boldsymbol{A}_r\bar{\boldsymbol{\psi}}_\mu + \xi_r - y_\mu\right)^2 + \lambda|\boldsymbol{w}_r^2| \tag{31}$$

In the limit where $\beta \to \infty$ the gibbs measure will concentrate around the weight vector $\hat{\boldsymbol{w}}_r$ which minimizes the regularized loss function. The replica trick relies on the following identity:

$$\langle \log(Z[\mathcal{D}])\rangle_{\mathcal{D}} = \lim_{n\to 0}\frac{1}{n}\log\left(\langle Z^n\rangle_{\mathcal{D}}\right) \tag{32}$$

where $\langle\cdot\rangle_{\mathcal{D}}$ represents an average over all quenched disorder in the system. In this case, quenched disorder – the disorder which is fixed prior to and throughout training of the weights – consists of the selected training examples along with their feature noise and label noise: $\mathcal{D} = \{\boldsymbol{\psi}_\mu, \boldsymbol{\sigma}^\mu, \epsilon^\mu\}_{\mu=1}^{P}$. The calculation proceeds by first computing the average of the replicated partition function assuming n is a positive integer. Then, in a non-rigorous but standard step, we analytically extend the result to $n \to 0$.

F.1 Diagonal Terms

We start by calculating E_{rr} for some fixed choice of r. This derivation partially follows section D.3 from [36], with the addition of readout noise and label noise. Noting that the diagonal terms of the generalization error E_{rr} only depend on the learned weights $\boldsymbol{w}_r$, and the loss function separates over the readouts, we may consider the Gibbs measure over only these weights:

$$Z = \int d\boldsymbol{w}_r \exp\left(-\frac{\beta}{2\lambda}E_r^t - \frac{JM\beta}{2}E_{rr}(\boldsymbol{w}_r)\right) \tag{33}$$

$$\begin{aligned}\langle Z^n\rangle_{\mathcal{D}} = &\int \prod_a d\boldsymbol{w}_r^a \mathbb{E}_{\{\boldsymbol{\psi}_\mu, \boldsymbol{\sigma}^\mu, \epsilon^\mu\}} \\ &\exp\left(-\frac{\beta M}{2\lambda}\sum_{\mu,a}\frac{1}{M}\left[\frac{1}{\sqrt{\nu_{rr}}}\boldsymbol{w}_r^{a\top}\boldsymbol{A}_r\left(\boldsymbol{\psi}_\mu + \boldsymbol{\sigma}^\mu\right) - \boldsymbol{w}^{*\top}\boldsymbol{\psi}_\mu - \sqrt{M}(\epsilon^\mu - \xi_r^\mu)\right]^2\right. \\ &\left.-\frac{\beta}{2}\sum_a |\boldsymbol{w}_r^a|^2 - \frac{JM\beta}{2}\sum_a E_{rr}(\boldsymbol{w}^a)\right)\end{aligned} \tag{34}$$

Next we must perform the averages over quenched disorder. We first integrate over $\{\boldsymbol{\psi}_\mu, \boldsymbol{\sigma}^\mu, \xi_r^\mu, \epsilon^\mu\}_{\mu=1}^{P}$. Noting that the scalars

$$h_\mu^{ra} \equiv \frac{1}{\sqrt{M}}\left[\frac{1}{\sqrt{\nu_{rr}}}\boldsymbol{w}_r^{a\top}\boldsymbol{A}_r\left(\boldsymbol{\psi}_\mu + \boldsymbol{\sigma}^\mu\right) - \boldsymbol{w}^{*\top}\boldsymbol{\psi}_\mu - \sqrt{M}(\epsilon^\mu - \xi_r^\mu)\right]$$

are Gaussian random variables (when conditioned on A_r) with mean zero and covariance:

$$\langle h_\mu^{ra} h_\nu^{rb} \rangle = \delta_{\mu\nu} Q_{ab}^{rr} \tag{35}$$

$$Q_{ab}^{rr} = \frac{1}{M}\left[\left(\frac{1}{\sqrt{\nu_{rr}}}\boldsymbol{w}_r^{a\top}\boldsymbol{A}_r - \boldsymbol{w}^{*\top}\right)\boldsymbol{\Sigma}_s\left(\frac{1}{\sqrt{\nu_{rr}}}\boldsymbol{A}_r^\top \boldsymbol{w}_r^b - \boldsymbol{w}^*\right)\right.$$
$$\left. + \frac{1}{\nu_{rr}}\boldsymbol{w}_r^{a\top}\boldsymbol{A}_r\boldsymbol{\Sigma}_0\boldsymbol{A}_r^\top\boldsymbol{w}_r^b + M(\zeta^2+\eta_r^2)\right] \tag{36}$$

To perform this integral we re-write in terms of $\{\boldsymbol{H}_\mu^r\}_{\mu=1}^P$, where

$$\boldsymbol{H}_\mu^r = \begin{bmatrix} h_\mu^{r1} \\ h_\mu^{r2} \\ \vdots \\ h_\mu^{rn} \end{bmatrix} \in \mathbb{R}^n \tag{37}$$

$$\langle Z^n \rangle_{\mathcal{D}} = \int \prod_a d\boldsymbol{w}_r^a \mathbb{E}_{\{\psi_\mu, \boldsymbol{\sigma}^\mu, \boldsymbol{\epsilon}^\mu\}} \exp\left(-\frac{\beta}{2\lambda}\sum_\mu \boldsymbol{H}_\mu^{r\top}\boldsymbol{H}_\mu^r - \frac{\beta}{2}\sum_a |\boldsymbol{w}_r^a|^2 - \frac{JM\beta}{2}\sum_a E_{rr}(\boldsymbol{w}^a)\right) \tag{38}$$

Integrating over the $\boldsymbol{H}_\mu^r$ we get:

$$\langle Z^n \rangle_{\mathcal{D}} = \int \prod_a d\boldsymbol{w}_r^a \exp\left(-\frac{P}{2}\log\det\left(\boldsymbol{I}_n + \frac{\beta}{\lambda}\boldsymbol{Q}^{rr}\right) - \frac{\beta}{2}\sum_a |\boldsymbol{w}_r^a|^2 - \frac{JM\beta}{2}\sum_a E_{rr}(\boldsymbol{w}_r)\right) \tag{39}$$

Next we integrate over $\boldsymbol{Q}^r$ and add constraints. We use the following identity:

$$1 = \prod_{ab'}\int dQ_{ab}^{rr}\delta\left(Q_{ab}^{rr} - \frac{1}{M}\left[\left(\frac{1}{\sqrt{\nu_{rr}}}\boldsymbol{w}_r^{a\top}\boldsymbol{A}_r - \boldsymbol{w}^{*\top}\right)\boldsymbol{\Sigma}_s\left(\frac{1}{\sqrt{\nu_{rr}}}\boldsymbol{A}_r^\top\boldsymbol{w}_r^b - \boldsymbol{w}^*\right)\right.\right.$$
$$\left.\left. + \frac{1}{\nu_{rr}}\boldsymbol{w}_r^{a\top}\boldsymbol{A}_r\boldsymbol{\Sigma}_0\boldsymbol{A}_r^\top\boldsymbol{w}_r^b + M(\zeta^2+\eta_r^2)\right]\right) \tag{40}$$

Using the Fourier representation of the delta function, we get:

$$1 = \prod_{ab}\int \frac{1}{4\pi i/M} dQ_{ab}^{rr} d\hat{Q}_{ab}^{rr} \exp\left(\frac{M}{2}\hat{Q}_{ab}^{rr}\left(Q_{ab}^{rr} - \frac{1}{M}\left[\left(\frac{1}{\sqrt{\nu_{rr}}}\boldsymbol{w}_r^{a\top}\boldsymbol{A}_r - \boldsymbol{w}^{*\top}\right)\boldsymbol{\Sigma}_s\left(\frac{1}{\sqrt{\nu_{rr}}}\boldsymbol{A}_r^\top\boldsymbol{w}_r^b - \boldsymbol{w}^*\right)\right.\right.\right.$$
$$\left.\left.\left. + \frac{1}{\nu_{rr}}\boldsymbol{w}_r^{a\top}\boldsymbol{A}_r\boldsymbol{\Sigma}_0\boldsymbol{A}_r^\top\boldsymbol{w}_r^b + M(\zeta^2+\eta_r^2)\right]\right)\right) \tag{41}$$

Inserting this identity into the replicated partition function and substituting $E_{rr}(\boldsymbol{w}_r^a) = Q_{aa}^{rr} - \zeta^2$ we find:

$$
\langle Z^n \rangle_{\mathcal{D}} \propto
$$

$$
\int \prod_{ab} dQ^{rr}_{ab} d\hat{Q}^{rr}_{ab} \exp\left(-\frac{P}{2} \log\det\left(\boldsymbol{I}_n + \frac{\beta}{\lambda} \boldsymbol{Q}^{rr} \right) + \frac{1}{2} \sum_{ab} M \hat{Q}^{rr}_{ab} Q^{rr}_{ab} - \frac{JM\beta}{2} \sum_{a} (Q^{rr}_{aa} - \zeta^2) \right)
$$

$$
\int \prod_{a} d\boldsymbol{w}^a_r \exp\left(-\frac{\beta}{2} \sum_{a} |\boldsymbol{w}^a_r|^2 - \frac{1}{2} \sum_{ab} \hat{Q}^{rr}_{ab} \left[\left(\frac{1}{\sqrt{\nu_{rr}}} \boldsymbol{w}^{a\top}_r \boldsymbol{A}_r - \boldsymbol{w}^{*\top} \right) \boldsymbol{\Sigma}_s \left(\frac{1}{\sqrt{\nu_{rr}}} \boldsymbol{A}^\top_r \boldsymbol{w}^b_r - \boldsymbol{w}^* \right) \right.\right.
$$

$$
\left.\left. + \frac{1}{\nu_{rr}} \boldsymbol{w}^{a\top}_r \boldsymbol{A}_r \boldsymbol{\Sigma}_0 \boldsymbol{A}^\top_r \boldsymbol{w}^b_r + M(\zeta^2 + \eta^2_r) \right] \right) \tag{42}
$$

In order to perform the Gaussian integral over the $\{\boldsymbol{w}^a_r\}$, we unfold over the replica index a. We first define the following:

$$
\boldsymbol{w}^{\cdot}_r \equiv \begin{bmatrix} \boldsymbol{w}^1_r \\ \vdots \\ \boldsymbol{w}^n_r \end{bmatrix} \tag{43}
$$

$$
T^r \equiv \beta \boldsymbol{I}_n \otimes \boldsymbol{I}_{N_r} + \hat{\boldsymbol{Q}}^{rr} \otimes \left(\frac{1}{\nu_{rr}} \boldsymbol{A}_r (\boldsymbol{\Sigma}_s + \boldsymbol{\Sigma}_0) \boldsymbol{A}^\top_r \right) \tag{44}
$$

$$
V^r \equiv (\hat{\boldsymbol{Q}}^{rr} \otimes \boldsymbol{I}_{N_r})(\boldsymbol{1}_n \otimes \frac{1}{\sqrt{\nu_{rr}}} \boldsymbol{A}_r \boldsymbol{\Sigma}_s \boldsymbol{w}^*) \tag{45}
$$

We then have for the integral over w

$$
\int d\boldsymbol{w}^{\cdot}_r \exp\left(-\frac{1}{2} \boldsymbol{w}^{\cdot\top}_r T^r \boldsymbol{w}^{\cdot}_{r'} + V^{r\top} \boldsymbol{w}^{\cdot}_r \right) \tag{46}
$$

$$
= \exp\left(\frac{1}{2} V^{r\top} (T^r)^{-1} V^r - \frac{1}{2} \log\det(T^r) \right) \tag{47}
$$

We can finally write the replicated partition function as:

$$
\langle Z^n \rangle_{\mathcal{D}} \propto
$$

$$
\int \prod_{ab} dQ^{rr}_{ab} d\hat{Q}^{r}_{ab} \exp\left(-\frac{P}{2} \log\det\left(\boldsymbol{I}_n + \frac{\beta}{\lambda} \boldsymbol{Q}^{rr} \right) + \frac{1}{2} \sum_{ab} M \hat{Q}^{rr}_{ab} Q^{rr}_{ab} - \frac{JM\beta}{2} \sum_{a} (Q^{rr}_{aa} - \zeta^2) \right)
$$

$$
\exp\left(\frac{1}{2} V^{r\top} (T^r)^{-1} V^r - \frac{1}{2} \log\det(T^r) - \frac{1}{2} \sum_{ab} \hat{Q}^{rr}_{ab} (M(\zeta^2 + \eta^2_r) + \boldsymbol{w}^{*\top} \Sigma_s \boldsymbol{w}^*) \right) \tag{48}
$$

We now make the following replica-symmetric ansatz:

$$
Q^{rr}_{ab} = \beta^{-1} q \delta_{ab} + q_0 \tag{49}
$$

$$
\hat{Q}^{rr}_{ab} = \beta \hat{q} \delta_{ab} + \beta^2 \hat{q}_0 \tag{50}
$$

which is well-motivated because the loss function is convex. We will verify that the chosen scalings are self-consistent in the zero-temperature limit where $\beta \to \infty$. We may then rewrite the partition function as follows:

$$\langle Z^n \rangle_{\mathcal{D}} = \exp\left(-\frac{nM}{2}\mathfrak{g}\left[q, q_0, \hat{q}, \hat{q}_0\right]\right) \tag{51}$$

Where the effective action is written:

$$\mathfrak{g}\left[q, q_0, \hat{q}, \hat{q}_0\right] = \alpha\left[\log(1+\frac{q}{\lambda}) + \frac{\beta q_0}{\lambda+q}\right] - (q\hat{q} + \beta q\hat{q}_0 + \beta q_0\hat{q}) + \beta J\left[(\beta^{-1}q + q_0) - \zeta^2\right]$$
$$-\frac{\beta}{\nu_{rr}M}\hat{q}^2 \boldsymbol{w}^{*\top}\boldsymbol{\Sigma}_s\boldsymbol{A}_r^\top\boldsymbol{G}^{-1}\boldsymbol{A}_r\boldsymbol{\Sigma}_s\boldsymbol{w}^* + \frac{1}{M}\left[\log\det(\boldsymbol{G}) + \beta\hat{q}_0\operatorname{tr}[\boldsymbol{G}^{-1}\tilde{\boldsymbol{\Sigma}}]\right] + \beta\hat{q}\left(\zeta^2 + \eta_r^2 + \frac{1}{M}\boldsymbol{w}^{*\top}\boldsymbol{\Sigma}_s\boldsymbol{w}^*\right) \tag{52}$$

Where $\boldsymbol{G} \equiv \boldsymbol{I}_{N_r} + \hat{q}\tilde{\boldsymbol{\Sigma}}$ and $\tilde{\boldsymbol{\Sigma}} \equiv \frac{1}{\nu_{rr}}\boldsymbol{A}_r(\boldsymbol{\Sigma}_s + \boldsymbol{\Sigma}_0)\boldsymbol{A}_r^\top$

To determine the values of the order parameters we set the derivatives of $\mathfrak{g}\left[q, q_0, \hat{q}, \hat{q}_0\right]$, evaluated at zero source ($J = 0$), to zero:

$$\frac{\partial \mathfrak{g}}{\partial q_0} = 0 = \frac{\alpha\beta}{\lambda+q} - \beta\hat{q} \qquad \Rightarrow \hat{q} = \frac{\alpha}{\lambda+q} \tag{53}$$
$$\frac{\partial \mathfrak{g}}{\partial \hat{q}_0} = 0 = -\beta q + \frac{\beta}{M}\operatorname{tr}\left[\boldsymbol{G}^{-1}\tilde{\boldsymbol{\Sigma}}\right] \qquad \Rightarrow q = \frac{1}{M}\operatorname{tr}\left[\boldsymbol{G}^{-1}\tilde{\boldsymbol{\Sigma}}\right] \tag{54}$$
$$\frac{\partial \mathfrak{g}}{\partial q} = 0 = \frac{\alpha}{\lambda+q} - \frac{\alpha q_0\beta}{(\lambda+\beta q)^2} - \hat{q} - \beta\hat{q}_0 \qquad \Rightarrow \hat{q}_0 = -\frac{\alpha q_0}{(\lambda+\beta q)^2} \tag{55}$$

$$\frac{\partial \mathfrak{g}}{\partial \hat{q}} = 0 = -q - \beta q_0 + \beta\zeta^2 + \beta\eta_r^2 + \frac{1}{M}\operatorname{tr}\left[\boldsymbol{G}^{-1}\tilde{\boldsymbol{\Sigma}}\right] - \frac{\beta\hat{q}_0}{M}\operatorname{tr}\left[\left(\boldsymbol{G}^{-1}\tilde{\boldsymbol{\Sigma}}\right)^2\right]$$
$$+\frac{\beta}{M}\boldsymbol{w}^{*\top}\left[\boldsymbol{\Sigma}_s - \frac{2}{\nu_{rr}}\hat{q}\boldsymbol{\Sigma}_s\boldsymbol{A}_r^\top\boldsymbol{G}^{-1}\boldsymbol{A}_r\boldsymbol{\Sigma}_s + \frac{1}{\nu_{rr}}\hat{q}^2\boldsymbol{\Sigma}_s\boldsymbol{A}_r^\top\boldsymbol{G}^{-1}\tilde{\boldsymbol{\Sigma}}\boldsymbol{G}^{-1}\boldsymbol{A}_r\boldsymbol{\Sigma}_s\right]\boldsymbol{w}^* \tag{56}$$
$$\Rightarrow q_0 = \frac{1}{1-\gamma}\left(\zeta^2 + \eta_r^2 + \frac{1}{M}\boldsymbol{w}^{*\top}\left[\boldsymbol{\Sigma}_s - \frac{2}{\nu_{rr}}\hat{q}\boldsymbol{\Sigma}_s\boldsymbol{A}_r^\top\boldsymbol{G}^{-1}\boldsymbol{A}_r\boldsymbol{\Sigma}_s + \frac{1}{\nu_{rr}}\hat{q}^2\boldsymbol{\Sigma}_s\boldsymbol{A}_r^\top\boldsymbol{G}^{-1}\tilde{\boldsymbol{\Sigma}}\boldsymbol{G}^{-1}\boldsymbol{A}_r\boldsymbol{\Sigma}_s\right]\boldsymbol{w}^*\right) \tag{57}$$

where $\gamma \equiv \frac{\alpha}{M\kappa^2}\operatorname{tr}\left[(\boldsymbol{G}^{-1}\tilde{\boldsymbol{\Sigma}})^2\right]$ and $\kappa \equiv \lambda + q$. We retroactively confirm that the chosen β-scalings are correct by noticing that the saddle-point equations permit a solution where all order parameters remain $\mathcal{O}(1)$ as $\beta \to \infty$. The error is given as:

$$E_{rr} = \lim_{\beta\to\infty}\frac{1}{\beta}\frac{\partial}{\partial J}\mathfrak{g}[q, q_0, \hat{q}, \hat{q}_0] = \lim_{\beta\to\infty}\beta^{-1}q + q_0 - \zeta^2 = q_0 - \zeta^2 \tag{58}$$

Where $q, q_0, \hat{q}, \hat{q}_0$ are the solutions to the saddle-point equations 53, 54, 55, 57. Substituting eq. 57 for q_0, we obtain:

$$E_{rr} = \frac{1}{1-\gamma}\frac{1}{M}\boldsymbol{w}^{\star\top}\left[\boldsymbol{\Sigma}_s - \frac{2}{\nu_{rr}}\hat{q}\boldsymbol{\Sigma}_s\boldsymbol{A}_r^\top\boldsymbol{G}^{-1}\boldsymbol{A}_r\boldsymbol{\Sigma}_s + \frac{1}{\nu_{rr}}\hat{q}^2\boldsymbol{\Sigma}_s\boldsymbol{A}_r^\top\boldsymbol{G}^{-1}\tilde{\boldsymbol{\Sigma}}\boldsymbol{G}^{-1}\boldsymbol{A}_r\boldsymbol{\Sigma}_s\right]\boldsymbol{w}^* + \frac{\gamma\zeta^2 + \eta_r^2}{1-\gamma} \tag{59}$$
$$= \frac{1}{1-\gamma}\frac{1}{M}\boldsymbol{w}^{\star\top}\left[\boldsymbol{\Sigma}_s - \frac{1}{\nu_{rr}}\hat{q}\boldsymbol{\Sigma}_s\boldsymbol{A}_r^\top\boldsymbol{G}^{-1}\boldsymbol{A}_r\boldsymbol{\Sigma}_s - \frac{1}{\nu_{rr}}\hat{q}\boldsymbol{\Sigma}_s\boldsymbol{A}_r^\top\boldsymbol{G}^{-2}\boldsymbol{A}_r\boldsymbol{\Sigma}_s\right]\boldsymbol{w}^* + \frac{\gamma\zeta^2 + \eta_r^2}{1-\gamma} \tag{60}$$

F.2 Off-Diagonal Terms

We now calculate $E_{rr'}$ for $r \neq r'$. We now must consider the joint Gibbs Measure over $\boldsymbol{w}_r$ and $\boldsymbol{w}_{r'}$:

$$Z = \int d\boldsymbol{w}_r d\boldsymbol{w}_{r'} \exp\left(-\frac{\beta}{2\lambda}(E_r^t + E_{r'}^t) - \frac{JM\beta}{2} E_{rr'}(\boldsymbol{w}_r, \boldsymbol{w}_{r'})\right) \tag{61}$$

$$\tag{62}$$

$$\langle Z^n \rangle_{\mathcal{D}} = \int \prod_a d\boldsymbol{w}_r^a d\boldsymbol{w}_{r'}^a \mathbb{E}_{\{\psi_\mu, \boldsymbol{\sigma}^\mu, \epsilon^\mu\}} \exp\left(-\frac{\beta M}{2\lambda} \sum_{\mu,a} \frac{1}{M}\left[\left(h_\mu^{ra}\right)^2 + \left(h_\mu^{r'a}\right)^2\right] - \frac{\beta}{2}\sum_a \left[|\boldsymbol{w}_r^a|^2 + |\boldsymbol{w}_{r'}^a|^2\right] - \frac{JM\beta}{2}\sum_a E_{rr'}(\boldsymbol{w}_r^a, \boldsymbol{w}_{r'}^a)\right) \tag{63}$$

Where the h_μ^{ra} are defined as before. Next we must perform the averages over quenched disorder. To do so, we note that the h_μ^{ra} are Gaussian random variables with covariance structure:

$$\langle h_\mu^{ra} h_\nu^{r'b} \rangle = \delta_{\mu\nu} Q_{ab}^{rr'} \tag{64}$$

$$Q_{ab}^{rr'} = \frac{1}{M}\left[\left(\frac{1}{\sqrt{\nu_{rr}}}\boldsymbol{w}_r^{a\top}\boldsymbol{A}_r - \boldsymbol{w}^{*\top}\right)\boldsymbol{\Sigma}_s\left(\frac{1}{\sqrt{\nu_{r'r'}}}\boldsymbol{A}_{r'}^\top \boldsymbol{w}_{r'}^b - \boldsymbol{w}^*\right) + \frac{1}{\sqrt{\nu_{rr}\nu_{r'r'}}}\boldsymbol{w}_r^{a\top}\boldsymbol{A}_r\boldsymbol{\Sigma}_0\boldsymbol{A}_{r'}^\top\boldsymbol{w}_{r'}^b + M\zeta^2\right] \tag{65}$$

To perform this integral we re-write in terms of $\{\boldsymbol{H}_\mu\}_{\mu=1}^P$, where

$$\boldsymbol{H}_\mu = \begin{bmatrix}\boldsymbol{H}_\mu^r \\ \boldsymbol{H}_\mu^{r'}\end{bmatrix} \in \mathbb{R}^{2n} \tag{66}$$

$$\langle Z^n \rangle_{\mathcal{D}} = \int \prod_a d\boldsymbol{w}_r^a d\boldsymbol{w}_{r'}^a \mathbb{E}_{\{\psi_\mu, \boldsymbol{\sigma}^\mu, \epsilon^\mu\}} \exp\left(-\frac{\beta}{2\lambda}\sum_\mu \boldsymbol{H}_\mu^\top \boldsymbol{H}_\mu - \frac{\beta}{2}\sum_a \left[|\boldsymbol{w}_r^a|^2 + |\boldsymbol{w}_{r'}^a|^2\right] - \frac{JM\beta}{2}\sum_a E_{rr'}(\boldsymbol{w}_r^a, \boldsymbol{w}_{r'}^a)\right) \tag{67}$$

Integrating over $\boldsymbol{H}_\mu$ we get:

$$\langle Z^n \rangle_{\mathcal{D}} = \int \prod_a d\boldsymbol{w}_r^a d\boldsymbol{w}_{r'}^a \exp\left(-\frac{P}{2}\log\det\left(\boldsymbol{I}_{2n} + \frac{\beta}{\lambda}\boldsymbol{Q}\right) - \frac{\beta}{2}\sum_a \left[|\boldsymbol{w}_r^a|^2 + |\boldsymbol{w}_{r'}^a|^2\right] - \frac{JM\beta}{2}\sum_a E_{rr}(\boldsymbol{w}_r^a, \boldsymbol{w}_{r'}^a)\right) \tag{68}$$

Where we have defined the matrix $\boldsymbol{Q}$ so that:

$$\boldsymbol{Q} = \begin{bmatrix}\boldsymbol{Q}^{rr} & \boldsymbol{Q}^{rr'} \\ \boldsymbol{Q}^{rr'} & \boldsymbol{Q}^{r'r'}\end{bmatrix} \tag{69}$$

Next we integrate over $\boldsymbol{Q}$ and add constraints. We use the following identity:

$$1 = \prod_{ab} \int dQ_{ab}^{rr'} \delta \left(Q_{ab}^{rr'} - \frac{1}{M} \left[\left(\frac{1}{\sqrt{\nu_{rr}}} \boldsymbol{w}_r^{a\top} \boldsymbol{A}_r - \boldsymbol{w}^{*\top} \right) \boldsymbol{\Sigma}_s \left(\frac{1}{\sqrt{\nu_{r'r'}}} \boldsymbol{A}_{r'}^\top \boldsymbol{w}_{r'}^b - \boldsymbol{w}^* \right) \right. \right.$$
$$\left. \left. + \frac{1}{\sqrt{\nu_{rr}\nu_{r'r'}}} \boldsymbol{w}_r^{a\top} \boldsymbol{A}_r \boldsymbol{\Sigma}_0 \boldsymbol{A}_{r'}^\top \boldsymbol{w}_{r'}^b + M\zeta^2 \right] \right) \tag{70}$$

Using the Fourier representation of the delta function, we get:

$$1 = \prod_{ab} \int \frac{1}{4\pi i/M} dQ_{ab}^{rr'} d\hat{Q}_{ab}^{rr'} \exp \left(\frac{M}{2} \hat{Q}_{ab}^{rr'} \left(Q_{ab}^{rr'} - \frac{1}{M} \left[\left(\frac{1}{\sqrt{\nu_{rr}}} \boldsymbol{w}_r^{a\top} \boldsymbol{A}_r - \boldsymbol{w}^{*\top} \right) \boldsymbol{\Sigma}_s \left(\frac{1}{\sqrt{\nu_{r'r'}}} \boldsymbol{A}_{r'}^\top \boldsymbol{w}_{r'}^b - \boldsymbol{w}^* \right) \right. \right. \right.$$
$$\left. \left. \left. + \frac{1}{\nu_{rr}} \boldsymbol{w}_r^{a\top} \boldsymbol{A}_r \boldsymbol{\Sigma}_0 \boldsymbol{A}_{r'}^\top \boldsymbol{w}_{r'}^b + M\zeta^2 \right] \right) \right) \tag{71}$$

Inserting this identity and the corresponding statements for Q_{ab}^{rr} and $Q_{ab}^{r'r'}$ into the replicated partition function and substituting $E_{rr'}(\boldsymbol{w}^a) = Q_{aa}^{rr'} - \zeta^2$ we find:

$$\langle Z^n \rangle_{\mathcal{D}} \propto \int \prod_{abr_1r_2} dQ_{ab}^{r_1r_2} d\hat{Q}_{ab}^{r_1r_2}$$
$$\exp \left(-\frac{P}{2} \log\det \left(\boldsymbol{I}_{2n} + \frac{\beta}{\lambda} \boldsymbol{Q} \right) + \frac{1}{2} \sum_{abr_1r_2} M \hat{Q}_{ab}^{r_1r_2} Q_{ab}^{r_1r_2} - \frac{JM\beta}{2} \sum_a (Q_{aa}^{rr'} - \zeta^2) \right)$$
$$\int \prod_a d\boldsymbol{w}_r^a d\boldsymbol{w}_{r'}^a \exp \left(-\frac{\beta}{2} \sum_a \left[|\boldsymbol{w}_r^a|^2 + |\boldsymbol{w}_{r'}^a|^2 \right] - \frac{1}{2} \sum_{abr_1r_2} \hat{Q}_{ab}^{r_1r_2} \left[\left(\frac{1}{\sqrt{\nu_{r_1}}} \boldsymbol{w}_{r_1}^{a\top} \boldsymbol{A}_{r_1} - \boldsymbol{w}^{*\top} \right) \boldsymbol{\Sigma}_s \left(\frac{1}{\sqrt{\nu_{r_2}}} \boldsymbol{A}_{r_2}^\top \boldsymbol{w}_{r_2}^b - \boldsymbol{w}^* \right) \right. \right.$$
$$\left. \left. + \frac{1}{\sqrt{\nu_{r_1}\nu_{r_2}}} \boldsymbol{w}_{r_1}^{a\top} \boldsymbol{A}_{r_1} \boldsymbol{\Sigma}_0 \boldsymbol{A}_{r_2}^\top \boldsymbol{w}_{r_2}^b + M\zeta^2 \right] \right) \tag{72}$$

Where sums over r_1 and r_2 run over $\{r, r'\}$.

In order to perform the Gaussian integral over the $\{\boldsymbol{w}_r^a\}$, we unfold in two steps. We first define the following:

$$\boldsymbol{w}_r^{\cdot} \equiv \begin{bmatrix} \boldsymbol{w}_r^1 \\ \vdots \\ \boldsymbol{w}_r^n \end{bmatrix} \tag{73}$$
$$[\hat{\boldsymbol{Q}}^{rr'}]_{ab} \equiv \hat{Q}_{ab}^{rr'} \tag{74}$$
$$\tilde{\boldsymbol{\Sigma}}_{rr'} \equiv \frac{1}{\sqrt{\nu_{rr}\nu_{r'r'}}} \boldsymbol{A}_r [\boldsymbol{\Sigma}_s + \boldsymbol{\Sigma}_0] \boldsymbol{A}_{r'}^\top \tag{75}$$
$$T^{rr'} \equiv \beta \delta_{rr'} \boldsymbol{I}_n \otimes \boldsymbol{I}_{N_r} + \hat{\boldsymbol{Q}}^{rr'} \otimes \tilde{\boldsymbol{\Sigma}}_{rr'} \tag{76}$$

Unfolding over the replica indices, we then get:

$$
\begin{aligned}
&\langle Z^n\rangle_{\mathcal{D}} \propto \int \prod_{abr_1r_2} dQ_{ab}^{r_1r_2} d\hat{Q}_{ab}^{r_1r_2} \\
&\exp\left(-\frac{P}{2}\log\det\left(\boldsymbol{I}_{2n}+\frac{\beta}{\lambda}\boldsymbol{Q}\right)+\frac{1}{2}\sum_{abr_1r_2} M\hat{Q}_{ab}^{r_1r_2}Q_{ab}^{r_1r_2}-\frac{JM\beta}{2}\sum_a (Q_{aa}^{rr'}-\zeta^2)\right) \\
&\exp\left(-\frac{1}{2}\sum_{abr_1r_2}\hat{Q}_{ab}^{r_1r_2}\left(\boldsymbol{w}^{*\top}\boldsymbol{\Sigma}_s\boldsymbol{w}^*+M\zeta^2\right)\right) \\
&\int d\boldsymbol{w}_r^{\cdot} d\boldsymbol{w}_{r'}^{\cdot}\exp\left(-\frac{1}{2}\sum_{r_1r_2}\boldsymbol{w}_{r_1}^{\cdot\top}T^{r_1r_2}\boldsymbol{w}_{r_2}+\sum_{r_1r_2}\left[(\hat{\boldsymbol{Q}}^{r_1r_2}\otimes\boldsymbol{I}_{N_{r_1}})(\mathbf{1}_n\otimes\frac{1}{\sqrt{\nu_{r_1}}}\boldsymbol{A}_{r_1}\boldsymbol{\Sigma}_s\boldsymbol{w}^*)\right]^\top \boldsymbol{w}_{r_1}\right)
\end{aligned} \tag{77}
$$

Note that the dimensionality of $T^{r_1r_2}$ varies for different choices of r_1 and r_2. Next, we unfold over the two readouts:

$$
\boldsymbol{w} \equiv \begin{bmatrix}\boldsymbol{w}_r^{\cdot}\\ \boldsymbol{w}_{r'}^{\cdot}\end{bmatrix} \tag{78}
$$

$$
T \equiv \begin{bmatrix}T^{rr} & T^{rr'}\\ T^{r'r} & T^{r'r'}\end{bmatrix} \tag{79}
$$

$$
V \equiv \begin{bmatrix}\left((\hat{\boldsymbol{Q}}^{rr}+\hat{\boldsymbol{Q}}^{rr'})\otimes\boldsymbol{I}_{N_r}\right)\left(\mathbf{1}_n\otimes\frac{1}{\sqrt{\nu_{rr}}}\boldsymbol{A}_r\boldsymbol{\Sigma}_s\boldsymbol{w}^*\right)\\ \left((\hat{\boldsymbol{Q}}^{r'r'}+\hat{\boldsymbol{Q}}^{r'r})\otimes\boldsymbol{I}_{N_{r'}}\right)\left(\mathbf{1}_n\otimes\frac{1}{\sqrt{\nu_{r'r'}}}\boldsymbol{A}_{r'}\boldsymbol{\Sigma}_s\boldsymbol{w}^*\right)\end{bmatrix} \tag{80}
$$

The integral over w then becomes:

$$
\int d\boldsymbol{w}\exp\left(-\frac{1}{2}\boldsymbol{w}^\top T\boldsymbol{w}+V^\top\boldsymbol{w}\right)\propto\exp\left(\frac{1}{2}V^\top T^{-1}V-\frac{1}{2}\log\det T\right) \tag{81}
$$

We are now ready to make a replica-symmetric ansatz. The order parameter that we wish to constrain is $Q_{ab}^{rr'}$. Overlaps go between the weights from different replicas of the system as well as different readouts. The scale of the overlap between two measurements depends on their overlap with each other and with the principal components of the data distribution. An ansatz which is replica-symmetric but makes no assumptions about the overlaps between different measurements is as follows:

$$
Q_{ab}^{r_1r_2} = \beta^{-1}q^{r_1r_2}\delta_{ab}+Q^{r_1r_2} \tag{82}
$$

$$
\hat{Q}_{ab}^{r_1r_2} = \beta\hat{q}^{r_1r_2}\delta_{ab}+\beta^2\hat{Q}^{r_1r_2} \tag{83}
$$

Next step is to plug the RS ansatz into the free energy and simplify. To make calculations more transparent, we re-label the paramters in the RS ansatz as follows:

$$
\boldsymbol{Q}^{rr} = \beta^{-1}q\boldsymbol{I}+Q\mathbf{1}\mathbf{1}^\top \tag{84}
$$

$$
\boldsymbol{Q}^{r'r'} = \beta^{-1}r\boldsymbol{I}+R\mathbf{1}\mathbf{1}^\top \tag{85}
$$

$$
\boldsymbol{Q}^{rr'} = \beta^{-1}c\boldsymbol{I}+C\mathbf{1}\mathbf{1}^\top \tag{86}
$$

$$
\hat{\boldsymbol{Q}}^{rr} = \beta\hat{q}\boldsymbol{I}+\beta^2\hat{Q}\mathbf{1}\mathbf{1}^\top \tag{87}
$$

$$
\hat{\boldsymbol{Q}}^{r'r'} = \beta\hat{r}\boldsymbol{I}+\beta^2\hat{R}\mathbf{1}\mathbf{1}^\top \tag{88}
$$

$$
\hat{\boldsymbol{Q}}^{rr'} = \beta\hat{c}\boldsymbol{I}+\beta^2\hat{C}\mathbf{1}\mathbf{1}^\top \tag{89}
$$

In order to simplify $\log\det(\lambda \boldsymbol{I}_{2n} + \beta \boldsymbol{Q})$, we note that this is a symmetric 2-by-2-block matrix, where each block commutes with all other blocks. We may then use [53]'s result to simplify.

$$\log\det(\lambda \boldsymbol{I}_{2n} + \beta \boldsymbol{Q}) = n\left[\log\left((\lambda+q)(\lambda+r)-c^2\right) + \beta\frac{(\lambda+q)R+(\lambda+r)Q-2cC}{(\lambda+q)(\lambda+r)-c^2}\right] + \mathcal{O}(n^2) \tag{90}$$

$$\sum_{abr_1r_2} \hat{\boldsymbol{Q}}_{ab}^{r_1r_2}\boldsymbol{Q}_{ab}^{r_1r_2} = n\left[\left(q\hat{q}+\beta\hat{q}Q+\beta q\hat{Q}\right)+\left(r\hat{r}+\beta\hat{r}R+\beta r\hat{R}\right)+2\left(c\hat{c}+\beta\hat{c}C+\beta c\hat{C}\right)\right]+\mathcal{O}(n^2) \tag{91}$$

$$\sum_a\left(\boldsymbol{Q}_{aa}^{rr'}-\zeta^2\right) = n\left[\frac{1}{\beta}c + C - \zeta^2\right]+\mathcal{O}(n^2) \tag{92}$$

$$\sum_{abr_1r_2}\hat{\boldsymbol{Q}}_{ab}^{r_1r_2} = \beta n\left[\hat{q}+\hat{r}+2\hat{c}\right]+\mathcal{O}(n^2) \tag{93}$$

$$\log\det(T) = n\left[\log(\beta)+\log\det\begin{bmatrix}\boldsymbol{G}_{rr} & \boldsymbol{G}_{rr'}\\ \boldsymbol{G}_{r'r} & \boldsymbol{G}_{r'r'}\end{bmatrix}+\beta\,\mathrm{tr}\left(\begin{bmatrix}\boldsymbol{G}_{rr} & \boldsymbol{G}_{rr'}\\ \boldsymbol{G}_{r'r} & \boldsymbol{G}_{r'r'}\end{bmatrix}^{-1}\begin{bmatrix}\hat{Q}\tilde{\boldsymbol{\Sigma}}_{rr} & \hat{C}\tilde{\boldsymbol{\Sigma}}_{rr'}\\ \hat{C}\tilde{\boldsymbol{\Sigma}}_{r'r} & \hat{R}\tilde{\boldsymbol{\Sigma}}_{r'r'}\end{bmatrix}\right)\right]+\mathcal{O}(n^2) \tag{94}$$

where $\boldsymbol{G}_{rr} = \boldsymbol{I}_{N_r}+\hat{q}\tilde{\boldsymbol{\Sigma}}_{rr} \quad \boldsymbol{G}_{r'r'} = \boldsymbol{I}_{N_{r'}}+\hat{r}\tilde{\boldsymbol{\Sigma}}_{r'r'} \quad \boldsymbol{G}_{rr'} = \hat{c}\tilde{\boldsymbol{\Sigma}}_{rr'} \quad \boldsymbol{G}_{r'r} = \hat{c}\tilde{\boldsymbol{\Sigma}}_{r'r}$ (95)

$$V^\top T^{-1}V = n\beta\boldsymbol{w}^{*\top}\begin{bmatrix}\frac{1}{\sqrt{\nu_{rr}}}(\hat{q}+\hat{c})\boldsymbol{A}_r\boldsymbol{\Sigma}_s\\ \frac{1}{\sqrt{\nu_{r'r'}}}(\hat{r}+\hat{c})\boldsymbol{A}_{r'}\boldsymbol{\Sigma}_s\end{bmatrix}^\top\begin{bmatrix}\boldsymbol{G}_{rr} & \boldsymbol{G}_{rr'}\\ \boldsymbol{G}_{r'r} & \boldsymbol{G}_{r'r'}\end{bmatrix}^{-1}\begin{bmatrix}\frac{1}{\sqrt{\nu_{rr}}}(\hat{q}+\hat{c})\boldsymbol{A}_r\boldsymbol{\Sigma}_s\\ \frac{1}{\sqrt{\nu_{r'r'}}}(\hat{r}+\hat{c})\boldsymbol{A}_{r'}\boldsymbol{\Sigma}_s\end{bmatrix}\boldsymbol{w}^*+\mathcal{O}(n^2) \tag{96}$$

Collecting these terms, we may write the replicated partition function as follows:

$$\langle Z^n\rangle_{\mathcal{D}} = \exp\left(-\frac{nM}{2}\mathfrak{g}\left[q,Q,r,R,c,C,\hat{q},\hat{Q},\hat{r},\hat{R},\hat{c},\hat{C}\right]\right) \tag{97}$$

Where the free energy is written:

$$\mathfrak{g}\left[q,Q,r,R,c,C,\hat{q},\hat{Q},\hat{r},\hat{R},\hat{c},\hat{C}\right] = \tag{98}$$

$$\alpha\left[\log\left((\lambda+q)(\lambda+r)-c^2\right)+\beta\frac{(\lambda+q)R+(\lambda+r)Q-2cC}{(\lambda+q)(\lambda+r)-c^2}\right] \tag{99}$$

$$-\left[\left(q\hat{q}+\beta\hat{q}Q+\beta q\hat{Q}\right)+\left(r\hat{r}+\beta\hat{r}R+\beta r\hat{R}\right)+2\left(c\hat{c}+\beta\hat{c}C+\beta c\hat{C}\right)\right] \tag{100}$$

$$+J(c+\beta C-\beta\zeta^2) \tag{101}$$

$$+\beta\left[\hat{q}+\hat{r}+2\hat{c}\right]\left(\frac{1}{M}\boldsymbol{w}^{*\top}\Sigma\boldsymbol{w}^*+\zeta^2\right) \tag{102}$$

$$-\frac{1}{M}\beta\boldsymbol{w}^{*\top}\begin{bmatrix}\frac{1}{\sqrt{\nu_{rr}}}(\hat{q}+\hat{c})\boldsymbol{A}_r\boldsymbol{\Sigma}_s\\ \frac{1}{\sqrt{\nu_{r'r'}}}(\hat{r}+\hat{c})\boldsymbol{A}_{r'}\boldsymbol{\Sigma}_s\end{bmatrix}^\top\boldsymbol{G}^{-1}\begin{bmatrix}\frac{1}{\sqrt{\nu_{rr}}}(\hat{q}+\hat{c})\boldsymbol{A}_r\boldsymbol{\Sigma}_s\\ \frac{1}{\sqrt{\nu_{r'r'}}}(\hat{r}+\hat{c})\boldsymbol{A}_{r'}\boldsymbol{\Sigma}_s\end{bmatrix}\boldsymbol{w}^* \tag{103}$$

$$+\frac{1}{M}\left[\log(\beta)+\log\det\boldsymbol{G}+\beta\,\mathrm{tr}\left(\boldsymbol{G}^{-1}\begin{bmatrix}\hat{Q}\tilde{\boldsymbol{\Sigma}}_{rr} & \hat{C}\tilde{\boldsymbol{\Sigma}}_{rr'}\\ \hat{C}\tilde{\boldsymbol{\Sigma}}_{r'r} & \hat{R}\tilde{\boldsymbol{\Sigma}}_{r'r'}\end{bmatrix}\right)\right] \tag{104}$$

where we have defined $\boldsymbol{G} \equiv \begin{bmatrix} \boldsymbol{G}_{rr} & \boldsymbol{G}_{rr'} \\ \boldsymbol{G}_{r'r} & \boldsymbol{G}_{r'r'} \end{bmatrix}$

The saddle-point equations for the replica-diagonal order parameters are:

$$\frac{\partial \mathfrak{g}}{\partial Q} = 0 = \beta \frac{\alpha(\lambda + r)}{(\lambda + q)(\lambda + r) - c^2} - \beta \hat{q} \tag{105}$$
$$\frac{\partial \mathfrak{g}}{\partial \hat{Q}} = 0 = -\beta q + \beta \frac{1}{M} \operatorname{tr}\left(\boldsymbol{G}^{-1} \begin{bmatrix} \tilde{\boldsymbol{\Sigma}}_{rr} & \mathbf{0} \\ \mathbf{0} & \mathbf{0} \end{bmatrix} \right) \tag{106}$$
$$\frac{\partial \mathfrak{g}}{\partial R} = 0 = \beta \frac{\alpha(\lambda + q)}{(\lambda + q)(\lambda + r) - c^2} - \beta \hat{r} \tag{107}$$
$$\frac{\partial \mathfrak{g}}{\partial \hat{R}} = 0 = -\beta r + \beta \frac{1}{M} \operatorname{tr}\left(\boldsymbol{G}^{-1} \begin{bmatrix} \mathbf{0} & \mathbf{0} \\ \mathbf{0} & \tilde{\boldsymbol{\Sigma}}_{r'r'} \end{bmatrix} \right) \tag{108}$$
$$\frac{\partial \mathfrak{g}}{\partial C} = 0 = -\beta \frac{2\alpha c}{(\lambda + q)(\lambda + r) - c^2} - 2\beta \hat{c} + \beta J \tag{109}$$
$$\frac{\partial \mathfrak{g}}{\partial \hat{C}} = 0 = -2\beta c + \beta \frac{1}{M} \operatorname{tr}\left(\boldsymbol{G}^{-1} \begin{bmatrix} \mathbf{0} & \tilde{\boldsymbol{\Sigma}}_{rr'} \\ \tilde{\boldsymbol{\Sigma}}_{r'r} & \mathbf{0} \end{bmatrix} \right) \tag{110}$$

Note that when $J = 0$, the saddle point equations 109, 110 are solved by setting $c = \hat{c} = 0$, and in this case the remaining saddle-point equations decouple over the readouts (as expected for independently trained ensemble members) giving: For readout r:

$$0 = \frac{\alpha}{(\lambda + q)} - \hat{q} \tag{111}$$
$$0 = -q + \frac{1}{M} \operatorname{tr}\left(\boldsymbol{G}_{rr}^{-1} \tilde{\boldsymbol{\Sigma}}_{rr} \right) \tag{112}$$

and for readout r':

$$0 = \frac{\alpha}{(\lambda + r)} - \hat{r} \tag{113}$$
$$0 = -r + \frac{1}{M} \operatorname{tr}\left(\boldsymbol{G}_{r'r'}^{-1} \tilde{\boldsymbol{\Sigma}}_{r'r'} \right) \tag{114}$$

These are equivalent to the saddle-point equations for a single readout given in equation 53, 54 as expected for independently trained readouts. It is physically sensible that $c = 0$ when $J = 0$, because at zero source, there is no term in the replicated system energy function which would distinguish the overlap between two readouts from the same replica from the overlap between two readouts in separate replicas (we expect that the total overlap between readouts is non-zero, as we may still have $C > 0$).

The saddle-point equations obtained by setting the derivatives $\frac{\partial \mathfrak{g}}{\partial q} = \frac{\partial \mathfrak{g}}{\partial \hat{q}} = \frac{\partial \mathfrak{g}}{\partial r} = \frac{\partial \mathfrak{g}}{\partial \hat{r}} = 0$ will similarly decouple to recover two copies of the diagonal case 57 55. We will not re-write the expressions here as they are not necessary to determine the off-diagonal error term $E_{rr'}$.

The remaining saddle-point equations are obtained by setting $\frac{\partial \mathfrak{g}}{\partial c} = \frac{\partial \mathfrak{g}}{\partial \hat{c}} = 0$

$$\left. \frac{\partial \mathfrak{g}}{\partial c} \right|_{c=\hat{c}=J=0} = -\frac{2\alpha\beta C}{(\lambda + q)(\lambda + r)} - 2\beta \hat{C} \quad \Rightarrow \quad \hat{C} = -\frac{\alpha C}{(\lambda + q)(\lambda + r)} \tag{115}$$

$$
\begin{aligned}
\left.\frac{\partial \mathfrak{g}}{\partial \hat{c}}\right|_{c=\hat{c}=J=0} = 0 = & -2\beta C + 2\beta\left(\frac{1}{M}\boldsymbol{w}^{*\top}\boldsymbol{\Sigma}_s\boldsymbol{w}^* + \zeta^2\right) \\
& - \frac{2\beta}{M}\boldsymbol{w}^{*\top}\boldsymbol{\Sigma}_s\left[\frac{1}{\nu_{rr}}\hat{q}\boldsymbol{A}_r^\top\boldsymbol{G}_{rr}^{-1}\boldsymbol{A}_r + \frac{1}{\nu_{r'r'}}\hat{r}\boldsymbol{A}_{r'}^\top\boldsymbol{G}_{r'r'}^{-1}\boldsymbol{A}_{r'}\right]\boldsymbol{\Sigma}_s\boldsymbol{w}^* \\
& + \frac{2\beta\hat{q}\hat{r}}{M}\frac{1}{\sqrt{\nu_{rr}\nu_{r'r'}}}\boldsymbol{w}^{*\top}\boldsymbol{\Sigma}_s\boldsymbol{A}_r^\top\boldsymbol{G}_{rr}^{-1}\tilde{\boldsymbol{\Sigma}}_{rr'}\boldsymbol{G}_{r'r'}^{-1}\boldsymbol{A}_{r'}\boldsymbol{\Sigma}_s\boldsymbol{w}^* \\
& - \frac{2\hat{C}\beta}{M}\operatorname{tr}\left[\boldsymbol{G}_{rr}^{-1}\tilde{\boldsymbol{\Sigma}}_{rr'}\boldsymbol{G}_{r'r'}^{-1}\tilde{\boldsymbol{\Sigma}}_{r'r}\right]
\end{aligned} \tag{116}
$$

Solving equations 115 and 116 for C, we obtain:

$$
\begin{aligned}
C = & \frac{1}{1-\gamma}\zeta^2 + \frac{1}{1-\gamma}\left(\frac{1}{M}\boldsymbol{w}^{*\top}\boldsymbol{\Sigma}_s\boldsymbol{w}^*\right) \\
& - \frac{1}{M(1-\gamma)}\boldsymbol{w}^{*\top}\boldsymbol{\Sigma}_s\left[\frac{1}{\nu_{rr}}\hat{q}\boldsymbol{A}_r^\top\boldsymbol{G}_{rr}^{-1}\boldsymbol{A}_r + \frac{1}{\nu_{r'r'}}\hat{r}\boldsymbol{A}_{r'}^\top\boldsymbol{G}_{r'r'}^{-1}\boldsymbol{A}_{r'}\right]\boldsymbol{\Sigma}_s\boldsymbol{w}^* \\
& + \frac{1}{M(1-\gamma)}\hat{q}\hat{r}\frac{1}{\sqrt{\nu_{rr}\nu_{r'r'}}}\boldsymbol{w}^{*\top}\boldsymbol{\Sigma}_s\boldsymbol{A}_r^\top\boldsymbol{G}_{rr}^{-1}\tilde{\boldsymbol{\Sigma}}_{rr'}\boldsymbol{G}_{r'r'}^{-1}\boldsymbol{A}_{r'}\boldsymbol{\Sigma}_s\boldsymbol{w}^*
\end{aligned} \tag{117}
$$

$$
\text{where} \quad \gamma \equiv \frac{\alpha}{(\lambda+q)(\lambda+r)}\operatorname{tr}\left[\boldsymbol{G}_{rr}^{-1}\tilde{\boldsymbol{\Sigma}}_{rr'}\boldsymbol{G}_{r'r'}^{-1}\tilde{\boldsymbol{\Sigma}}_{r'r}\right] \tag{118}
$$

We can obtain the generalization error as:

$$
E_{rr'} = \lim_{\beta\to\infty}\frac{1}{\beta}\frac{\partial}{\partial J}\mathfrak{g}\left[q,Q,r,R,c,C,\hat{q},\hat{Q},\hat{r},\hat{R},\hat{c},\hat{C}\right] = \lim_{\beta\to\infty}\frac{1}{\beta}(c+\beta C-\beta\zeta^2) = C-\zeta^2 \tag{119}
$$

We may then simplify the expression for the $E_{rr'}$ error as follows:

$$
\begin{aligned}
E_{rr'} = & \frac{\gamma}{1-\gamma}\zeta^2 + \frac{1}{1-\gamma}\left(\frac{1}{M}\boldsymbol{w}^{*\top}\boldsymbol{\Sigma}_s\boldsymbol{w}^*\right) \\
& - \frac{1}{M(1-\gamma)}\boldsymbol{w}^{*\top}\boldsymbol{\Sigma}_s\left[\frac{1}{\nu_{rr}}\hat{q}\boldsymbol{A}_r^\top\boldsymbol{G}_{rr}^{-1}\boldsymbol{A}_r + \frac{1}{\nu_{r'r'}}\hat{r}\boldsymbol{A}_{r'}^\top\boldsymbol{G}_{r'r'}^{-1}\boldsymbol{A}_{r'}\right]\boldsymbol{\Sigma}_s\boldsymbol{w}^* \\
& + \frac{1}{M(1-\gamma)}\hat{q}\hat{r}\frac{1}{\sqrt{\nu_{rr}\nu_{r'r'}}}\boldsymbol{w}^{*\top}\boldsymbol{\Sigma}_s\boldsymbol{A}_r^\top\boldsymbol{G}_{rr}^{-1}\tilde{\boldsymbol{\Sigma}}_{rr'}\boldsymbol{G}_{r'r'}^{-1}\boldsymbol{A}_{r'}\boldsymbol{\Sigma}_s\boldsymbol{w}^*
\end{aligned} \tag{120}
$$

Re-labeling the order parameters: $\hat{q} \to \hat{q}_r$, $\hat{r} \to \hat{q}_{r'}$, $\gamma \to \gamma_{rr'}$ and $\boldsymbol{G}_{rr} \to \boldsymbol{G}_r$, we obtain the result given in the main text.

G Derivation of Proposition 1 from [31]

In the case where the data and noise covariance matrices $\boldsymbol{\Sigma}_s$ and $\boldsymbol{\Sigma}_0$ have bounded spectra, our main result may be derived using Theorem 4.1 from Loureiro et. al. [31], with a few additional arguments to incorporate a readout noise which varies across ensemble members, and to allow for the presence of label noise in the training set but not at test time. Rather than reproducing their very lengthy statements here, we direct the reader to theorem 4.1 and corollary 4.2 in [31].

G.1 Altered Expectations at Evaluation

In [31], labels are generated at both training and test time through the same statistical process $y \sim P_y^0(y|\nu)$ where $\nu = \frac{\langle \boldsymbol{x}|\boldsymbol{\theta}\rangle}{\sqrt{d}}$. However, their results may be easily extended to the case where data are generated through a different statistical process for the training set and at evaluation. This will allow the application of their results to the case where label noise is present during training but not at evaluation as studied in this work. We therefore introduce separate distributions of the labels y at training and evaluation. During training, we still have $y \sim P_y^0(y|\nu)$. At evaluation, we put $y \sim P_y^g(y|\nu)$. This leads to the updated formula:

$$\mathbb{E}_{(y,\boldsymbol{x})}\left[\varphi\left(y, \frac{\langle\langle \hat{\boldsymbol{W}} \mid \boldsymbol{U}\rangle\rangle}{\sqrt{p}}\right)\right] \xrightarrow{\text{P}} \mathbb{E}_{(\nu,\boldsymbol{\mu})}\left[\int dy P_y^g(y|\nu)\varphi(y,\boldsymbol{\mu})\right] \tag{121}$$

when the expectation is over data-label pairs $(y, \boldsymbol{x})$ at *evaluation*.

G.2 Rigorous Proof of Proposition 1

We now restate the the problem setup of our main theorem using notation consistent with [31]. We study generalization error in an ensemble of estimators $\{\hat{\boldsymbol{w}}_k\}$, $k = 1, \ldots, K$. We say $\hat{\boldsymbol{w}}_k \in \mathbb{R}^p$ for all $k = 1, \ldots, K$. The weights are trained independently such that each minimizes a ridge loss function:

$$\begin{aligned}\hat{\boldsymbol{w}}_k &= \arg\min_{\boldsymbol{w}} \sum_{\mu=1}^{n}\left(y^\mu - \frac{1}{\sqrt{N_k}}\boldsymbol{u}_k^\top \boldsymbol{w} - \xi_k^\mu\right)^2 + \lambda_k|\boldsymbol{w}|^2 \qquad k = 1, \ldots, K \\ &= \arg\min_{\boldsymbol{w}} \sum_{\mu=1}^{n}\left(y^\mu - \frac{1}{\sqrt{p}}\boldsymbol{u}_k^\top\left(\frac{1}{\sqrt{\nu_{kk}}}\boldsymbol{w}\right) - \xi_k^\mu\right)^2 + \nu_{kk}\lambda_k|\frac{1}{\sqrt{\nu_{kk}}}\boldsymbol{w}|^2\end{aligned} \tag{122}$$

So that our results will correspond to the results of [31] after re-scaling the regularizations $\lambda_k \to \nu_{kk}\lambda_k$. The training labels are drawn as $y^\mu \sim P_y^0(y|\frac{1}{\sqrt{p}}\boldsymbol{\theta}^\top\boldsymbol{x}^\mu)$ where $P_y^0(y|x) = \mathcal{N}(x, \zeta^2)$, $\xi_k^\mu \sim \mathcal{N}(0, \eta_r^2)$ and θ represent the ground truth weights. For $k = 1, \ldots, k$ we have a "measurement matrix" $\boldsymbol{A}_k \in \mathbb{R}^{N_k \times d}$, and we may set $d = p \geq \max_k N_k$ (so that $\gamma = \frac{d}{p} = 1$). The feature vectors $\boldsymbol{u}_k(x)$ are then drawn as

$$\boldsymbol{u}_k(\boldsymbol{x}) = \begin{bmatrix}\boldsymbol{A}_k(\boldsymbol{x}+\boldsymbol{\sigma}) \\ \boldsymbol{0}_{(p-N_k)}\end{bmatrix} = \bar{\boldsymbol{A}}_k(\boldsymbol{x}+\boldsymbol{\sigma})$$

where $\boldsymbol{x} \sim \mathcal{N}(0, \boldsymbol{\Sigma}_s)$ and $\boldsymbol{\sigma} \sim \mathcal{N}(0, \boldsymbol{\Sigma}_0)$. For convenience, we have defined the auxilary matrices

$$\bar{\boldsymbol{A}}_k \equiv \begin{bmatrix}\boldsymbol{A}_k \\ \boldsymbol{0}_{(p-N_k)\times p}\end{bmatrix} \in \mathbb{R}^{p\times p}$$

By constructing the feature vectors as p-dimensional vectors with only N_k non-zero components, we may apply the results of [31] while preserving structural heterogeneity (we may have $N_k \neq N_{k'}$ for $k \neq k'$) as is present in our main result. Because $[\boldsymbol{u}_k]_i = 0$ for all $i > N_k$, these auxiliary dimensions will not affect model predictions or generalization error. We then have $\mu_k = \frac{1}{\sqrt{p}}\boldsymbol{u}_k^\top\hat{\boldsymbol{w}}_k$, and labels are generated at evaluation according to :

$$y \sim P_y^g(y|\frac{1}{K}\sum_k \mu_k) \text{ where } P_y^g(y|x) = \mathcal{N}(x, \frac{1}{K^2}\sum_k \eta_k^2) \tag{123}$$

The generalization error may then be decomposed as $E_g = \frac{1}{K^2}\sum_{k,k'} E_{kk'}$ where $E_{kk'} = \mathbb{E}_{(y,\boldsymbol{x})}\left[(y-\mu_k)(y-\mu_{k'})\right]$. We will apply eq. 121 separately to calculate $E_{rr'}$ in the cases where $r \neq r'$ and $r = r'$.

G.2.1 Off-Diagonal Terms

Eq. 121 cannot be used to calculate $E_{rr'}$ directly in the case where $r \neq r'$ due to the presence of noises ξ_k^μ which vary over elements of the ensemble (indexed by k) in the loss function (eq. 122). We may argue, however, that the presence of readout noise noise has no effect on the expected value of $E_{rr'}$ when $r \neq r'$, then apply eq. 121 with $\eta_k = \eta_{k'} = 0$. To see this, we examine the analytical form of the minimizer of the loss function (eq, 122):

$$\hat{\boldsymbol{w}}_k = \boldsymbol{U}_k^\top \left(\boldsymbol{U}_k \boldsymbol{U}_k^\top + \lambda_k \boldsymbol{I}\right)^{-1} (\boldsymbol{y} - \boldsymbol{\xi}_k) \tag{124}$$

where we have defined the design matrices $[\boldsymbol{U}_k]_{i\mu} = \frac{1}{\sqrt{p}}[\boldsymbol{u}_k(\boldsymbol{x}^\mu)]_i$ and the vectors $[\boldsymbol{y}]_\mu = y^\mu$ and $[\boldsymbol{\xi}_k]_\mu = \xi_k^\mu$. We then have

$$\begin{aligned} E_{kk'} &= \left(y - \frac{1}{\sqrt{p}}\boldsymbol{y}^\top \left(\boldsymbol{U}_k \boldsymbol{U}_k^\top + \lambda_k \boldsymbol{I}\right)^{-1} \boldsymbol{U}_k\right)\left(y - \frac{1}{\sqrt{p}}\boldsymbol{y}^\top \left(\boldsymbol{U}_{k'} \boldsymbol{U}_{k'}^\top + \lambda_{k'} \boldsymbol{I}\right)^{-1} \boldsymbol{U}_{k'}\right) \\ &+ \left(y - \frac{1}{\sqrt{p}}\boldsymbol{y}^\top \left(\boldsymbol{U}_k \boldsymbol{U}_k^\top + \lambda_k \boldsymbol{I}\right)^{-1} \boldsymbol{U}_k\right)\left(\frac{1}{\sqrt{p}}\boldsymbol{\xi}_{k'}^\top \left(\boldsymbol{U}_{k'} \boldsymbol{U}_{k'}^\top + \lambda_{k'} \boldsymbol{I}\right)^{-1} \boldsymbol{U}_{k'}\right) \\ &+ \left(y - \frac{1}{\sqrt{p}}\boldsymbol{y}^\top \left(\boldsymbol{U}_{k'} \boldsymbol{U}_{k'}^\top + \lambda_{k'} \boldsymbol{I}\right)^{-1} \boldsymbol{U}_{k'}\right)\left(\frac{1}{\sqrt{p}}\boldsymbol{\xi}_{k}^\top \left(\boldsymbol{U}_{k} \boldsymbol{U}_{k}^\top + \lambda_{k} \boldsymbol{I}\right)^{-1} \boldsymbol{U}_{k}\right) \\ &+ \left(\frac{1}{\sqrt{p}}\boldsymbol{\xi}_{k}^\top \left(\boldsymbol{U}_{k} \boldsymbol{U}_{k}^\top + \lambda_{k} \boldsymbol{I}\right)^{-1} \boldsymbol{U}_{k}\right)\left(\frac{1}{\sqrt{p}}\boldsymbol{\xi}_{k'}^\top \left(\boldsymbol{U}_{k'} \boldsymbol{U}_{k'}^\top + \lambda_{k'} \boldsymbol{I}\right)^{-1} \boldsymbol{U}_{k'}\right) \end{aligned} \tag{125}$$

Taking the expectation value over the readout noise in the training set we get:

$$\mathbb{E}_{\{\boldsymbol{\xi}_1,\ldots,\boldsymbol{\xi}_K\}} E_{kk'} = \left(y - \frac{1}{\sqrt{p}}\boldsymbol{y}^\top \left(\boldsymbol{U}_k \boldsymbol{U}_k^\top + \lambda_k \boldsymbol{I}\right)^{-1} \boldsymbol{U}_k\right)\left(y - \frac{1}{\sqrt{p}}\boldsymbol{y}^\top \left(\boldsymbol{U}_{k'} \boldsymbol{U}_{k'}^\top + \lambda_{k'} \boldsymbol{I}\right)^{-1} \boldsymbol{U}_{k'}\right) \quad (k \neq k') \tag{126}$$

This is identical to $E_{kk'}$ when $\boldsymbol{\xi}_k = 0$ for all k. We may therefore calculate the off-diagonal error terms by setting $\boldsymbol{\xi}_k = 0$ in eq. 122, which gives a problem compatible with the theorem 4 of [31]. To calculate $E_{rr'}$, we appeal to theorem 4.1 and corollary 4.2 of [31]. The following objects defined in 121 are given the following definitions:

$$r(\{\hat{\boldsymbol{w}}_1, \ldots, \hat{\boldsymbol{w}}_K\}) = \frac{1}{2}\sum_{k=1}^K \nu_{kk}\lambda_k |\hat{\boldsymbol{w}}_k|^2 \tag{127}$$

$$\Delta(y, \hat{y}(\boldsymbol{x})) = (y - \hat{y}(\boldsymbol{x}))^2 \tag{128}$$

$$\hat{\ell}(y, \boldsymbol{\mu}) = \frac{1}{2}|\boldsymbol{\mu} - y\boldsymbol{1}|^2 \tag{129}$$

$$\mathcal{Z}^0(y, \mu, \sigma) := \int \frac{P_y^0(y \mid x)\mathrm{d}x}{\sqrt{2\pi\sigma}} \mathrm{e}^{-\frac{(x-\mu)^2}{2\sigma}} = \frac{1}{\sqrt{2\pi(\zeta^2 + \sigma)}} \exp\left(-\frac{(y-\mu)^2}{2(\zeta^2 + \sigma)}\right) \tag{130}$$

$$E_{kk'} = \mathbb{E}_{(y,\boldsymbol{x})}\left[(y - \mu_k)(y - \mu_{k'})\right] \to \mathbb{E}_{(\nu,\boldsymbol{x})}\left[\int dy P_y^g(y|\nu)(y - \mu_k)(y - \mu_{k'})\right] \tag{131}$$

$$= \mathbb{E}_{(\nu,\boldsymbol{x})}\left[(\nu - \mu_k)(\nu - \mu_{k'})\right] + \frac{1}{K^2}\sum_{k=1}^K \eta_k^2 \tag{132}$$

$$= \rho - [\boldsymbol{m}]_k - [\boldsymbol{m}]_{k'} + [\boldsymbol{Q}]_{kk'} + \frac{1}{K^2}\sum_{k=1}^K \eta_k^2 \tag{133}$$

Where ρ, $\boldsymbol{m}$ and $\boldsymbol{Q}$ are defined as in [31]:

$$(\nu, \boldsymbol{\mu}) \sim \mathcal{N}\left(\boldsymbol{0}_{1+K}, \begin{pmatrix} \rho & \boldsymbol{m}^\top \\ \boldsymbol{m} & \boldsymbol{Q} \end{pmatrix}\right) \tag{134}$$

What remains is to determine the values of the order parameters ρ, $\boldsymbol{m}$ and $\boldsymbol{Q}$. We next define the covariance matrices which characterize the feature maps. The feature-feature covariance is:

$$\boldsymbol{\Omega}_{kk'}^{ij} = \mathbb{E}_{\boldsymbol{x}}\left[(\boldsymbol{u}_k(\boldsymbol{x}))_i(\boldsymbol{u}_{k'}(\boldsymbol{x}))_j\right] = \mathbb{1}_{\{1\le i\le N_k, 1\le j\le N_{k'}\}}\left[\boldsymbol{A}_k\left(\boldsymbol{\Sigma}_s+\boldsymbol{\Sigma}_0\right)\boldsymbol{A}_{k'}^\top\right]_{ij} \tag{135}$$

$$= \sqrt{\nu_{kk}\nu_{k'k'}}\mathbb{1}_{\{1\le i\le N_k, 1\le j\le N_{k'}\}}\left[\tilde{\boldsymbol{\Sigma}}_{kk'}\right]_{ij} = \left[\bar{\boldsymbol{\Sigma}}_{kk'}\right]_{ij} \tag{136}$$

where we have defined $\nu_{kk} = \frac{N_k}{p}$, $\tilde{\boldsymbol{\Sigma}}_{kk'} = \frac{1}{\sqrt{\nu_{kk}\nu_{k'k'}}}\boldsymbol{A}_k\left(\boldsymbol{\Sigma}_s+\boldsymbol{\Sigma}_0\right)\boldsymbol{A}_{k'}^\top$, and $\bar{\boldsymbol{\Sigma}}_{kk'} = \sqrt{\nu_{kk}\nu_{k'k'}}\begin{bmatrix}\tilde{\boldsymbol{\Sigma}}_{kk'} & \boldsymbol{0}\\ \boldsymbol{0} & \boldsymbol{0}\end{bmatrix} \in \mathbb{R}^{p\times p}$. Note that while in [31], all $\boldsymbol{\Omega}_{kk}$ must have strictly positive eigenvalues, their result can be easily extended to cover the case where some eigenvalues are zero by a continuity argument.

The feature-label covariance is given by:

$$\left[\hat{\boldsymbol{\Phi}}\right]_k^i = \mathbb{E}_{\boldsymbol{x}}\left[\boldsymbol{u}_k(\boldsymbol{x})\boldsymbol{x}^\top\boldsymbol{\theta}\right]_i = \mathbb{1}_{\{1\le i\le N_k\}}\left[\boldsymbol{A}_k\boldsymbol{\Sigma}_s\boldsymbol{\theta}\right]_i = \left[\bar{\boldsymbol{A}}_k\boldsymbol{\Sigma}_s\boldsymbol{\theta}\right]_i \tag{137}$$

$$[\boldsymbol{\Theta}]_{kk'}^{ij} = \left[\hat{\boldsymbol{\Phi}}\right]_k^i\left[\hat{\boldsymbol{\Phi}}\right]_{k'}^j = \left[\bar{\boldsymbol{A}}_k\boldsymbol{\Sigma}_s\boldsymbol{\theta}\right]_i\left[\bar{\boldsymbol{A}}_{k'}\boldsymbol{\Sigma}_s\boldsymbol{\theta}\right]_j \tag{138}$$

Recalling $\boldsymbol{\omega} := \boldsymbol{Q}^{1/2}\boldsymbol{\xi}$, we can now obtain an explicit form for the proximal $\boldsymbol{h}$:

$$\boldsymbol{h} := \underset{\boldsymbol{u}}{\operatorname{argmin}}\left[\frac{(\boldsymbol{u}-\boldsymbol{\omega})\boldsymbol{V}^{-1}(\boldsymbol{u}-\boldsymbol{\omega})}{2} + \hat{\ell}(y,\boldsymbol{u})\right] = \boldsymbol{V}(\boldsymbol{I}+\boldsymbol{V})^{-1}(\boldsymbol{V}^{-1}\boldsymbol{Q}^{1/2}\boldsymbol{\xi} + y\boldsymbol{1}) \tag{139}$$

The proximal $\boldsymbol{G}$ should not arise in this special case.

Next, we will simplify the saddle-point equations. Simplifying where possible, we may write:

$$\boldsymbol{f} = \boldsymbol{V}^{-1}(\boldsymbol{h}-\boldsymbol{w}) = (\boldsymbol{I}+\boldsymbol{V})^{-1}\left(y\boldsymbol{1}-\boldsymbol{\omega}\right) \tag{140}$$

$$\partial_{\boldsymbol{\omega}}\boldsymbol{f} = -(\boldsymbol{I}+\boldsymbol{V})^{-1} \tag{141}$$

$$\rho = \mathbb{E}_{\boldsymbol{x}}\left[\left(\frac{1}{\sqrt{d}}\boldsymbol{\theta}^\top\boldsymbol{x}\right)^2\right] = \frac{1}{d}\boldsymbol{\theta}^\top\boldsymbol{\Sigma}_s\boldsymbol{\theta} \tag{142}$$

$$\omega_0 \equiv \boldsymbol{m}^\top\boldsymbol{Q}^{-1/2}\boldsymbol{\xi} \tag{143}$$

$$\sigma_0 = \rho - \boldsymbol{m}^\top\boldsymbol{Q}^{-1}\boldsymbol{m} = \frac{1}{d}\boldsymbol{\theta}^\top\boldsymbol{\Sigma}_s\boldsymbol{\theta} - \boldsymbol{m}^\top\boldsymbol{Q}^{-1}\boldsymbol{m} \tag{144}$$

$$\hat{\boldsymbol{V}} = -\alpha\int dy\mathbb{E}_{\boldsymbol{\xi}}\left[Z^0(y,\omega_0,\sigma_0)(-(\boldsymbol{I}+\boldsymbol{V})^{-1})\right] = \alpha(\boldsymbol{I}+\boldsymbol{V})^{-1}\mathbb{E}_{\boldsymbol{\xi}}\left[\underbrace{\int dyZ^0(y,\omega_0,\sigma_0)}_{1}\right] \tag{145}$$

$$\Rightarrow \hat{\boldsymbol{V}} = \alpha(\boldsymbol{I}+\boldsymbol{V})^{-1} \tag{146}$$

We can simplify the prior equation for $\hat{\boldsymbol{V}}$ to the following set of equations:

$$\boldsymbol{V}_{kk} = \frac{1}{p}\operatorname{tr}\left[\bar{\boldsymbol{\Sigma}}_{kk}\left[\nu_{kk}\lambda_k\boldsymbol{I}_p + \hat{\boldsymbol{V}}_{kk}\bar{\boldsymbol{\Sigma}}_{kk}\right]^{-1}\right] \qquad k=1,\dots,K \tag{147}$$

$$\boldsymbol{V}_{kk'} = \frac{1}{p}\operatorname{tr}\left[\bar{\boldsymbol{\Sigma}}_{kk'}\left[\hat{\boldsymbol{V}}_{k'k}\bar{\boldsymbol{\Sigma}}_{k'k}\right]^{-1}\right] \qquad k'\neq k \tag{148}$$

Equations 146 and 148 are solved by setting $\hat{\boldsymbol{V}}_{kk'} = \boldsymbol{V}_{kk'} = 0$ for all $k \neq k'$ so that $\hat{\boldsymbol{V}}_{kk'} = \hat{V}_k \delta_{kk'}$ and $\boldsymbol{V}_{kk'} = V_k \delta_{kk'}$. The diagonal components then satisfy

$$\hat{V}_k = \frac{\alpha}{1 + V_k} \tag{149}$$

$$V_k = \frac{1}{p} \operatorname{tr} \left[\bar{\boldsymbol{\Sigma}}_{kk} \left[\nu_{kk} \lambda_k \boldsymbol{I}_p + \hat{V}_k \bar{\boldsymbol{\Sigma}}_{kk} \right]^{-1} \right] \tag{150}$$

Which may be solved separately for each $k = 1, \ldots, K$. Simplifying the remaining channel equations we have:

$$\hat{\boldsymbol{Q}} = \alpha(\boldsymbol{I} + \boldsymbol{V})^{-1} \left[\left(\zeta^2 + \rho \right) \boldsymbol{1}\boldsymbol{1}^\top - \boldsymbol{1}\boldsymbol{m}^\top - \boldsymbol{m}\boldsymbol{1}^\top + \boldsymbol{Q} \right] (\boldsymbol{I} + \boldsymbol{V})^{-1} \tag{151}$$

$$\Rightarrow \hat{Q}_{kk'} = \frac{1}{\alpha} \hat{V}_k \hat{V}_{k'} \left[\zeta^2 + \rho - m_k - m_{k'} + Q_{kk'} \right] \tag{152}$$

$$\hat{\boldsymbol{m}} = \alpha(\boldsymbol{I} + \boldsymbol{V})^{-1} \boldsymbol{1} \quad \Rightarrow \quad \hat{m}_k = \frac{\alpha}{(1 + V_k)} = \hat{V}_k \tag{153}$$

Simplifying the prior equations we obtain (through some tedious but straightforward algebra):

$$Q_{kk'} = \hat{Q}_{kk'} J_{kk'} + \hat{V}_k \hat{V}_{k'} \Lambda_{kk'} \tag{154}$$

$$m_k = \hat{V}_k R_k \tag{155}$$

Where we have defined:

$$\boldsymbol{G}_k \equiv \nu_{kk} \lambda_k \boldsymbol{I}_p + \hat{V}_k \bar{\boldsymbol{\Sigma}}_{kk} \tag{156}$$

$$J_{kk'} \equiv \frac{1}{p} \operatorname{tr} \left[\bar{\boldsymbol{\Sigma}}_{kk'} \boldsymbol{G}_{k'}^{-1} \bar{\boldsymbol{\Sigma}}_{k'k} \boldsymbol{G}_k^{-1} \right] \tag{157}$$

$$\Lambda_{kk'} \equiv \frac{1}{p} \boldsymbol{\theta}^\top \boldsymbol{\Sigma}_s \bar{\boldsymbol{A}}_k^\top \boldsymbol{G}_k^{-1} \bar{\boldsymbol{\Sigma}}_{kk'} \boldsymbol{G}_{k'}^{-1} \bar{\boldsymbol{A}}_{k'} \boldsymbol{\Sigma}_s \boldsymbol{\theta} \tag{158}$$

$$R_k \equiv \boldsymbol{\theta}^\top \boldsymbol{\Sigma}_s \bar{\boldsymbol{A}}_k^\top \boldsymbol{G}_k^{-1} \bar{\boldsymbol{A}}_k \boldsymbol{\Sigma}_s \boldsymbol{\theta} \tag{159}$$

Solving eq's 154,152 for $\boldsymbol{Q}$, we obtain:

$$Q_{kk'} = \frac{\gamma_{kk'}}{1 - \gamma_{kk'}} \left(\zeta^2 + \rho - m_k - m_{k'} \right) + \frac{\hat{V}_k \hat{V}_{k'}}{1 - \gamma_{kk'}} \Lambda_{kk'} \qquad (k \neq k') \tag{160}$$

Combining these results, we arrive at a formula for $E_{kk'}$:

$$E_{kk'} = \frac{1}{1 - \gamma_{kk'}} \left(\gamma_{kk'} \zeta^2 + \rho - \hat{V}_k R_k - \hat{V}_{k'} R_{k'} + \hat{V}_k \hat{V}_{k'} \Lambda_{kk'} \right) + \frac{1}{K^2} \sum_k \eta_k^2 \qquad (k \neq k') \tag{161}$$

$$\text{where } \gamma_{kk'} \equiv \frac{1}{\alpha} \hat{V}_k \hat{V}_{k'} J_{kk'} \tag{162}$$

Where the order parameters $\hat{V}_k$, $k = 1, \ldots, K$ satisfy the fixed-point equations given by eq's 149, 150.

G.2.2 Diagonal Terms

To calculate E_{rr}, we cannot ignore the presence of readout noise in the loss function. However, as the loss function is separable over the readouts k, we may calculate E_{rr} using the results of [31] in

the special case where $K = 1$, incorporating the readout noise into the label noise. Concretely, we may calculate E_{rr} as the error of a single linear predictor under the same setup as the off-diagonal terms except that in the training set $y^\mu \sim P_y^0(y|\frac{1}{\sqrt{p}}\boldsymbol{\theta}^\top \boldsymbol{x}^\mu)$ where $P_y^0(y|x) = \mathcal{N}(x, \zeta^2 + \eta_k^2)$. This may be recovered from the calculation for the off-diagonal terms by setting $k = k'$ and re-scaling $\zeta^2 \to \zeta^2 + \eta_k^2$, giving:

$$E_{kk} = \frac{1}{1-\gamma_{kk}}\left(\gamma_{kk}(\zeta^2+\eta_r^2) + \rho - 2\hat{V}_k R_k + \hat{V}_k^2 \Lambda_{kk}\right) + \frac{1}{K^2}\sum_k \eta_k^2 \qquad (k \neq k') \tag{163}$$

where the definitions of γ_{kk}, ρ, R_k, and Λ_{kk} can be inherited from the off-diagonal case, as well as the saddle-point equations 149, 150.

G.2.3 Full Error

The results obtained here are equivalent to the results of our main theorem, up to a reshuffling of additive constants η_k^2 among the error terms, and a trivial re-scaling of the order parameters as follows: $V_k \to \frac{1}{\lambda_k} V_k$, $\hat{V}_k \to \lambda_k \hat{V}_k$

H Equicorrelated Data Model

To gain an intuition for the joint effects of correlated data, subsampling, ensembling, feature noise, and readout noise, we simplify the formulas for the generalization error in the following special case:

$$\boldsymbol{\Sigma}_s = s\left[(1-c)\boldsymbol{I}_M + c\mathbf{1}_M\mathbf{1}_M^\top\right] \tag{164}$$

$$\boldsymbol{\Sigma}_0 = \omega^2 \boldsymbol{I}_M \tag{165}$$

Here s is a parameter which sets the overall scale of the data and $c \in [0,1]$ tunes the correlation structure in the data and ω^2 sets the scale of an isotropic feature noise. We consider an ensemble of k readouts, each of which sees a subset of the features. Due to the isotropic nature of the equicorrelated data model and the pairwise decomposition of the generalization error, we expect that the generalization error will depend on the partition of features among the readout neurons through only:

- The number of features sampled by each readout: $N_r \equiv \nu_{rr} M$, for $r = 1, \ldots, k$
- The number of features jointly sampled by each pair of readouts $n_{rr'} \equiv \nu_{rr'} M$ for $r, r' \in \{1, \ldots, k\}$

Here, we have introduced the subsampling fractions $\nu_{rr} = \frac{N_r}{M}$ and the overlap fractions $\nu_{rr'} = \frac{n_{rr'}}{M}$

We will average the generalization error over readout weights drawn randomly from the space perpendicular to $\mathbf{1}_M$, with an added spike along the direction of $\mathbf{1}_M$:

$$\boldsymbol{w}^* = \sqrt{1-\rho^2}\,\mathbb{P}_\perp \boldsymbol{w}_0^* + \rho \mathbf{1}_M \tag{166}$$

$$\boldsymbol{w}_0^* \sim \mathcal{N}(0, \boldsymbol{I}_M) \tag{167}$$

The projection matrix may be written $\mathbb{P}_\perp = \boldsymbol{I}_M - \frac{1}{N}\mathbf{1}_M\mathbf{1}_M^\top$. The two components of the ground truth weights will yield independent contributions to the generalization error in the sense that

$$\langle E_{rr'} \rangle = (1-\rho^2) E_{rr'}(\rho = 0) + \rho^2 E_{rr'}(\rho = 1) \tag{168}$$

Calculating E_{rr} and $E_{rr'}$ is an exercise in linear algebra which is straightforward but tedious. To assist with the tedious algebra, we wrote a Mathematica package which can handle multiplication, addition, and inversion of matrices of symbolic dimension of the specific form encountered in this problem. This form consists of block matrices, where the blocks may be written as $a\delta_{MN}\boldsymbol{I}_M + b\mathbf{1}_M\mathbf{1}_N^\top$, where a, b are scalars and δ_{MN} ensures that there is only a diagonal component for square blocks (when $M = N$). This package is included as supplemental material to this publication.

H.1 Diagonal Terms and Saddle-Point Equations

Here, we solve for the dominant values of q_r and $\hat{q}_r$ and simplify the expressions for E_{rr} in the case of equicorrelated features described above. In this isotropic setting, $E_{rr}, q_r, \hat{q}_r$ will depend on the subsampling only through $N_r = \nu_{rr} M$. We may then write, without loss of generality $\boldsymbol{A}_r = (\boldsymbol{I}_{N_r} \quad \boldsymbol{0}) \in \mathbb{R}^{N_r \times M}$ where $\boldsymbol{0}$ denotes a matrix of all zeros, of the appropriate dimensionality.

We start by simplifying the saddle-point equations 53,54. Expanding $\frac{1}{M} \operatorname{tr}\left(\boldsymbol{G}_r^{-1} \tilde{\boldsymbol{\Sigma}}_{rr}\right)$ and keeping only leading order terms, the saddle-point equations for q_r and $\hat{q}_r$ reduce to:

$$q_r = \frac{\nu_{rr}\left(s(1-c)+\omega^2\right)}{\hat{q}_r(s(1-c)+\omega^2)+\nu_r} \tag{169}$$

$$\hat{q}_r = \frac{\alpha}{\lambda + q_r} \tag{170}$$

Defining $a \equiv s(1-c)+\omega^2$ and solving this system of equations, we find:

$$q_r = \frac{\sqrt{a^2\alpha^2 + 2a\alpha(\lambda - a)\nu_r + (a+\lambda)^2\nu_r^2} - a\alpha + (a-\lambda)\nu_r}{2\nu_r} \tag{171}$$

$$\hat{q}_r = \frac{\sqrt{a^2\alpha^2 + 2a\alpha(\lambda - a)\nu_r + (a+\lambda)^2\nu_r^2} + a\alpha - (a+\lambda)\nu_r}{2a\lambda} \tag{172}$$

We have selected the solution with $q_r > 0$ because self-overlaps must be at least as large as overlaps between different replicas. This solution to the saddle-point equatios can be applied to each of the k readouts.

Next, we calculate E_{rr}. Expanding $\gamma_{rr} \equiv \frac{\alpha}{M\kappa^2} \operatorname{tr}\left[(\boldsymbol{G}^{-1}\tilde{\boldsymbol{\Sigma}})^2\right]$ to leading order in M, we find:

$$\gamma_{rr} = \frac{a^2\alpha\nu_r}{(\lambda+q_r)^2\left(a\hat{q}_r+\nu_r\right)^2} \tag{173}$$

$$\begin{aligned}\langle E_{rr}\rangle_{\mathcal{D},\boldsymbol{w}^*}(\rho=0) =& \frac{1}{1-\gamma_{rr}}\frac{1}{M}\operatorname{tr}\left[\mathbb{P}_\perp\left(\boldsymbol{\Sigma}_s - \frac{2}{\nu_{rr}}\hat{q}_r\boldsymbol{\Sigma}_s\boldsymbol{A}_r^\top\boldsymbol{G}_r^{-1}\boldsymbol{A}_r\boldsymbol{\Sigma}_s + \frac{1}{\nu_{rr}}\hat{q}_r^2\boldsymbol{\Sigma}_s\boldsymbol{A}_r^\top\boldsymbol{G}_r^{-1}\tilde{\boldsymbol{\Sigma}}\boldsymbol{G}_r^{-1}\boldsymbol{A}_r\boldsymbol{\Sigma}_s\right)\mathbb{P}_\perp\right]\\ &+ \frac{\gamma_{rr}}{1-\gamma_{rr}}\zeta^2 + \eta_r^2,\end{aligned} \tag{174}$$

$$\begin{aligned}\langle E_{rr}\rangle_{\mathcal{D},\boldsymbol{w}^*}(\rho=1) =& \frac{1}{1-\gamma_{rr}}\frac{1}{M}\boldsymbol{1}_M^\top\left[\boldsymbol{\Sigma}_s - \frac{2}{\nu_{rr}}\hat{q}_r\boldsymbol{\Sigma}_s\boldsymbol{A}_r^\top\boldsymbol{G}_r^{-1}\boldsymbol{A}_r\boldsymbol{\Sigma}_s + \frac{1}{\nu_{rr}}\hat{q}_r^2\boldsymbol{\Sigma}_s\boldsymbol{A}_r^\top\boldsymbol{G}_r^{-1}\tilde{\boldsymbol{\Sigma}}\boldsymbol{G}_r^{-1}\boldsymbol{A}_r\boldsymbol{\Sigma}_s\right]\boldsymbol{1}_m\\ &+ \frac{\gamma_{rr}}{1-\gamma_{rr}}\zeta^2 + \eta_r^2,\end{aligned} \tag{175}$$

With the aid of our custom Mathematica package, we calculate the traces and contractions in these expressions and expand them to leading order in M, finding:

$$\langle E_{rr}\rangle_{\mathcal{D},\boldsymbol{w}^*}(\rho=0) = \frac{1}{1-\gamma_{rr}}\left(s(1-c)\left(1 - \frac{(1-c)s\hat{q}_r\nu_r\left(\hat{q}_r(s(1-c)+\omega^2)+2\nu_r\right)}{\left(\hat{q}_r(s(1-c)+\omega^2)+\nu_r\right)^2}\right)\right) + \frac{\gamma_{rr}\zeta^2+\eta_r^2}{1-\gamma_{rr}} \tag{176}$$

$$\langle E_{rr}\rangle_{\mathcal{D},\boldsymbol{w}^*}(\rho=1) = \frac{1}{1-\gamma_{rr}}\left(\frac{s(1-c)(1-\nu_{rr})+\omega^2}{\nu_{rr}}\right) + \frac{\gamma_{rr}\zeta^2+\eta_r^2}{1-\gamma_{rr}} \tag{177}$$

In the "ridgeless" limit where $\lambda \to 0$, we obtain:

$$\gamma_{rr} = \frac{4\alpha\nu_{rr}}{(\alpha + \nu_{rr} + |\alpha - \nu_{rr}|)^2} \tag{178}$$

$$\langle E_{rr}(\rho = 0)\rangle_{\mathcal{D},\boldsymbol{w}^*} = \left\{ \begin{array}{ll} \frac{s(1-c)\nu_{rr}}{\nu_{rr}-\alpha}\left(1 + \frac{s\alpha(1-c)(\alpha-2\nu_{rr})}{\nu_{rr}[s(1-c)+\omega^2]}\right) + \frac{\alpha\zeta^2+\nu_{rr}\eta_r^2}{\nu_{rr}-\alpha}, & \text{if } \alpha < \nu_{rr} \\ \frac{s(1-c)\alpha}{\alpha-\nu_{rr}}\left(1 - \frac{s(1-c)\nu_{rr}}{s(1-c)+\omega^2}\right) + \frac{\nu_{rr}\zeta^2+\alpha\eta_r^2}{\alpha-\nu_{rr}}, & \text{if } \alpha > \nu_{rr} \end{array} \right\} \quad (\lambda \to 0) \tag{179}$$

$$\langle E_{rr}(\rho = 1)\rangle_{\mathcal{D},\boldsymbol{w}^*} = \left\{ \begin{array}{ll} \frac{\nu_{rr}}{\nu_{rr}-\alpha}\left(\frac{s(1-c)(1-\nu_{rr})+\omega^2}{\nu_{rr}}\right) + \frac{\alpha\zeta^2+\nu_{rr}\eta_r^2}{\nu_{rr}-\alpha}, & \text{if } \alpha < \nu_{rr} \\ \frac{\alpha}{\alpha-\nu_{rr}}\left(\frac{s(1-c)(1-\nu_{rr})+\omega^2}{\nu_{rr}}\right) + \frac{\nu_{rr}\zeta^2+\alpha\eta_r^2}{\alpha-\nu_{rr}}, & \text{if } \alpha > \nu_{rr} \end{array} \right\} \quad (\lambda \to 0) \tag{180}$$

H.2 Off-Diagonal Terms

In this section, we calculate the off-diagonal error terms $E_{rr'}$ for $r \neq r'$, again making use of our custom Mathematica package to simplify contractions of block matrices of the prescribed form. By the isotropic nature of the equicorrelated data model, $E_{rr'}$ can only depend on the subsampling scheme through ν_{rr}, $\nu_{r'r'}$, and $\nu_{rr'}$. We can thus, without loss of generality, write:

$$\boldsymbol{A}_r = \begin{pmatrix} \boldsymbol{I}_{n_r \times n_r} & \mathbf{0}_{n_r \times n_{r'}} & \mathbf{0}_{n_r \times n_s} & \mathbf{0}_{n_r \times l} \\ \mathbf{0}_{n_s \times n_r} & \mathbf{0}_{n_s \times n_{r'}} & \boldsymbol{I}_{n_s \times n_s} & \mathbf{0}_{n_s \times l} \end{pmatrix} \in \mathbb{R}^{N_r \times M} \tag{181}$$

$$\boldsymbol{A}_{r'} = \begin{pmatrix} \mathbf{0}_{n_{r'} \times n_r} & \boldsymbol{I}_{n_{r'} \times n_{r'}} & \mathbf{0}_{n_{r'} \times n_s} & \mathbf{0}_{n_{r'} \times l} \\ \mathbf{0}_{n_s \times n_r} & \mathbf{0}_{n_s \times n_{r'}} & \boldsymbol{I}_{n_s \times n_s} & \mathbf{0}_{n_s \times l} \end{pmatrix} \in \mathbb{R}^{N_{r'} \times M} \tag{182}$$

where we have defined n_s to be the number of features shared between the readouts, $n_r = N_r - n_s$ and $n_{r'} = N_{r'} - n_s$ and the count of remaining features $l = M - n_r - n_{r'} - n_s$.

Then, to leading order in M, we find:

$$\gamma_{rr'} = \frac{\alpha\nu_{rr'}(s(1-c)+\omega^2)^2}{(\lambda + q_r)(\lambda + q_{r'})\left(\nu_{rr} + (s(1-c)+\omega^2)\hat{q}_r\right)\left(\nu_{r'r'} + (s(1-c)+\omega^2)\hat{q}_{r'}\right)} \tag{183}$$

Averaging $E_{rr'}$ over $\boldsymbol{w}_0^* \sim \mathcal{N}(0, \boldsymbol{I}_M)$, we get:

$$\begin{aligned} \langle E_{rr'}(\mathcal{D})\rangle_{\mathcal{D},\boldsymbol{w}^*}(\rho = 0) =& \frac{\gamma_{rr'}}{1-\gamma_{rr'}}\zeta^2 + \frac{1}{1-\gamma_{rr'}}\left(\frac{1}{M}\operatorname{tr}\left[\mathbb{P}_\perp \boldsymbol{\Sigma}_s \mathbb{P}_\perp\right]\right) \\ &- \frac{1}{M(1-\gamma_{rr'})}\operatorname{tr}\left[\mathbb{P}_\perp \boldsymbol{\Sigma}_s\left(\frac{1}{\nu_{rr}}\hat{q}_r \boldsymbol{A}_r^\top \boldsymbol{G}_r^{-1}\boldsymbol{A}_r + \frac{1}{\nu_{r'r'}}\hat{q}_{r'}\boldsymbol{A}_{r'}^\top \boldsymbol{G}_{r'}^{-1}\boldsymbol{A}_{r'}\right)\boldsymbol{\Sigma}_s \mathbb{P}_\perp\right] \\ &+ \frac{\hat{q}_r\hat{q}_{r'}}{M(1-\gamma_{rr'})}\frac{1}{\sqrt{\nu_{rr}\nu_{r'r'}}}\operatorname{tr}\left[\mathbb{P}_\perp \boldsymbol{\Sigma}_s \boldsymbol{A}_r^\top \boldsymbol{G}_r^{-1}\tilde{\boldsymbol{\Sigma}}_{rr'}\boldsymbol{G}_{r'}^{-1}\boldsymbol{A}_{r'}\boldsymbol{\Sigma}_s \mathbb{P}_\perp\right], \end{aligned} \tag{184}$$

$$\begin{aligned} \langle E_{rr'}(\mathcal{D})\rangle_{\mathcal{D},\boldsymbol{w}^*}(\rho = 1) =& \frac{\gamma_{rr'}}{1-\gamma_{rr'}}\zeta^2 + \frac{1}{M(1-\gamma_{rr'})}\left(\mathbf{1}_M^\top \boldsymbol{\Sigma}_s \mathbf{1}_M\right) \\ &- \frac{1}{M(1-\gamma_{rr'})}\mathbf{1}_M^\top \boldsymbol{\Sigma}_s\left(\frac{1}{\nu_{rr}}\hat{q}_r \boldsymbol{A}_r^\top \boldsymbol{G}_r^{-1}\boldsymbol{A}_r + \frac{1}{\nu_{r'r'}}\hat{q}_{r'}\boldsymbol{A}_{r'}^\top \boldsymbol{G}_{r'}^{-1}\boldsymbol{A}_{r'}\right)\boldsymbol{\Sigma}_s \mathbf{1}_M^\top \\ &+ \frac{\hat{q}_r\hat{q}_{r'}}{M(1-\gamma_{rr'})}\frac{1}{\sqrt{\nu_{rr}\nu_{r'r'}}}\mathbf{1}_M^\top \boldsymbol{\Sigma}_s \boldsymbol{A}_r^\top \boldsymbol{G}_r^{-1}\tilde{\boldsymbol{\Sigma}}_{rr'}\boldsymbol{G}_{r'}^{-1}\boldsymbol{A}_{r'}\boldsymbol{\Sigma}_s \mathbf{1}_M \end{aligned} \tag{185}$$

Calculating these contractions and traces and expanding to leading order in M, we get:

$$
\begin{aligned}
\langle E_{rr'}(\mathcal{D})\rangle_{\mathcal{D},\boldsymbol{w}^*}(\rho=0) = \frac{s(1-c)}{1-\gamma_{rr'}} \Bigg(1 &- \frac{s(1-c)\nu_{rr}\hat{q}_r}{\nu_{rr}+(s(1-c)+\omega^2)\hat{q}_r} - \frac{s(1-c)\nu_{r'r'}\hat{q}_{r'}}{\nu_{r'r'}+(s(1-c)+\omega^2)\hat{q}_{r'}} \\
&+ \frac{s(1-c)(s(1-c)+\omega^2)\nu_{rr'}\hat{q}_r\hat{q}_{r'}}{(\nu_{rr}+(s(1-c)+\omega^2)\hat{q}_r)(\nu_{r'r'}+(s(1-c)+\omega^2)\hat{q}_{r'})} \Bigg) \\
&+ \frac{\gamma_{rr'}}{1-\gamma_{rr'}}\zeta^2
\end{aligned}
\tag{186}
$$

$$
\langle E_{rr'}(\mathcal{D})\rangle_{\mathcal{D},\boldsymbol{w}^*}(\rho=1) = \frac{1}{1-\gamma_{rr'}} \left(\frac{s(1-c)(\nu_{rr'}-\nu_{rr}\nu_{r'r'})+\omega^2\nu_{rr'}}{\nu_{rr}\nu_{r'r'}} \right) + \frac{\gamma_{rr'}}{1-\gamma_{rr'}}\zeta^2 \tag{187}
$$

Taking $\lambda \to 0$ we get the ridgeless limit:

$$
\gamma_{rr'} \to \frac{4\alpha\nu_{rr'}}{(\alpha+\nu_{rr}+|\alpha-\nu_{rr}|)(\alpha+\nu_{r'r'}+|\alpha-\nu_{r'r'}|)} \qquad (\lambda \to 0) \tag{188}
$$

$$
\langle E_{rr'}(\mathcal{D})\rangle_{\mathcal{D},\boldsymbol{w}^*}(\rho=0) = \frac{1}{1-\gamma_{rr'}}F_0(\alpha) + \frac{\gamma_{rr'}}{1-\gamma_{rr'}}\zeta^2 \quad (r \neq r') \tag{189}
$$

where

$$
F_0(\alpha) \equiv \left\{ \begin{array}{ll}
\frac{(c-1)s\big(\nu_r\nu_{r'}((2\alpha-1)(c-1)s+\omega^2)-\alpha^2(c-1)s\nu_{rr'}\big)}{\nu_r((c-1)s-\omega^2)\nu_{r'}}, & \text{if } \alpha \le \nu_{rr} \le \nu_{r'r'} \\
\frac{(c-1)s\big(\nu_{r'}\big((c-1)s\nu_r+(\alpha-1)(c-1)s+\omega^2\big)-\alpha(c-1)s\nu_{rr'}\big)}{((c-1)s-\omega^2)\nu_{r'}}, & \text{if } \nu_{rr} \le \alpha \le \nu_{r'r'} \\
\frac{(c-1)s\big((c-1)s\nu_{r'}-cs\nu_{rr'}+(c-1)s\nu_r-cs+s\nu_{rr'}+s+\omega^2\big)}{(c-1)s-\omega^2}, & \text{if } \nu_{rr} \le \nu_{r'r'} \le \alpha
\end{array} \right\} \tag{190}
$$

$$
\langle E_{rr'}(\mathcal{D})\rangle_{\mathcal{D},\boldsymbol{w}^*}(\rho=1) = \frac{1}{1-\gamma_{rr'}} \left(\frac{s(1-c)(\nu_{rr'}-\nu_{rr}\nu_{r'r'})+\omega^2\nu_{rr'}}{\nu_{rr}\nu_{r'r'}} \right) + \frac{\gamma_{rr'}}{1-\gamma_{rr'}}\zeta^2 \qquad (\lambda \to 0) \tag{191}
$$

H.3 Optimal Regularization

Here, we derive the "locally" optimal regularization which minimizes the prediction error of the ensemble members independently. Equivalently we derive the optimal regularization for a single readout ($k=1$) or the regularization which minimizes the diagonal terms E_{rr} of the generalization errror. By differentiating E_{rr} with respect to the regularization λ, one can show that for the optimal regularization, we will have

$$
\frac{1}{(1-\gamma_{rr})}\frac{dS_r}{d\lambda}\left[\frac{a^2\nu_{rr}}{\alpha(1-\gamma_{rr})}S_r\left((1-\rho^2)I^0_{rr}+\rho^2 I^1_{rr}+\zeta^2+\eta_r^2\right)+(1-\rho^2)s^2(1-c^2)\nu_{rr}(aS_r-1)\right]=0 \tag{192}
$$

It is easy to show that $\frac{dS_r}{d\lambda}$ cannot be equal to 0. Setting the term in brackets to zero and solving for λ, we find with the aid of the computer algebra system Mathematica [54]:

$$
\lambda^\star = a\left(\frac{a\left(\nu_{rr}(\zeta^2+\eta^2)+a\rho^2+s(1-c)\nu_{rr}(1-2\rho^2)\right)}{(1-c)^2\nu_{rr}^2\,(1-\rho^2)\,s^2}-1\right), \qquad (0<c\le 1) \tag{193}
$$

In the limiting case of isotropic data ($c=0$), the optimal regularization reduces to:

$$\lambda^\star = \frac{1}{\nu_{rr}}(\zeta^2 + \eta^2) + s\left(\frac{1}{\nu_{rr}} - 1\right), \qquad (c = 0) \tag{194}$$

We note that while generalization error is discontinuous at $c = 0$, this result can be quickly obtained from eq. 193 by noticing that the formula for the generalization error with $0 < c \leq 1$ reduces to the formula for generalization error with $c = 0$ when we set $\rho = c = 0$ (when $c = 0$, the generalization error does not depend on ρ because there is no special direction in the data).

H.4 Homogeneous Ensembling with Resource Constraints

We make further simplifications in the special case where $\lambda_r = \lambda$, $\eta_r = \eta$, $\nu_{rr} = \frac{1}{k}$ for all $r = 1, \ldots k$. For simplicity, we also set $\nu_{rr'} = 0$ for all $r \neq r'$ so that ensemble members sample mutually exclusive subsets of the features. We will consider both the ridgeless limit $\lambda \to 0$ and the case of "locally optimal" regularization $\lambda = \lambda^*$ (see eq. 193). Concretely, we show here that under these special cases, generalization error has the forms given in eqs. 25, 26.

We start by analyzing the saddle-point equations 111, 112. Note that in this special case the saddle point equations will be identical for all $r = 1, \ldots, k$. We will therefore suppress the r index. Solving this quadratic system of equations explicitly, we encounter the following radical, which we assign variable name x:

$$x \equiv \sqrt{(a\alpha - a\nu + \lambda\nu)^2 + 4a\lambda\nu^2} \tag{195}$$

In order to simplify this radical to begin extracting the factor of $s(1-c)$ that appears in eqs. 25, 26, we define a reduced regularization:

$$\Lambda \equiv \frac{\lambda}{s(1-c)} \tag{196}$$

And substitute Λ, H, W, and Z (recall eq. 24) for λ, η, ω, and ζ, giving

$$x = s(1-c)\mathcal{X}(\alpha, \nu, W, \Lambda) \tag{197}$$

$$\text{where } \mathcal{X}(\alpha, \nu, W, \Lambda) \equiv \sqrt{(\alpha(W+1) + \nu(\Lambda - W - 1))^2 + 4\Lambda\nu^2(W+1)} \tag{198}$$

making the same substitutions, we can then write

$$\hat{q} = [s(1-c)]^{-1}\,\mathcal{Q} \tag{199}$$

$$\text{where} \quad \mathcal{Q} \equiv \frac{2\alpha\nu}{(-\alpha(W+1) + \nu(\Lambda + W + 1) + X)} \tag{200}$$

Continuing to make substitutions in this manner, we arrve at:

$$S = [s(1-c)]^{-1}\mathcal{S} \tag{201}$$

$$\text{where} \quad \mathcal{S} \equiv \frac{\mathcal{Q}}{\nu + \mathcal{Q}(1+W)} \tag{202}$$

Finally, we may express the generalization error in a reasonably compact form. In the special case at hand, the generalization error is written:

$$E_k = \frac{1}{k}E_{rr}(\nu_{rr} = \frac{1}{k}) + \frac{k-1}{k}E_{rr'}(\nu_{rr'} = \frac{1}{k}\delta_{rr'}) \tag{203}$$

Substituting 201 for S in eq. 16, and making further substitutions as necessary, we arrive at:

$$E_{rr}(\nu_{rr} = \frac{1}{k}) = s(1-c)\mathcal{E}_{rr}(k, \alpha, \rho, \Lambda, H, W, Z) \tag{204}$$

where

$$\mathcal{E}_{rr} = \frac{N_1 + N_2}{D} \tag{205}$$

$$N_1 = \alpha(H+1)k + \mathcal{S}(\mathcal{S}(W+1)(\alpha + WZ + Z) - 2\alpha) \tag{206}$$

$$N_2 = \alpha\rho^2(k - \mathcal{S})(W(k+\mathcal{S}) + k + \mathcal{S} - 2) \tag{207}$$

$$D = \alpha k - \mathcal{S}^2(W+1)^2 \tag{208}$$

We also obtain

$$E_{rr'}(\nu_{rr'} = \frac{1}{k}\delta_{rr'}) = s(1-c)\mathcal{E}_{rr'}(k, \alpha, \rho, \Lambda, H, W, Z) \tag{209}$$

where

$$\mathcal{E}_{rr'} = \frac{2\left(\rho^2 - 1\right)\mathcal{S}}{k} - 2\rho^2 + 1 \tag{210}$$

Combining these, we obtain the error of the ensemble as:

$$E_k = s(1-c)\mathcal{E}(k, \alpha, \rho, \Lambda, H, W, Z) \tag{211}$$

where:

$$\mathcal{E} = \frac{1}{k}\mathcal{E}_{rr} + \frac{k-1}{k}\mathcal{E}_{rr'} \tag{212}$$

These result are derived in the Mathematica notebook titled "EquiCorrParameterReduction.nb" included with the available code. It follows from eq. 211 that when $\lambda = 0$ error can be written in the form of eq. 22. It follows from equations 211, 209 that at "locally optimal" regularization λ^*, error can be written in the form of eq. 23. To see this, note that the reduced regularization Λ^* which minimizes E_{rr} will only depend on the other arguments of $\mathcal{E}_{rr}$. Full expresions for the generalization error in the case $\lambda \to 0$ and $\lambda = \lambda^*$ can be found in the mathematica notebooks "EquiCorrPhaseDiagram_ZeroReg.nb" and "EquiCorrPhaseDiagram_LocalReg.nb" included with the available code. These equations are long and difficult to interperet – nor are they directly used in our code – and so are omitted here.

H.4.1 The Intermediate to Noise-Dominated Transition

The transition between the intermediate regime where $1 < k^* < \infty$ and the noise-dominated regime where $k^* = \infty$ can be studied analytically relatively painlessly in the ridgeless limit $\lambda \to 0$. The strategy we employ to determine this phase boundary is to examine the large-k asymptotic expansion of E_k to determine whether the error approaches its asymptotic value from below or above. If E_k approaches E_∞ from below, then k^* must be finite. If E_k approaches E_∞ from above, then k^* may be infinite – however, there is still the possibility of $k^* < \infty$ if E_k is non-monotonic in k. In practice, we check the values of E_k for $k = 1, 2, \ldots, 100$ and $k = \infty$.

Setting $\Lambda = 0$ and expanding E_k around $k = \infty$, we find:

$$\frac{E_k}{s(1-c)} = 1 - \rho^2 + \rho^2 W + \frac{\left((1+W)^2\rho^2 + \alpha(-2 + H + HW + 2\rho^2)\right)}{(1+W)\alpha k} + \mathcal{O}\left(\frac{1}{k^2}\right) \tag{213}$$

Setting the coefficient of k^{-1} to zero, we find the phase boundary as:

$$\alpha = \frac{(1+W)^2\rho^2}{2(1-\rho^2) - H(1+W)} \tag{214}$$

This equation explains the shapes of the boundaries between the intermediate and noise-dominated regions in the phase diagrams of fig. 4 with $\lambda = 0$ (see black dotted lines in panels b, c, d).

An analytical formula for the boundary between the intermediate and noise-dominated regimes at locally optimal regularization $\lambda = \lambda^*$ cannot be easily obtained. To understand why, we can asses the large-k asymptotic expansion of the generalization error at locally optimal regularization:

$$E_k(\lambda = \lambda^*) = s(1-c)\left[1 - \rho^2 + \rho^2 W + \frac{H}{k}\right] + \mathcal{O}\left(\frac{1}{k^2}\right) \tag{215}$$

This shows that at large k, when $H > 0$, error always approaches its asymptotic value from above (recall that when $H = 0$ we always have $k^* = 1$, so that there is no phase boundary unless $H > 0$). Thus, determining the phase boundary requires determining the value of k which minimizes E_k, which is not analytically tractable.

H.5 Infinite Data Limit

In this section we consider the behavior of generalization error in the equicorrelated data model as $\alpha \to \infty$ while keeping the $\lambda \sim \mathcal{O}(1)$. For simplicity, we assume $\nu_{rr'} = 0$ for $r \neq r'$, isotropic features ($c = 0$), no feature noise ($\omega = 0$) and uniform readout noise $\eta_r = \eta$ as in main text Fig. 3. This limit corresponds to data-rich learning, where the number of training examples is large relative to the number of model parameters. In this case, the saddle point equations reduce to:

$$\hat{q}_r \to \frac{\alpha}{\lambda} \tag{216}$$

$$q_r \to \frac{\nu_{rr}\lambda}{\alpha} \tag{217}$$

In this limit, we find that $\gamma_{rr'} \to 0$. Using this, we can simplify the generalization error as follows:

$$E_g = \frac{1}{k^2}\sum_{rr'=1}^{k} E_{rr'} = s\left[1 - \left(2 - \frac{1}{k}\right)\left(\frac{1}{k}\sum_{r=1}^{k}\nu_{rr}\right)\right] + \frac{\eta^2}{k} \tag{218}$$

Interestingly, we find that the readout error in this case depends on the subsampling fractions ν_{rr} only through their mean. Therefore, with infinite data, there will be no distinction between homogeneous and heterogeneous subsampling.

I Theoretical Learning Curves and Optimal Subsampling Phase Diagrams

Here, we provide additional learning curves and phase diagrams of k^* such as those in Fig. 4a,b,c,d, exploring more parameter values for the task-model alignment ρ and the Reduced noises H, W, and Z. Generalization errors are calculated for a homogeneous ensemble of k linear regressors, as described in sections 5, H.4. We also show diagrams of generalization error E_{k^*}.

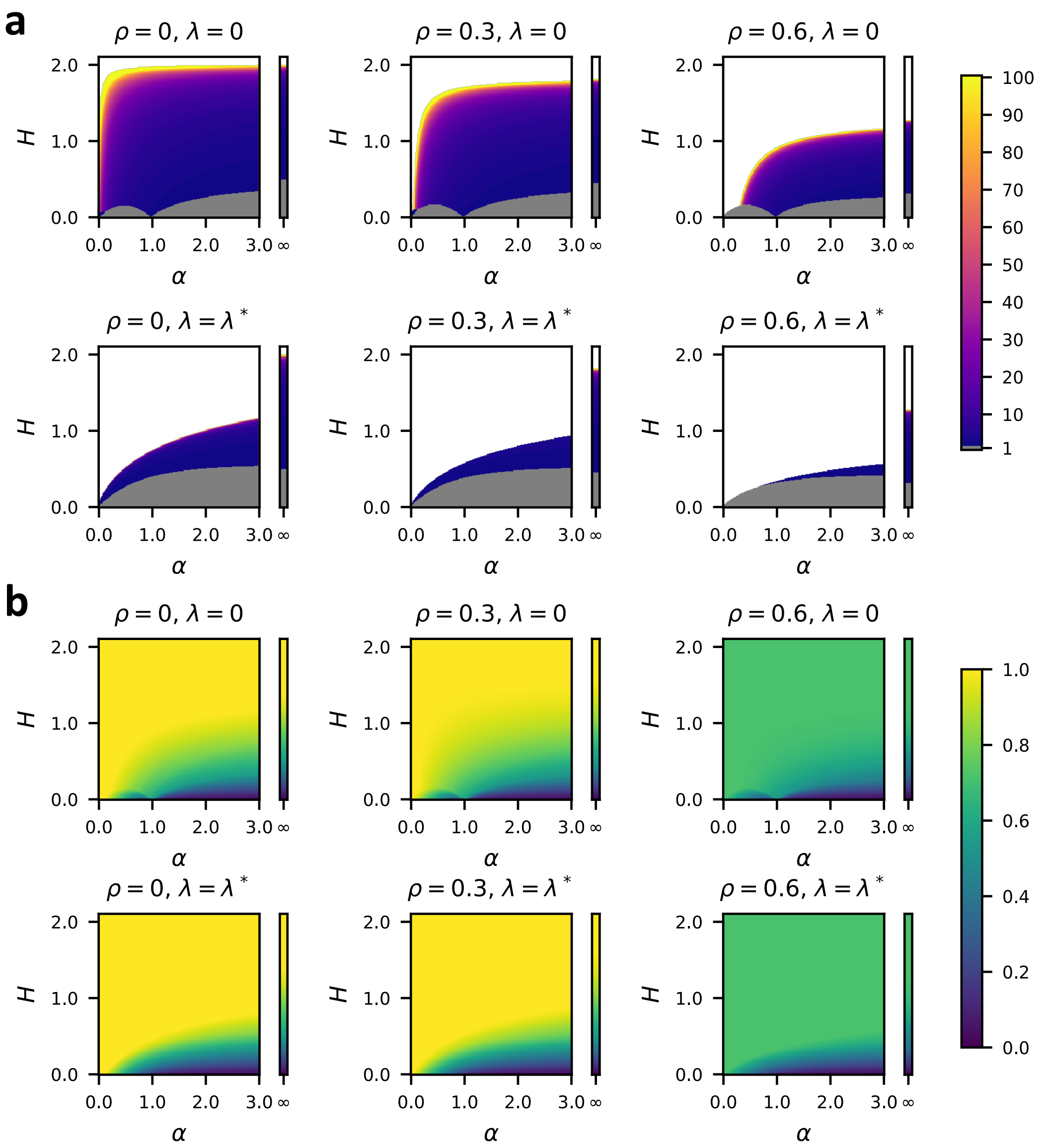

Figure S6: (a) Optimal ensemble size k^* (eqs. 25, 26) in the parameter space of sample size α and reduced readout noise scale H setting $W = Z = 0$. Grey indicates $k^* = 1$ and white indicates $k^* \to \infty$, with intermediate values given by the colorbar. Appended vertical bars show $\alpha \to \infty$. ρ and λ indicated in panel titles. $\lambda = \lambda^*$ denotes the 'locally optimal" regularization (see section H.3) (b) Optimal generalization error E_{k^*} for the same parameter values in (a).

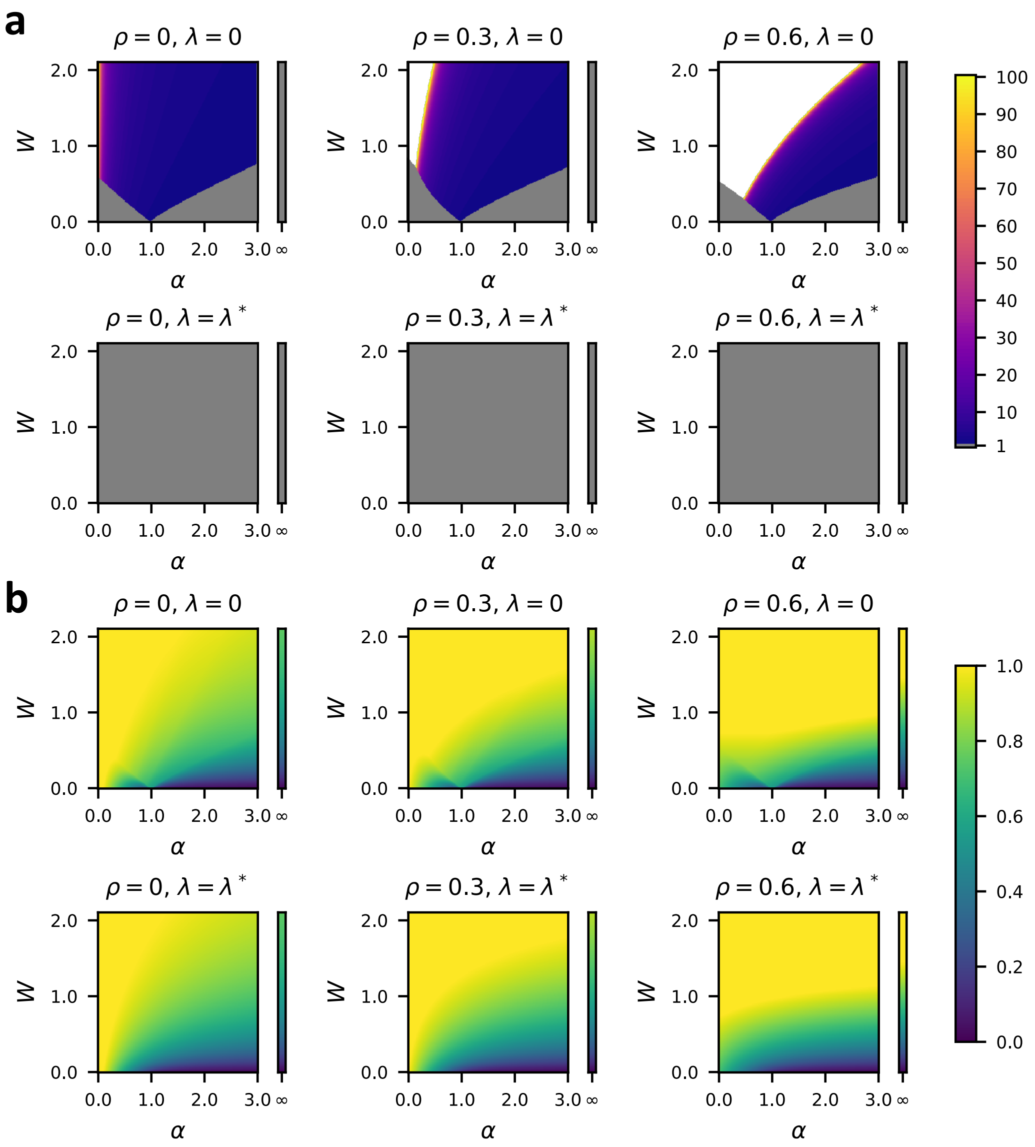

Figure S7: (a) Optimal ensemble size k^* (eqs. 25, 26) in the parameter space of sample size α and reduced feature noise scale W setting $H = Z = 0$. Grey indicates $k^* = 1$ and white indicates $k^* \to \infty$, with intermediate values given by the colorbar. Appended vertical bars show $\alpha \to \infty$. ρ and λ indicated in panel titles. $\lambda = \lambda^*$ denotes the 'locally optimal" regularization (see section H.3) (b) Optimal generalization error E_{k^*} for the same parameter values in (a).

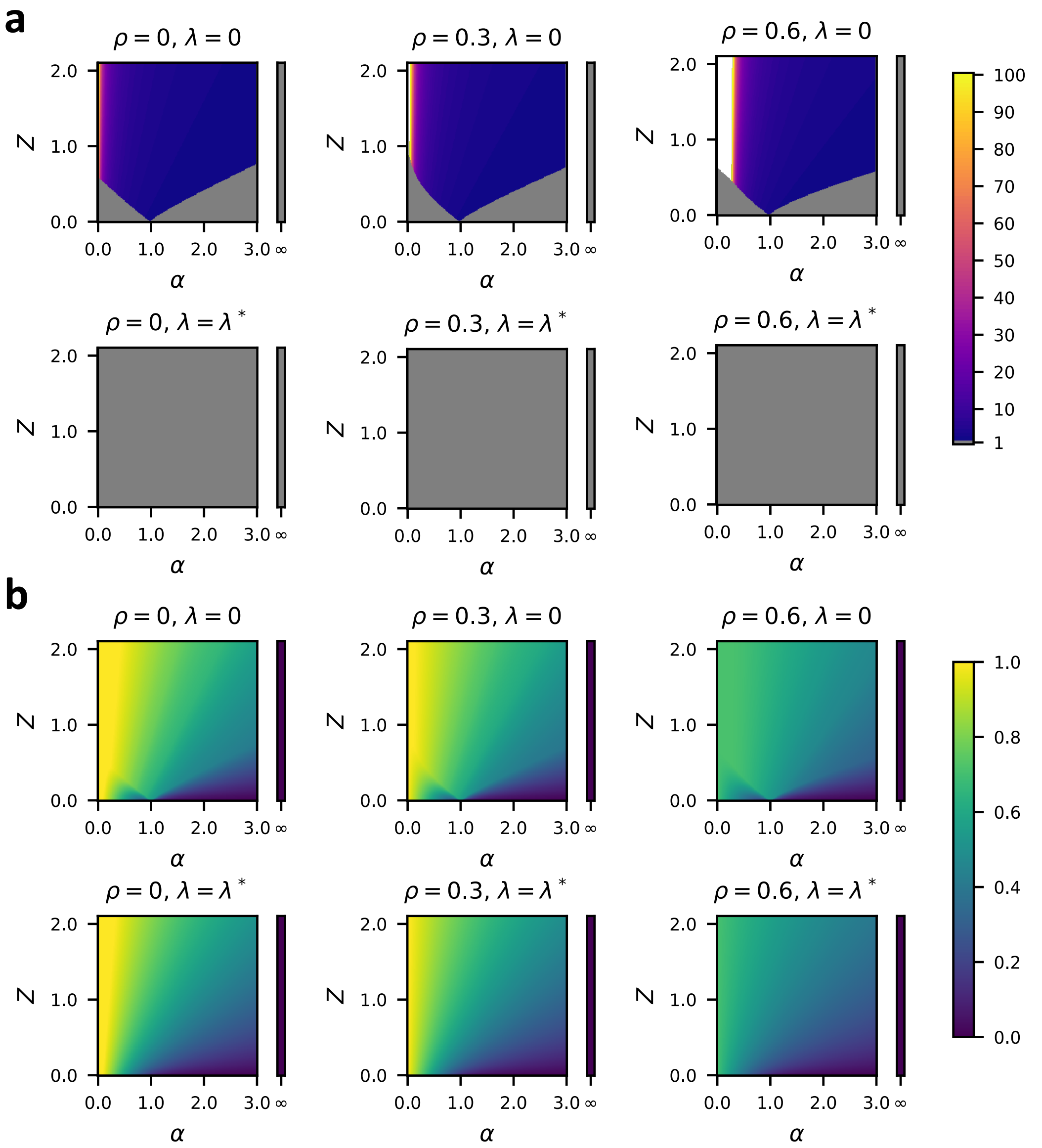

Figure S8: (a) Optimal ensemble size k^* (eqs. 25, 26) in the parameter space of sample size α and reduced label noise scale Z setting $H = W = 0$. Grey indicates $k^* = 1$ and white indicates $k^* \to \infty$, with intermediate values given by the colorbar. Appended vertical bars show $\alpha \to \infty$. ρ and λ indicated in panel titles. $\lambda = \lambda^*$ denotes the 'locally optimal" regularization (see section H.3) (b) Optimal generalization error E_{k^*} for the same parameter values in (a).

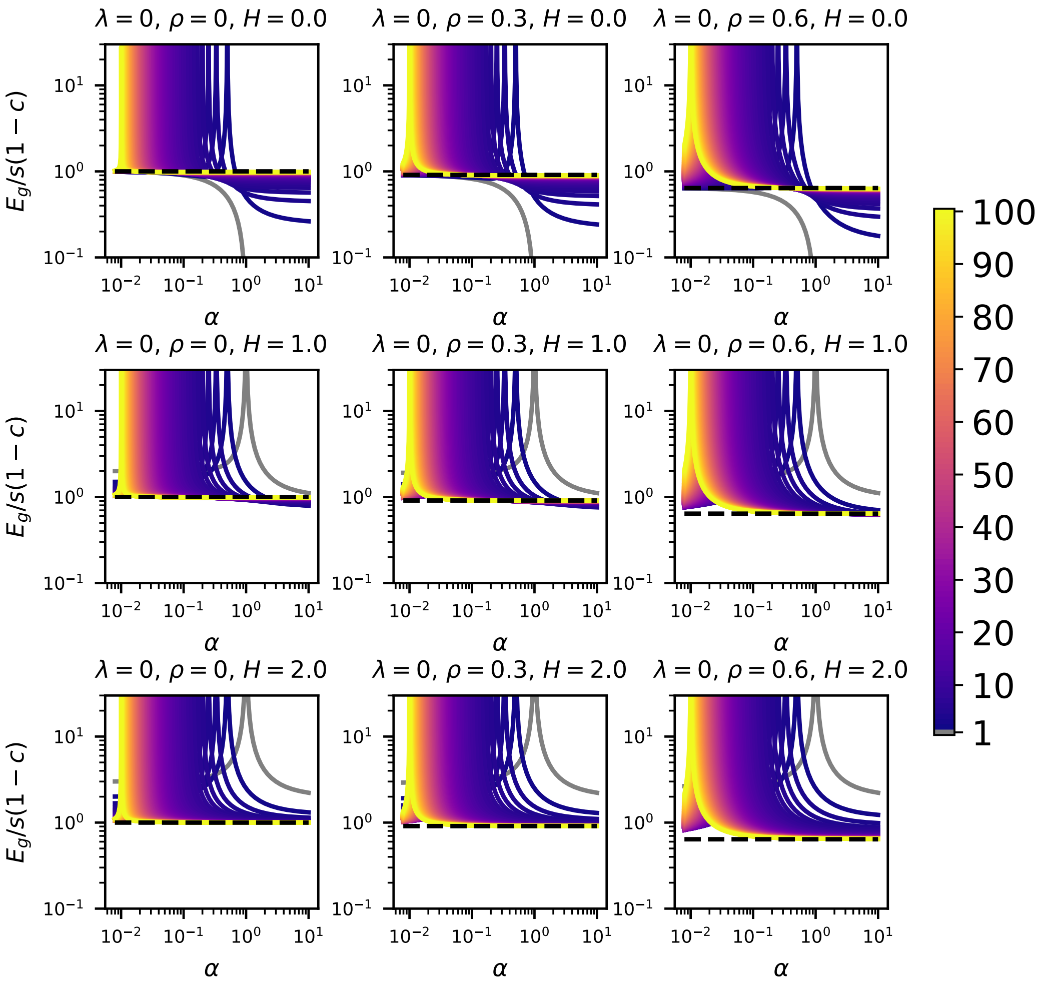

Figure S9: Reduced generalization errors $E_g/s(1-c)$ with $\lambda = 0$ and $W = Z = 0$ (given by eq. 22) for linear ridge ensembles of varying size k. ρ and H values indicated above plots. Grey lines indicate $k = 1$, dashed black lines $k \to \infty$, and intermediate k values by the colorbar.

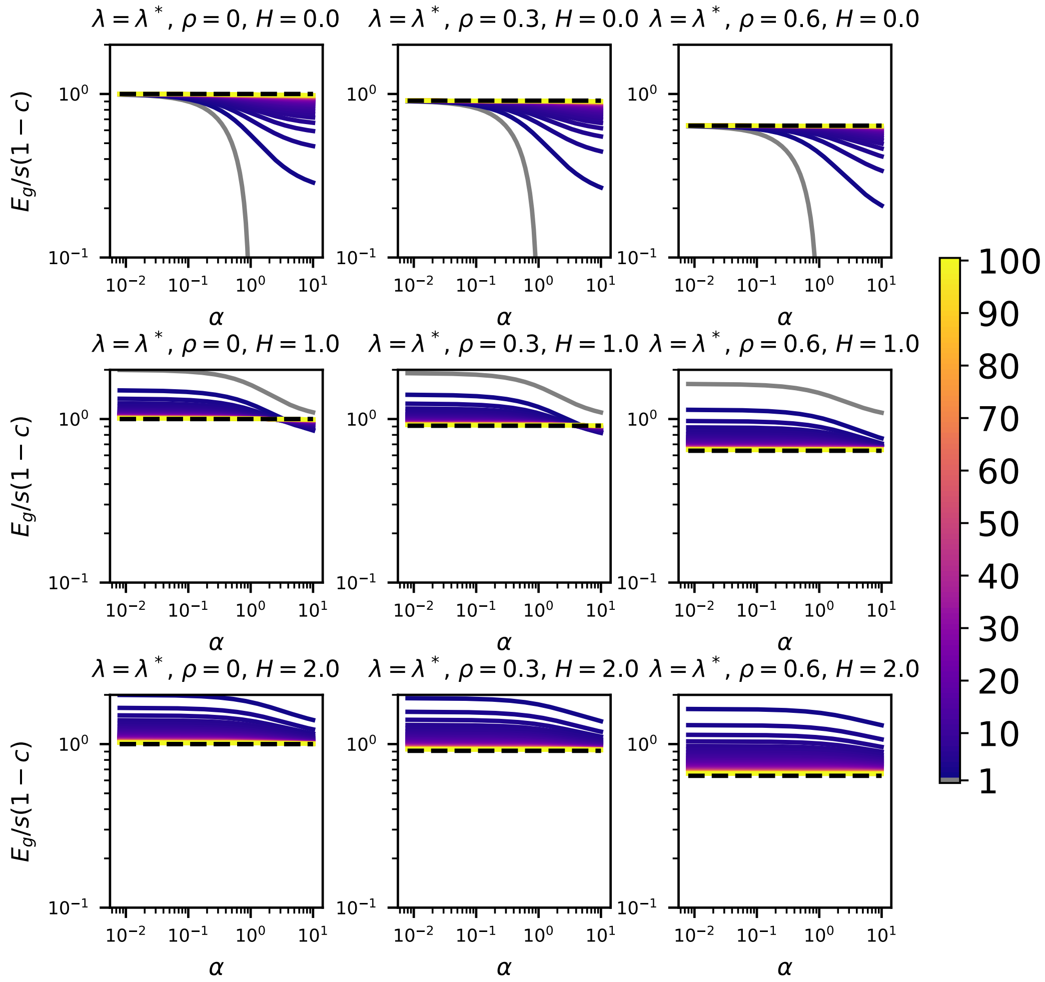

Figure S10: Reduced generalization errors $E_g/s(1-c)$ with $\lambda = \lambda^*$ and $W = Z = 0$ (given by eq. 23) for linear ridge ensembles of varying size k. ρ and H values indicated above plots. Grey lines indicate $k = 1$, dashed black lines $k \to \infty$, and intermediate k values by the colorbar.

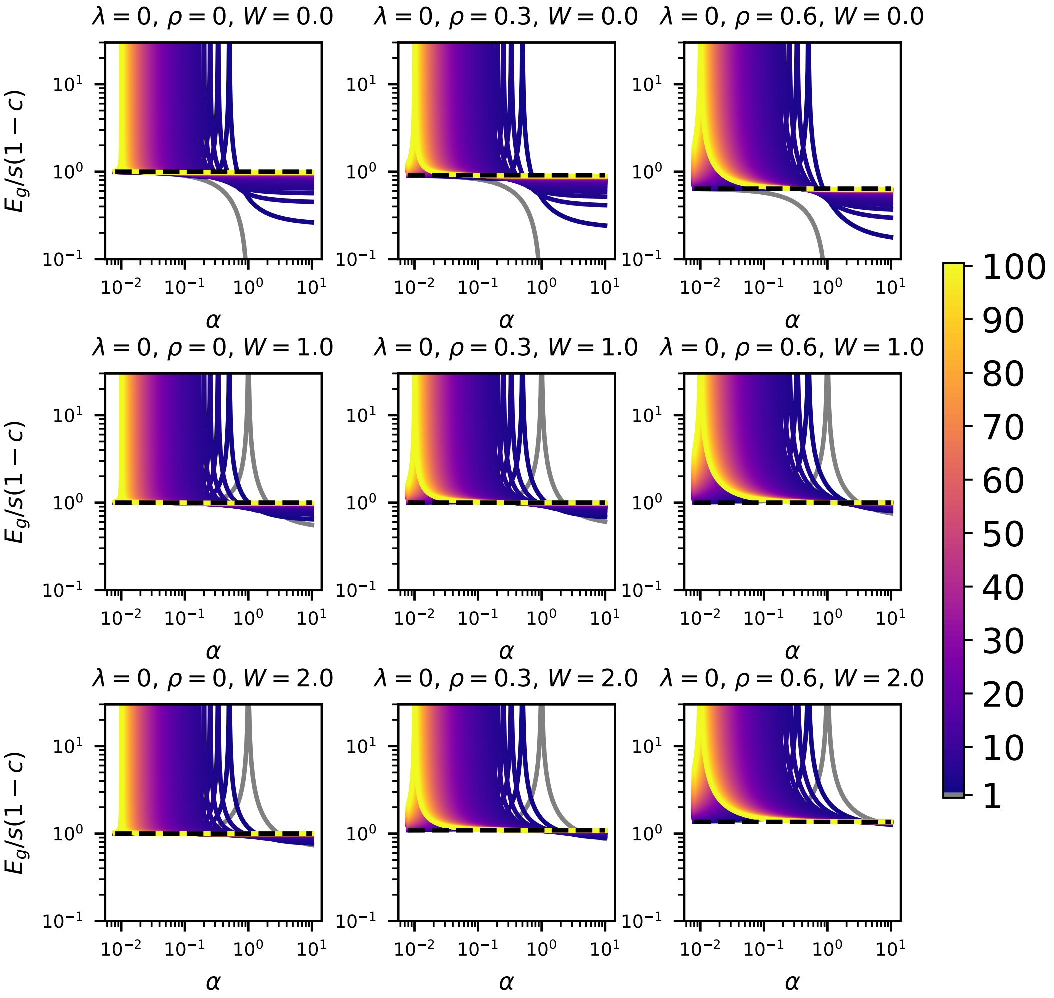

Figure S11: Reduced generalization errors $E_g/s(1-c)$ with $\lambda = 0$ and $H = Z = 0$ (given by eq. 22) for linear ridge ensembles of varying size k. ρ and W values indicated above plots. Grey lines indicate $k = 1$, dashed black lines $k \to \infty$, and intermediate k values by the colorbar.

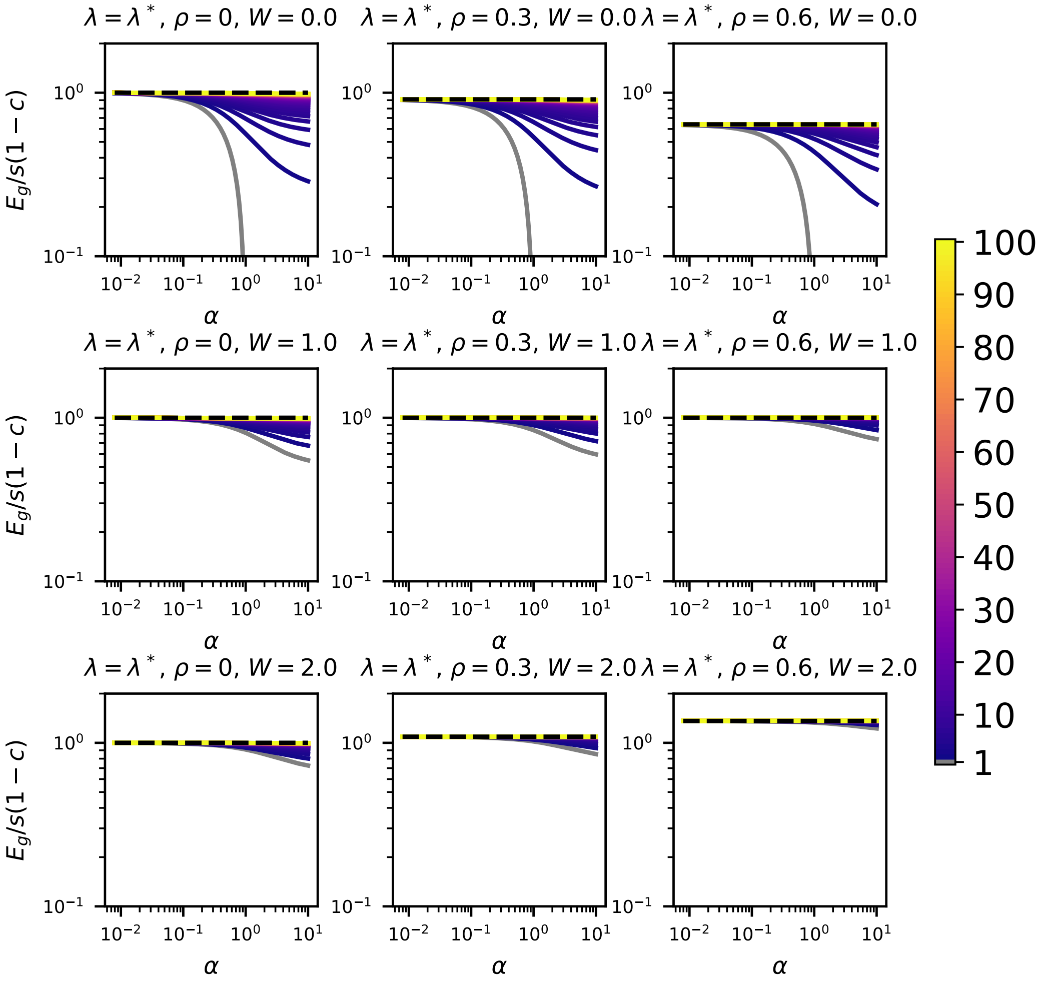

Figure S12: Reduced generalization errors $E_g/s(1-c)$ with $\lambda = \lambda^*$ and $H = Z = 0$ (given by eq. 23) for linear ridge ensembles of varying size k. ρ and W values indicated above plots. Grey lines indicate $k = 1$, dashed black lines $k \to \infty$, and intermediate k values by the colorbar.

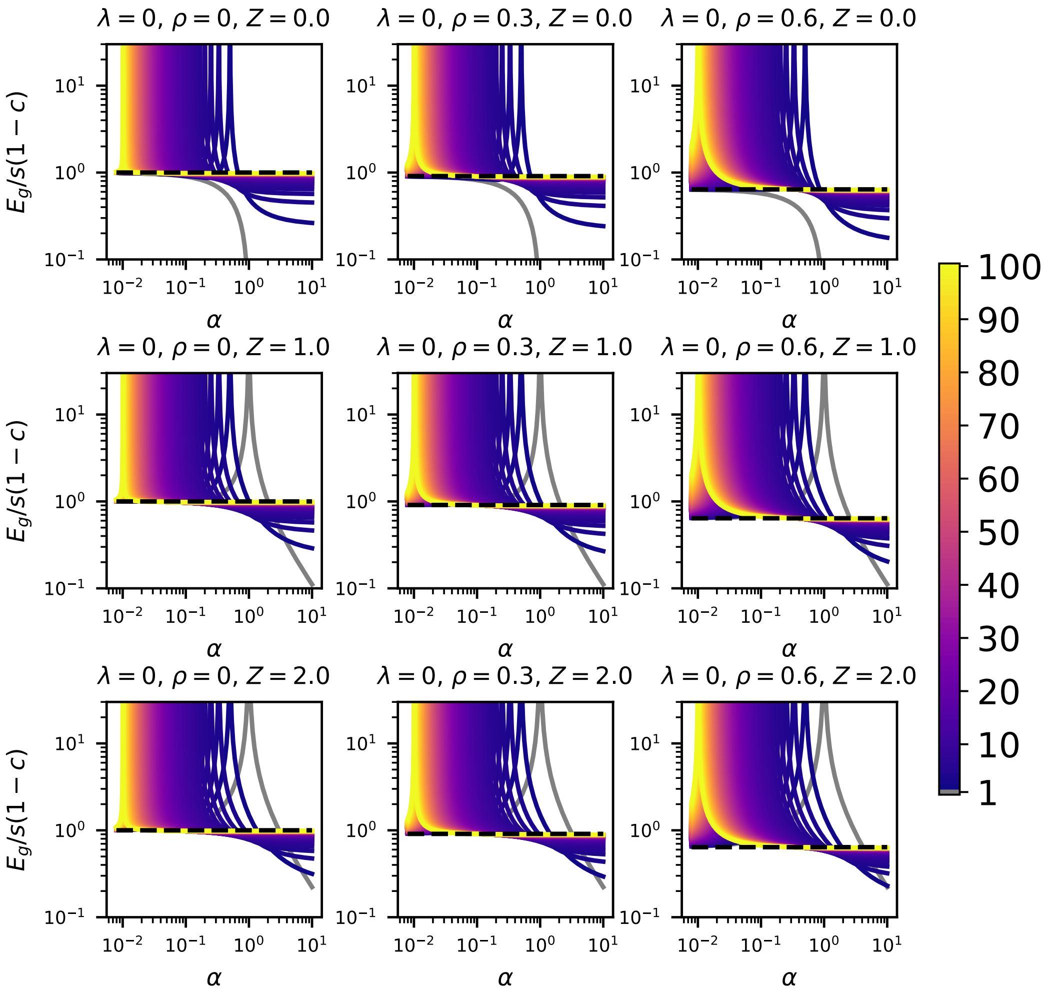

Figure S13: Reduced generalization errors $E_g/s(1-c)$ with $\lambda = 0$ and $H = W = 0$ (given by eq. 22) for linear ridge ensembles of varying size k. ρ and Z values indicated above plots. Grey lines indicate $k = 1$, dashed black lines $k \to \infty$, and intermediate k values by the colorbar

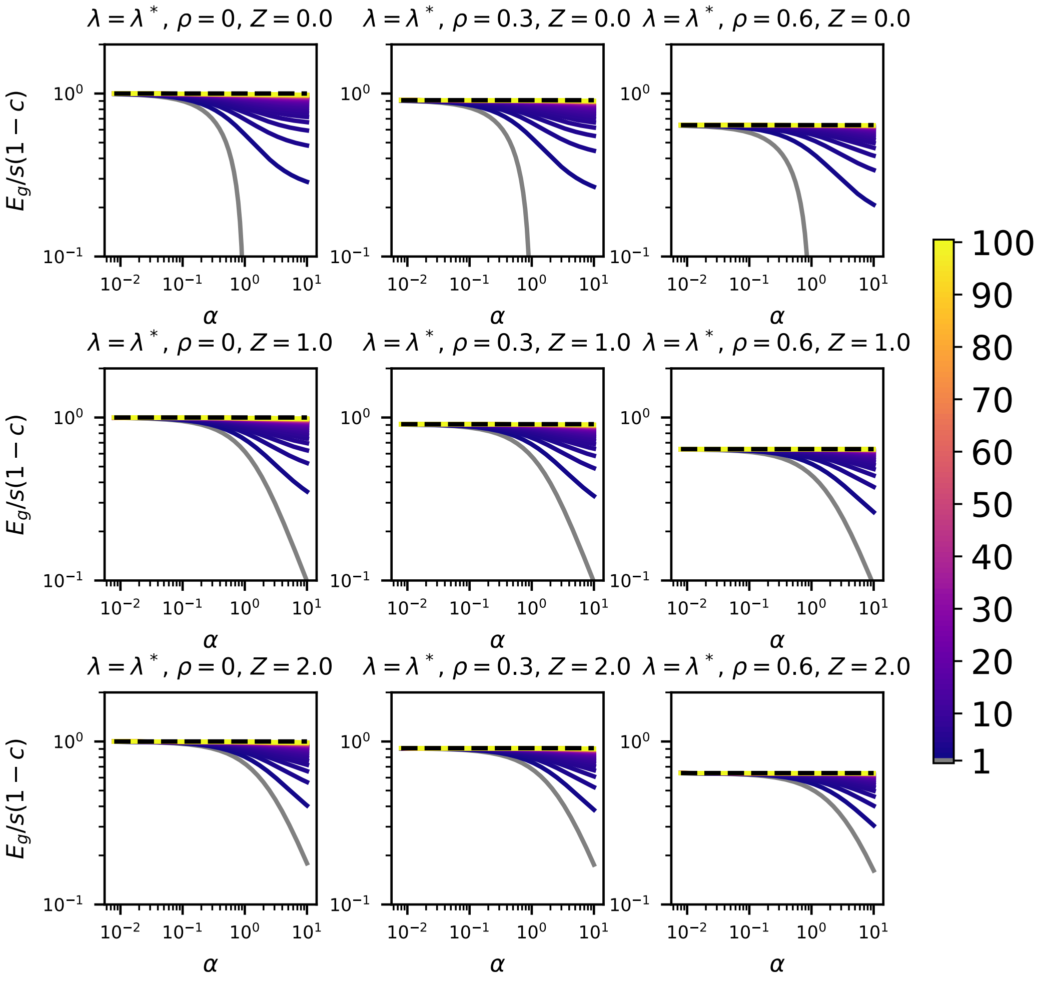

Figure S14: Reduced generalization errors $E_g/s(1-c)$ with $\lambda = \lambda^*$ and $H = W = 0$ (given by eq. 23) for linear ridge ensembles of varying size k. ρ and Z values indicated above plots. Grey lines indicate $k = 1$, dashed black lines $k \to \infty$, and intermediate k values by the colorbar.
